# Supplementary material for: Factors influencing influenza, pneumococcal and shingles vaccine uptake and refusal in older adults: a population-based cross-sectional study in England
Source: BMJ Open. 2023 Mar 15;13(3):e058705. doi: 10.1136/bmjopen-2021-058705 (PMC10030484; doi:10.1136/bmjopen-2021-058705)
Supplement: Supplementary data [file bmjopen-2021-058705supp001.pdf]

Supplement

Table S1: Characteristics of study population - lifestyle and health conditions

Characteristics		Study population	Vaccine uptake		
		Overall	Influenza	Pneumococcal	Shingles[a]
Total	N (row %)	2054463	1711465 (83.3)	1391228 (67.7)	690783 (53.4)
Body mass index	<18.5	36406 (1.8)	31088 (1.8)	25321 (1.8)	9351 (1.4)
	18.5-25	615113 (29.9)	515261 (30.1)	421175 (30.3)	204730 (29.6)
	25-30	754859 (36.7)	641998 (37.5)	528282 (38.0)	273645 (39.6)
	30-35	361993 (17.6)	310919 (18.2)	254754 (18.3)	128653 (18.6)
	35-40	121452 (5.9)	105199 (6.1)	85649 (6.2)	41349 (6.0)
	>=40	48792 (2.4)	42948 (2.5)	34151 (2.5)	15061 (2.2)
	Not recorded	115848 (5.6)	64052 (3.7)	41896 (3.0)	17994 (2.6)
Smoking	Non-smoker	1143669 (55.7)	955785 (55.8)	773504 (55.6)	383407 (55.5)
	Ex-smoker	712384 (34.7)	618783 (36.2)	516754 (37.1)	265778 (38.5)
	Current smoker	177685 (8.6)	132076 (7.7)	98773 (7.1)	40903 (5.9)
	Not recorded	20725 (1.0)	4821 (0.3)	2197 (0.2)	695 (0.1)
Health conditions	Asthma	254110 (12.4)	235822 (13.8)	162658 (11.7)	89598 (13.0)
	Chronic obstructive pulmonary disease	160907 (7.8)	150873 (8.8)	66827 (4.8)	52655 (7.6)
	Type-1 diabetes	6253 (0.3)	5908 (0.3)	4243 (0.3)	1882 (0.3)
	Type-2 diabetes	353860 (17.2)	327748 (19.2)	183136 (13.2)	120912 (17.5)
	Hypertension	1013241 (49.3)	901041 (52.6)	559319 (40.2)	360378 (52.2)
	Dementia	86868 (4.2)	81151 (4.7)	8622 (0.6)	10989 (1.6)
	Parkinson's disease	20720 (1.0)	18825 (1.1)	4635 (0.3)	5467 (0.8)
	Epilepsy	38404 (1.9)	33738 (2.0)	19335 (1.4)	10874 (1.6)
	Cerebral palsy	1041 (0.1)	929 (0.1)	598 (0.0)	233 (0.0)
	Learning disability	39959 (1.9)	36644 (2.1)	9192 (0.7)	9897 (1.4)
	Severe mental illness	243791 (11.9)	210885 (12.3)	133322 (9.6)	73294 (10.6)
	Coronary heart disease	294490 (14.3)	273488 (16.0)	153850 (11.1)	101948 (14.8)
	Atrial fibrillation	196503 (9.6)	180461 (10.5)	53438 (3.8)	55647 (8.1)
	Congestive cardiac failure	85674 (4.2)	79600 (4.7)	19891 (1.4)	20144 (2.9)
	Congenital heart disease	14739 (0.7)	13500 (0.8)	6590 (0.5)	4938 (0.7)
	Immunosuppression	17339 (0.8)	16188 (0.9)	8622 (0.6)	3445 (0.5)

[a]Percentage calculated using denominator of shingles eligible population, n = 1,294,176. [b] Comorbidities diagnosed prior to vaccinations in those vaccinated. Percentages are column percentages unless otherwise indicated. SD: standard deviation.

Figure S1: Directed acyclic graphs (DAGs) modelling exposures and corresponding outcomes. DAGs were used to map out the relationships between exposure and outcome of interest, and how they were related to other covariates to evaluate which variables were considered a confounder and would need to be adjusted for in the regression models.

Interpretation of DAGs
Green circles denote exposure and blue circle with "I" denote outcome.
White circles denote adjusted covariates while other blue circles denote variables not for adjustment in each model.

Model 1:
Exposure: Ethnicity
Outcome: Vaccination uptake/refusal
Confounder adjustment: None (no other variables were identified as a confounder for the association between ethnicity and vaccine uptake/refusal)

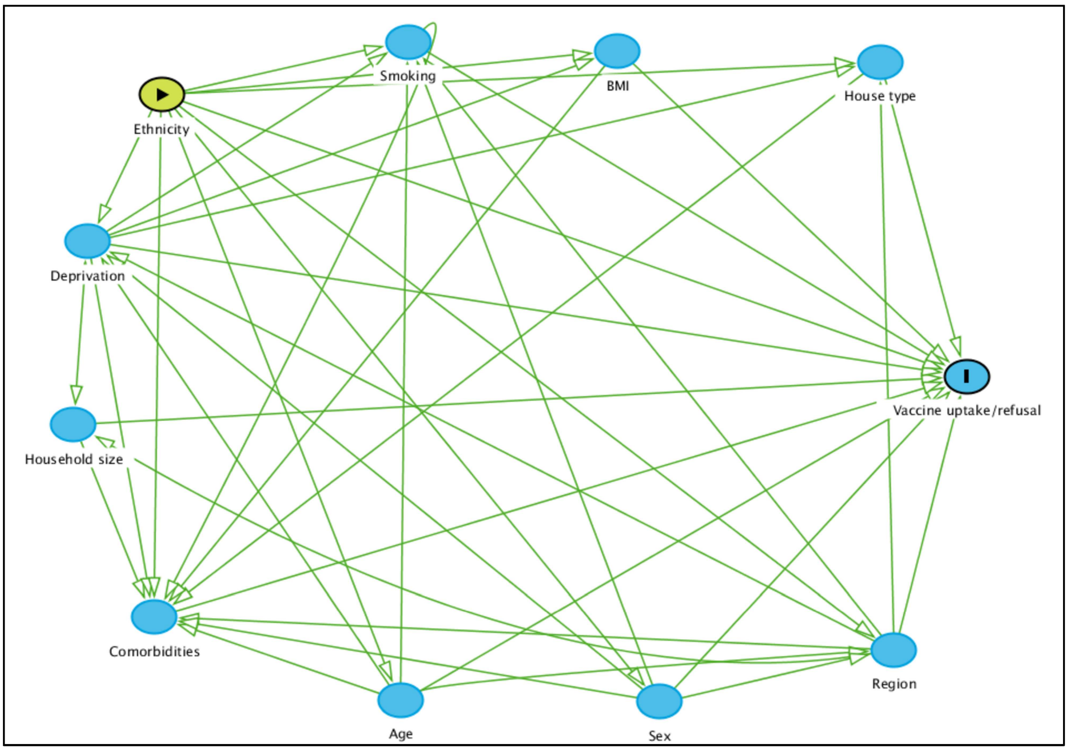

Model 2:
Exposure: Deprivation
Outcome: Vaccination uptake/refusal
Confounder adjustment: age, sex, region, ethnicity, household size (identified as confounders for the association between deprivation and vaccine uptake/refusal)

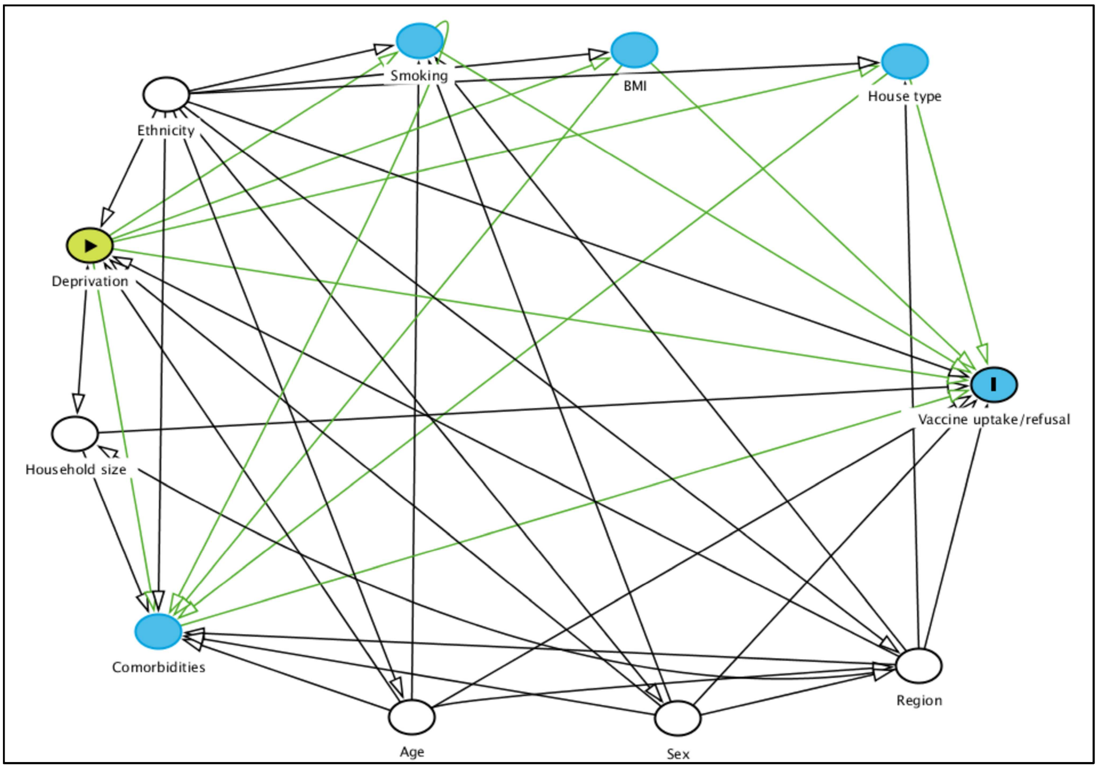

Model 3:
Exposure: Household size
Outcome: Vaccination uptake/refusal
Confounder adjustment: age, sex, region, ethnicity, deprivation (identified as confounders for the association between household size and vaccine uptake/refusal)

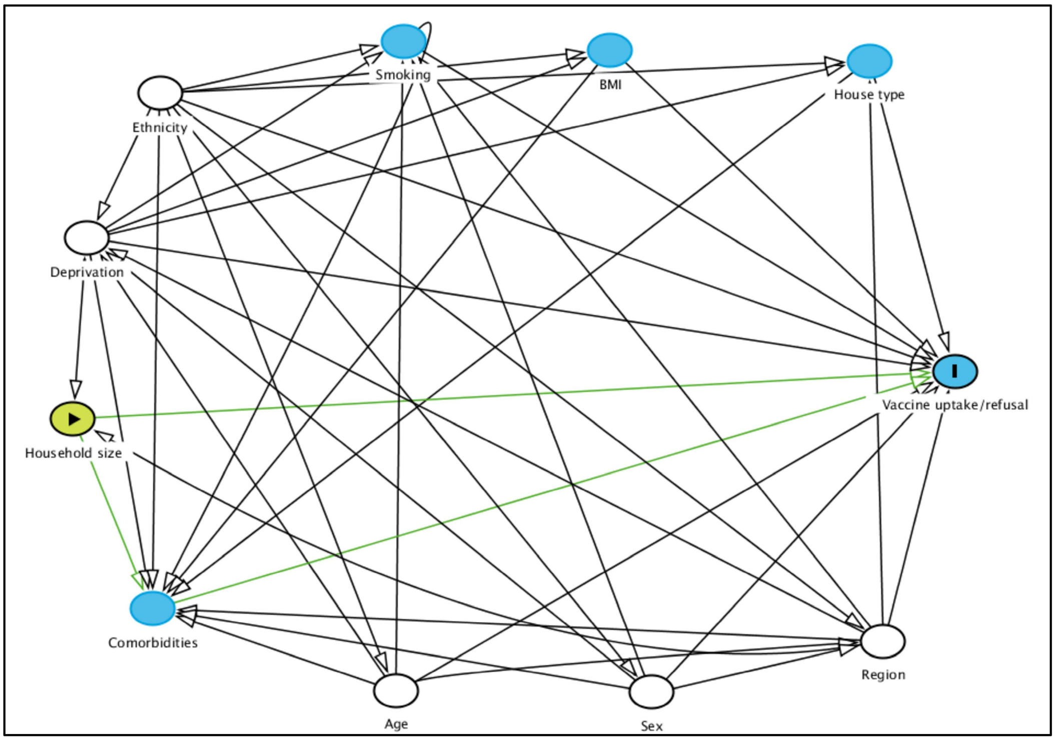

Model 4:
Exposure: Health conditions (comorbidities)
Outcome: Vaccination uptake/refusal
Confounder adjustment: age, sex, region, ethnicity, deprivation, household size, house type, smoking, BMI (identified as confounders for the association between health conditions (comorbidities) and vaccine uptake/refusal)

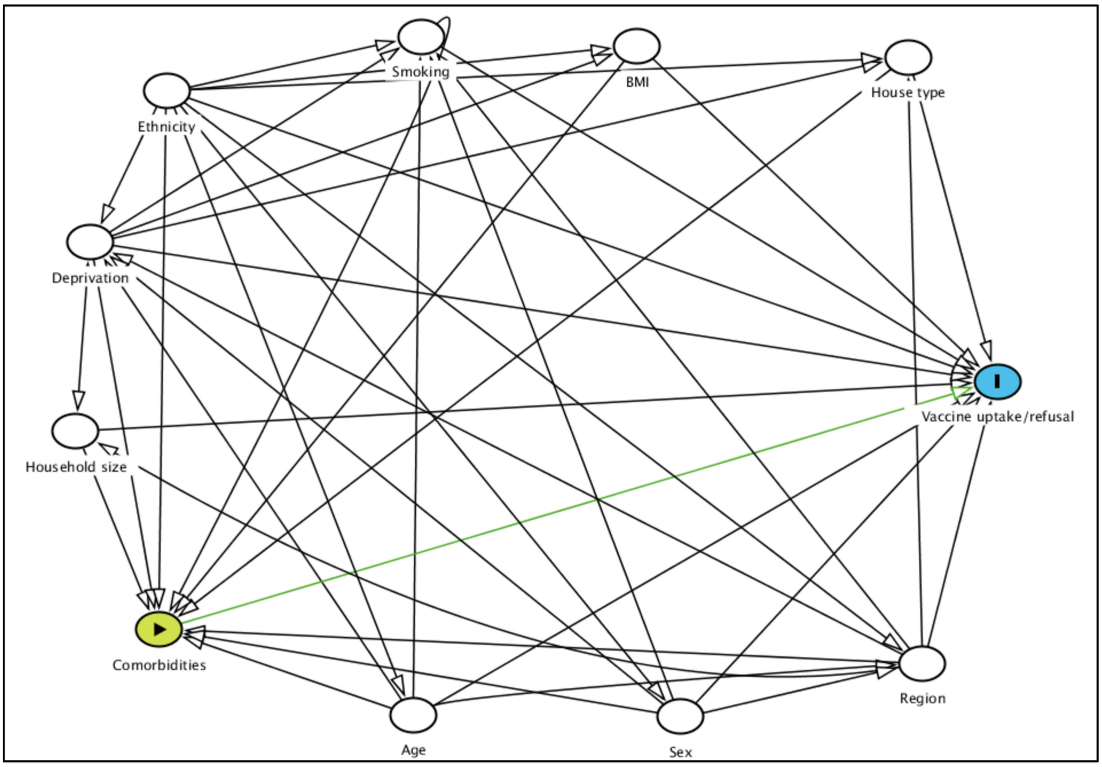

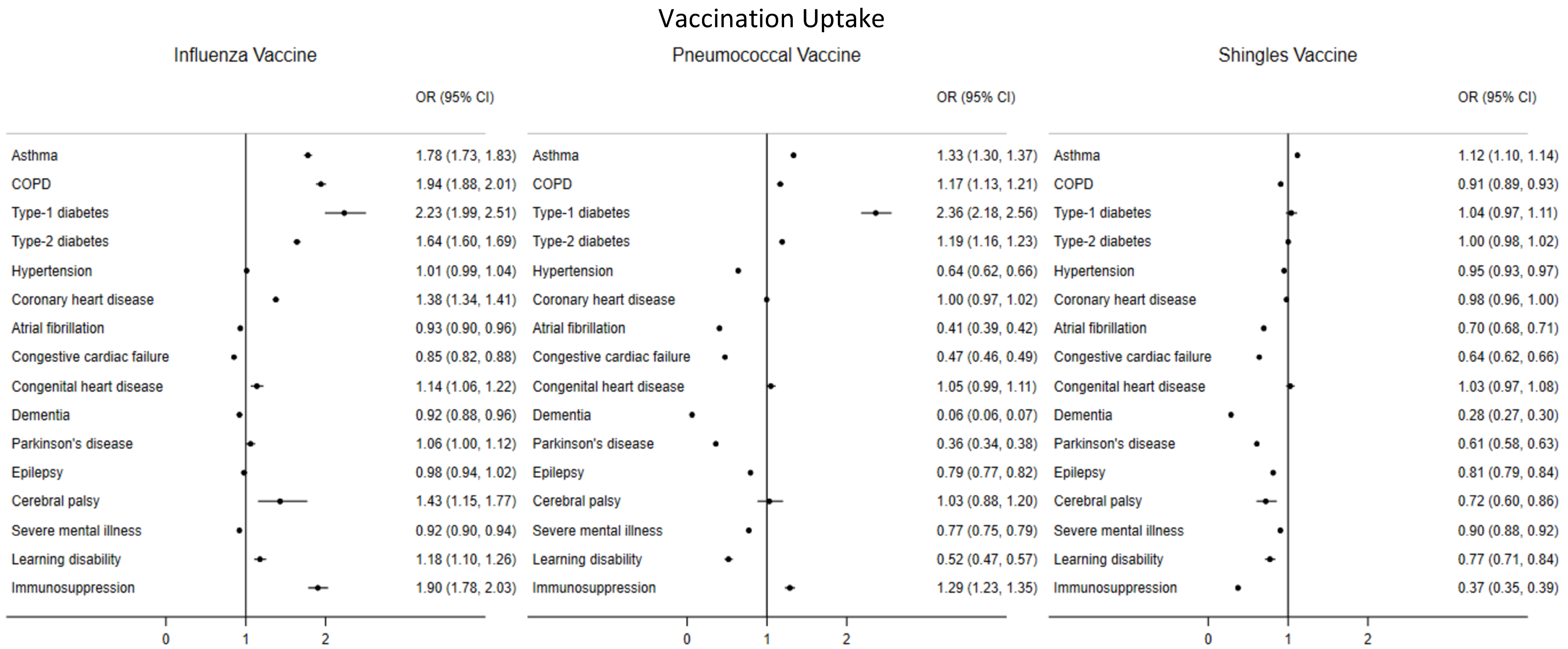

Figure S2: Associations of vaccine uptake and specific health conditions.

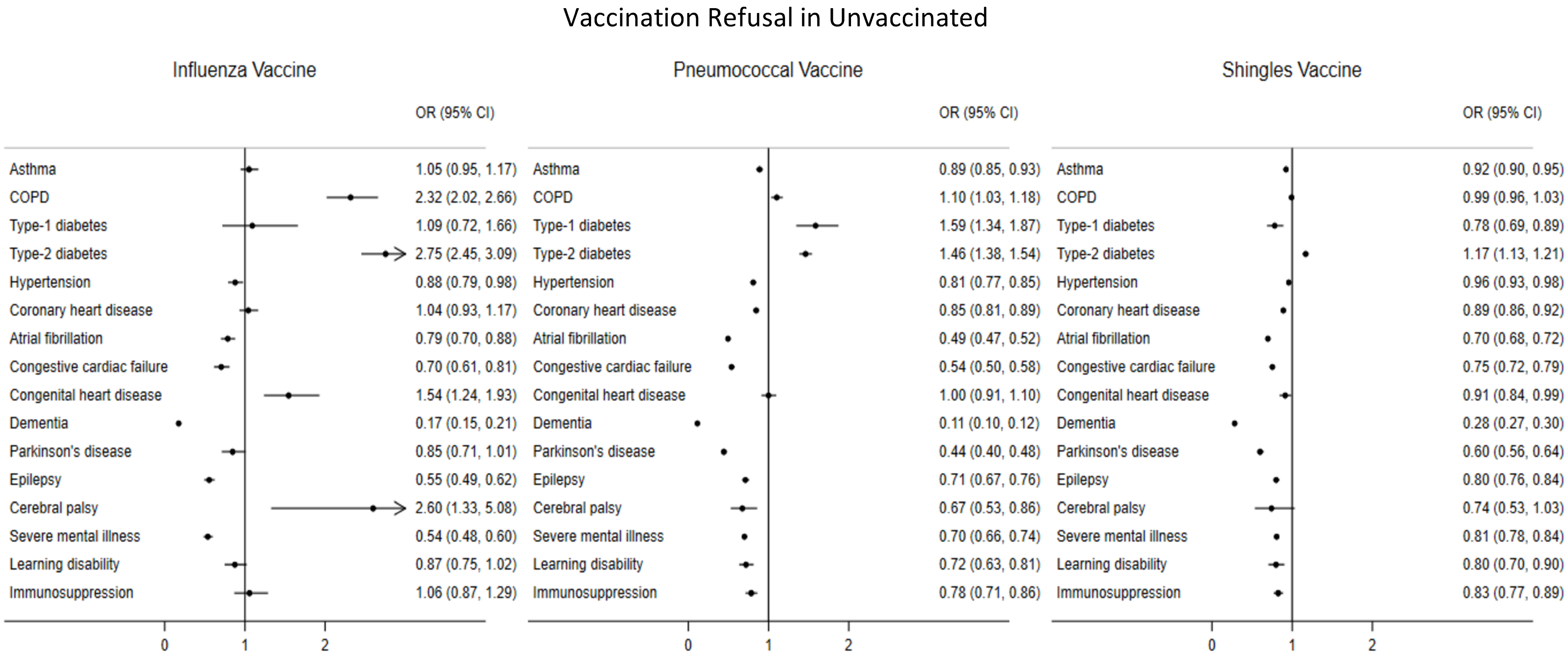

Figure S3: Associations of vaccine refusal in unvaccinated and specific health conditions.

Figure S4: Interaction analyses for vaccine uptake: ethnicity and deprivation

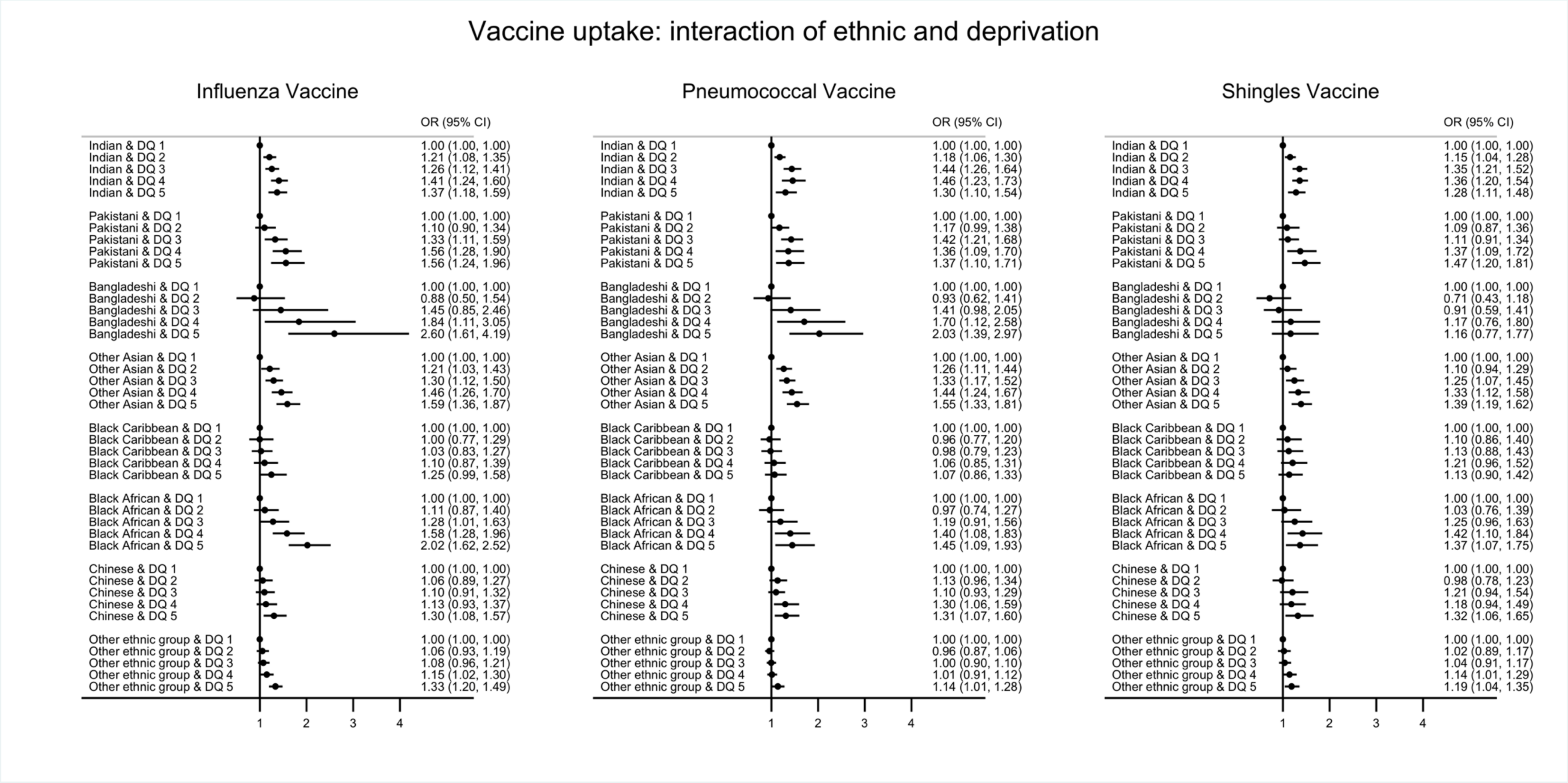

Interaction p values were <0.01 for uptake of each vaccines evaluated; DQ: deprivation quintile

Figure S5: Interaction analyses for vaccine uptake: ethnicity and household size

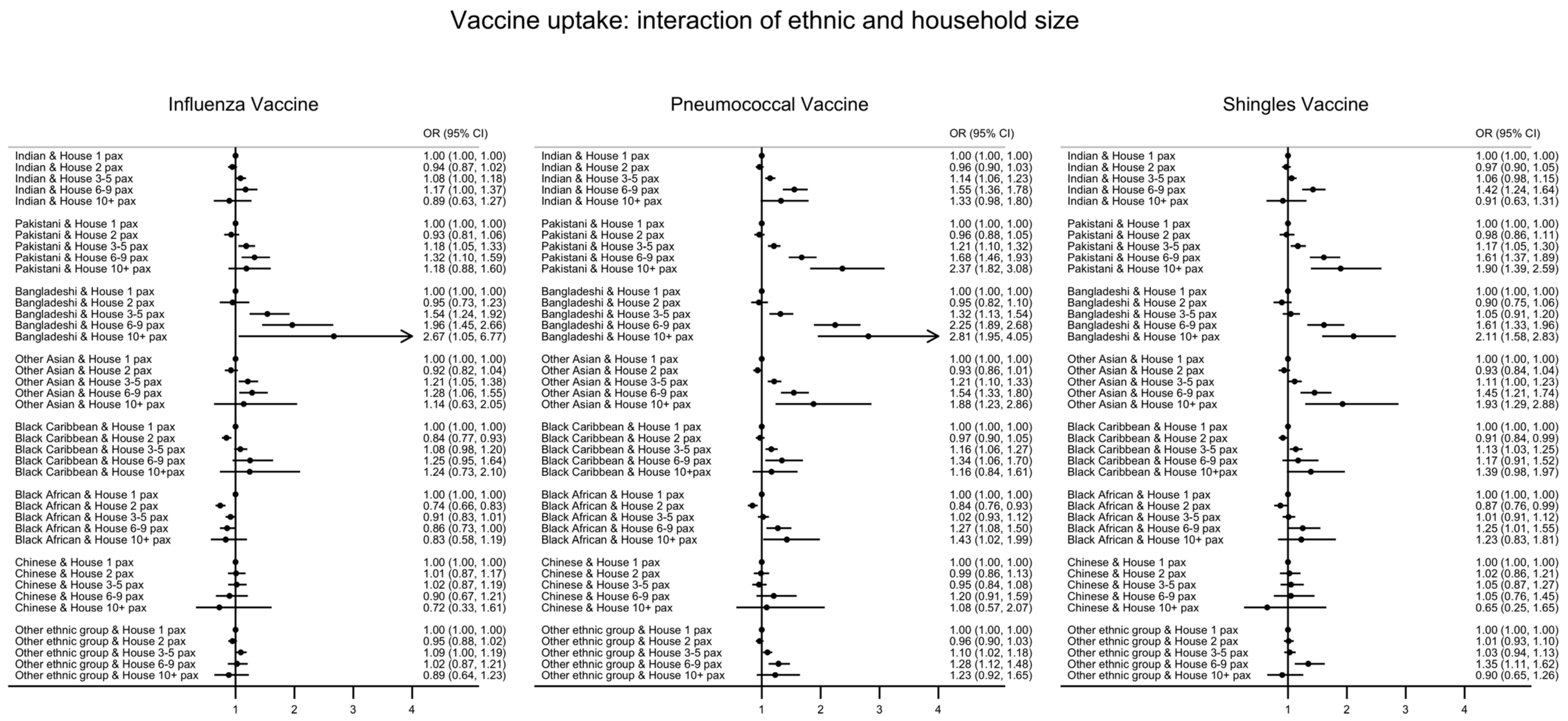

Interaction p values were <0.01 for uptake of each vaccines evaluated.

Figure S6: Interaction analyses for vaccine uptake: ethnicity and number of health conditions

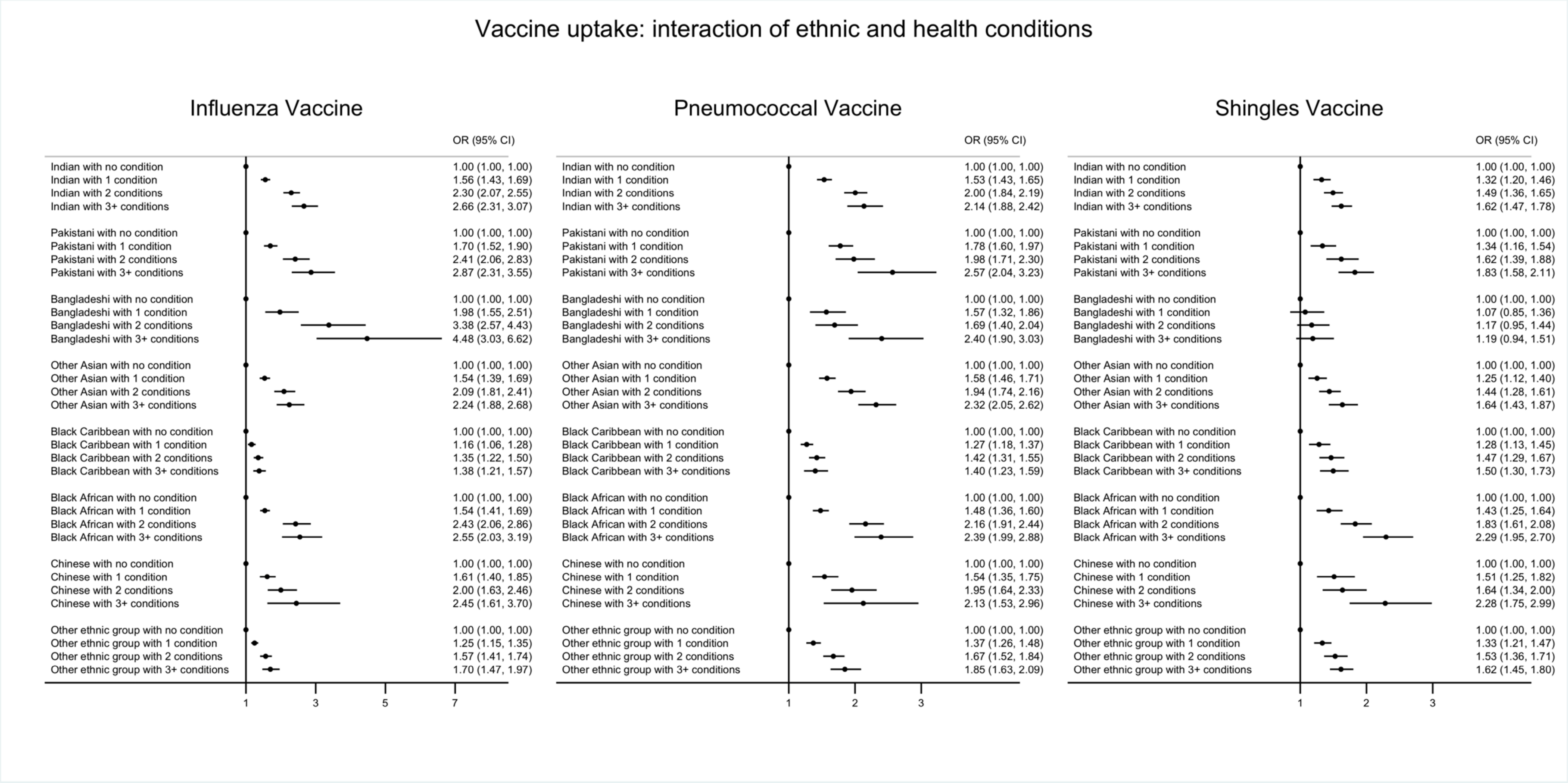

Interaction p values were <0.01 for uptake of each vaccines evaluated.

Vaccination Uptake (complete-case analysis)

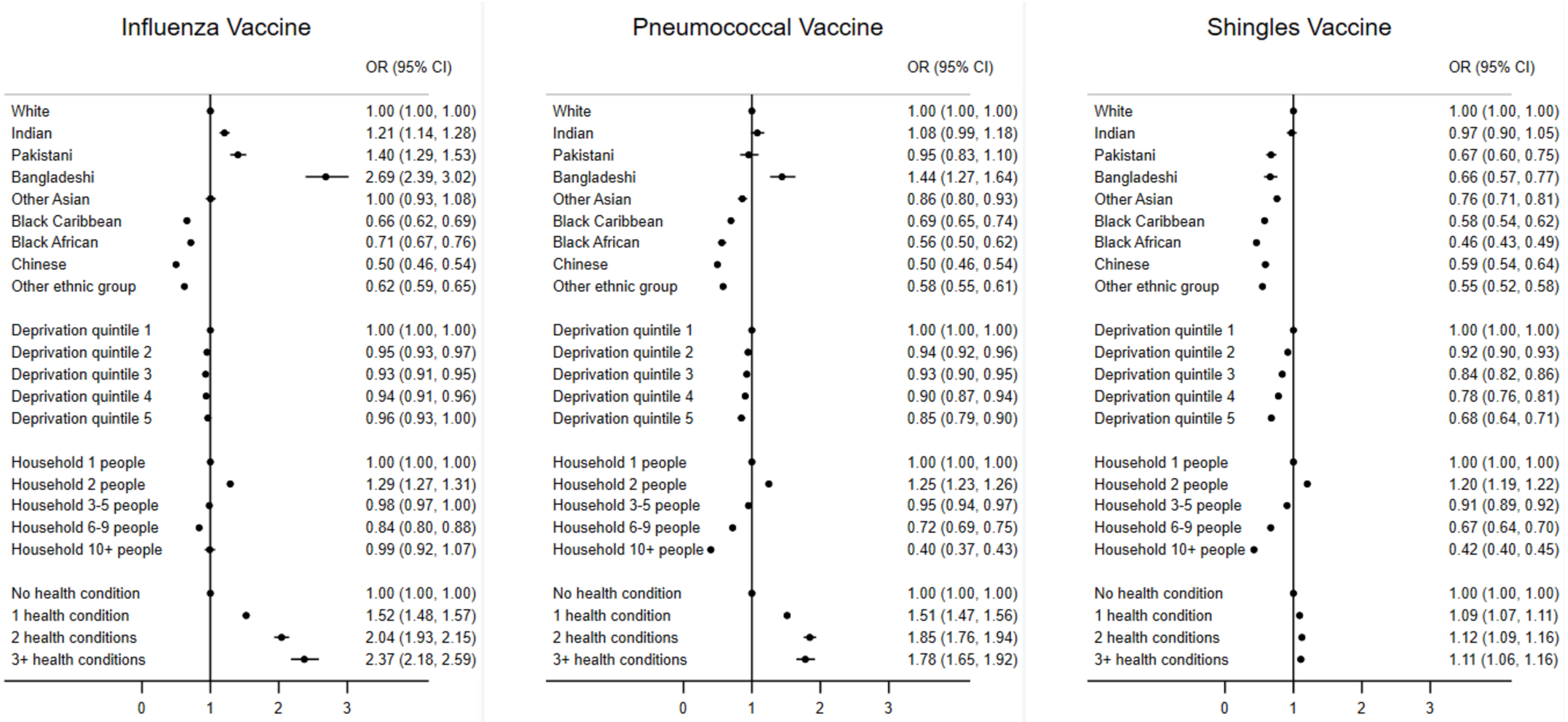

Figure S7: Sensitivity (complete-case analysis): associations of vaccine uptake and ethnic group, deprivation, household size and health conditions.

Vaccination Refusal in Unvaccinated (complete-case analysis)

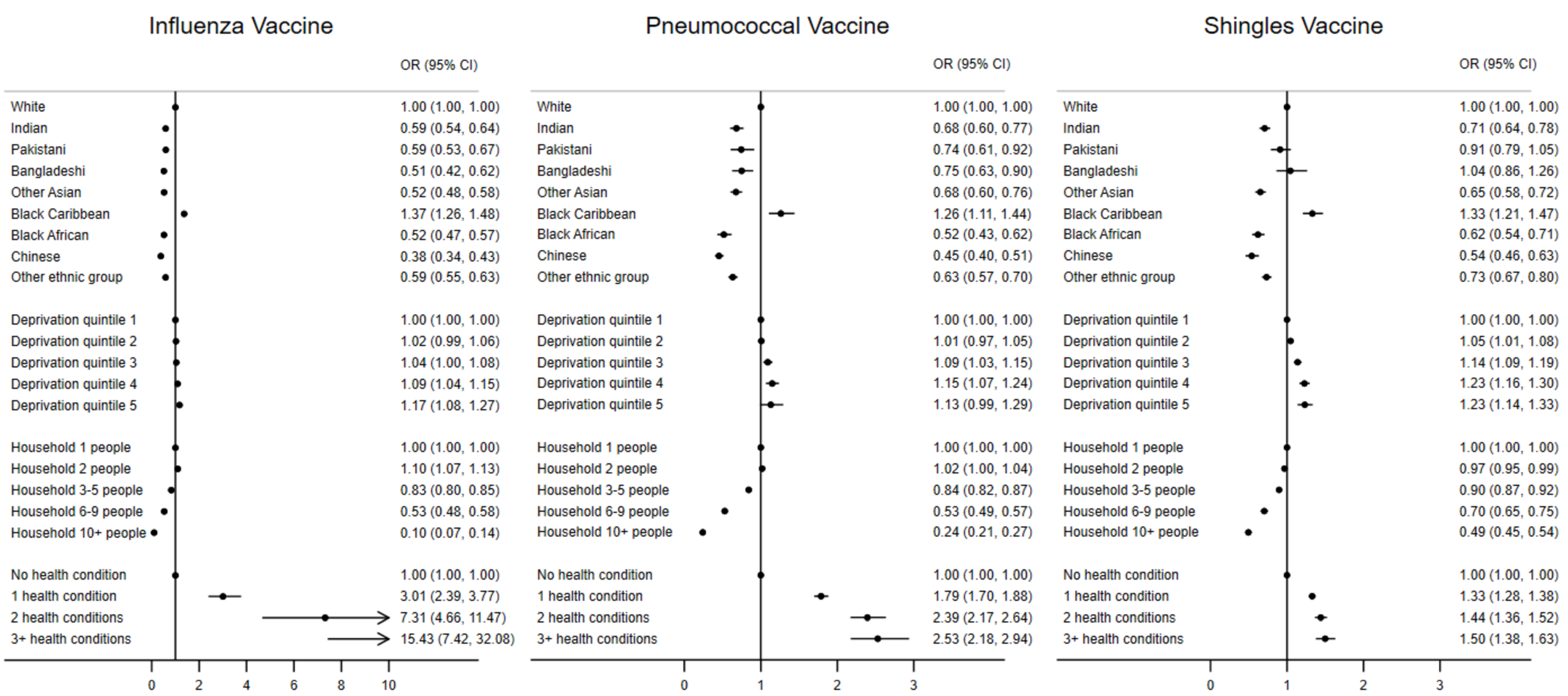

Figure S8: Sensitivity (complete-case analysis): associations of vaccine refusal (in non-vaccinated) and ethnic group, deprivation, household size and health conditions.
