# Supplementary material for: Factors influencing influenza, pneumococcal and shingles vaccine uptake and refusal in older adults: a population-based cross-sectional study in England
Source: BMJ Open. 2023 Mar 15;13(3):e058705. doi: 10.1136/bmjopen-2021-058705 (PMC10030484; doi:10.1136/bmjopen-2021-058705)
Supplement: Author's manuscript [file bmjopen-2021-058705.draft_revisions.pdf]

BMJ Open

BMJ Open is committed to open peer review. As part of this commitment we make the peer review history of every article we publish publicly available.

When an article is published we post the peer reviewers' comments and the authors' responses online. We also post the versions of the paper that were used during peer review. These are the versions that the peer review comments apply to.

The versions of the paper that follow are the versions that were submitted during the peer review process. They are not the versions of record or the final published versions. They should not be cited or distributed as the published version of this manuscript.

BMJ Open is an open access journal and the full, final, typeset and author-corrected version of record of the manuscript is available on our site with no access controls, subscription charges or pay-per-view fees (http://bmjopen.bmj.com).

If you have any questions on BMJ Open's open peer review process please email info.bmjopen@bmj.com

Factors influencing influenza, pneumococcal and shingles vaccine uptake and refusal in older adults: a population-based cross-sectional study in England

Journal:	*BMJ Open*
Manuscript ID	bmjopen-2021-058705
Article Type:	Original research
Date Submitted by the Author:	28-Oct-2021

Complete List of Authors:	Tan, Pui San; University of Oxford, Nuffield Department of Primary Care Health Sciences<br>Patone , Martina ; University of Oxford<br>Clift, Ashley; University of Oxford, Nuffield Department of Primary Care Health Sciences<br>Dambha-Miller, Hajira; University of Southampton Faculty of Medicine, Primary Care Research Centre<br>Saatci, Defne; University of Oxford, Nuffield Department of Primary Care Health Science<br>Ranger, Tom; Oxford University, Primary Care Health Sciences<br>Garriga, Cesar; University of Oxford, Nuffield Department of Primary Care Health Sciences<br>Zaccardi, Francesco; University of Leicester; University of Leicester, Diabetes Research Centre<br>Shah, Baiju; Sunnybrooke Health Sciences Centre<br>Coupland, Carol; University of Nottingham, Division of Primary Care<br>Griffin, Simon; The Primary Care Unit<br>Khunti, Kamlesh; University of Leicester, Diabetes Research Centre<br>Hippisley-Cox, Julia; University of Oxford, Nuffield Department of Primary Care Sciences

Keywords:	EPIDEMIOLOGY, Epidemiology < INFECTIOUS DISEASES, Public health < INFECTIOUS DISEASES, PRIMARY CARE

SCHOLARONE™
Manuscripts

I, the Submitting Author has the right to grant and does grant on behalf of all authors of the Work (as defined in the below author licence), an exclusive licence and/or a non-exclusive licence for contributions from authors who are: i) UK Crown employees; ii) where BMJ has agreed a CC-BY licence shall apply, and/or iii) in accordance with the terms applicable for US Federal Government officers or employees acting as part of their official duties; on a worldwide, perpetual, irrevocable, royalty-free basis to BMJ Publishing Group Ltd ("BMJ") its licensees and where the relevant Journal is co-owned by BMJ to the co-owners of the Journal, to publish the Work in this journal and any other BMJ products and to exploit all rights, as set out in our licence.

The Submitting Author accepts and understands that any supply made under these terms is made by BMJ to the Submitting Author unless you are acting as an employee on behalf of your employer or a postgraduate student of an affiliated institution which is paying any applicable article publishing charge ("APC") for Open Access articles. Where the Submitting Author wishes to make the Work available on an Open Access basis (and intends to pay the relevant APC), the terms of reuse of such Open Access shall be governed by a Creative Commons licence – details of these licences and which Creative Commons licence will apply to this Work are set out in our licence referred to above.

Other than as permitted in any relevant BMJ Author's Self Archiving Policies, I confirm this Work has not been accepted for publication elsewhere, is not being considered for publication elsewhere and does not duplicate material already published. I confirm all authors consent to publication of this Work and authorise the granting of this licence.

For peer review only

Factors influencing influenza, pneumococcal and shingles vaccine uptake and refusal in older adults: a population-based cross-sectional study in England

Pui San Tan[1*], Martina Patone[1*], Ash Kieran Clift[1,2*], Hajira Dambha-Miller[3], Defne Saatci[1], Tom A. Ranger[1], Cesar Garriga[1], Francesco Zaccardi[4], Baiju R. Shah[5], Carol Coupland[6], Simon J. Griffin[7,8], Kamlesh Khunti[4] & Julia Hippisley-Cox[1]

1: Nuffield Department of Primary Care Health Sciences, University of Oxford

2: Cancer Research UK Oxford Centre, Department of Oncology, University of Oxford

3: Primary Care Research Centre, University of Southampton

4: Leicester Diabetes Centre, University of Leicester

5: Department of Medicine, University of Toronto

6: Division of Primary Care, School of Medicine, University of Nottingham

7: Department of Public Health and Primary Care, School of Clinical Medicine, University of Cambridge

8: MRC Epidemiology Unit, School of Clinical Medicine, University of Cambridge

Corresponding author:

Dr Pui San Tan

Nuffield Department of Primary Care Health Sciences, University of Oxford

Radcliffe Primary Care Building, Radcliffe Observatory Quarter, Woodstock Rd,

Oxford OX2 6GG, UK

pui.tan@phc.ox.ac.uk

*Authors contributed equally to this work

For peer review only - http://bmjopen.bmj.com/site/about/guidelines.xhtml

Abstract:

Objectives: Uptake of influenza, pneumococcal and shingles vaccines in older adults remain heterogenous, particularly among the underserved. In this study, we studied the coverage and factors associated with vaccination uptake, as well as refusal in the unvaccinated population and their associations with ethnicity, deprivation, household size, and health conditions.

Design, setting and participants: A cross-sectional study of adults aged 65 years or older in England, using a large primary-care database. Associations of vaccine uptake and refusal in the unvaccinated with ethnicity, deprivation, household size, and health conditions were modelled using multivariable logistic regression.

Outcome measure: Influenza, pneumococcal and shingles vaccine uptake and refusal (in the unvaccinated).

Results: This study included 2,054,463 patients from 1,318 general practices. 1,711,465 (83.3%) received at least one influenza vaccine, 1,391,228 (67.7%) pneumococcal vaccine, and 690,783 (53.4%) shingles vaccine. Compared to White ethnicity, influenza vaccine uptake was lower in Chinese (0.49; 0.45-0.53), 'Other ethnic' groups (0.63; 0.60-0.65), Black Caribbean (odds ratio (OR) 0.68; 95% CI: 0.64-0.71), Black African (0.72; 0.68 -0.77). There was generally lower vaccination uptake among more deprived individuals, people living in larger household sizes (3 or more persons) and those with lesser health conditions. Among those who were unvaccinated, higher odds of refusal was associated with the Black Caribbean ethnic group and marginally with more deprived individuals, but was not associated with those living in household sizes above 3 or more persons or those with lesser health conditions.

Conclusion: Non-vaccine uptake among certain ethnic minority and deprived groups could potentially be driven by refusal. Non-vaccine uptake among individuals from larger households or lesser health conditions were more likely to be driven by other factors than refusal. Understanding these may inform tailored public health messaging to different communities for equitable implementation of vaccination programs.

Keywords: vaccine, uptake, refusal, ethnicity, deprivation, equality

For peer review only - http://bmjopen.bmj.com/site/about/guidelines.xhtml

Strengths and limitations of the study

- Use of a large primary care database offered a population-representative population in terms of demographics including ethnic groups and deprivation

- Using a primary care database captures comprehensive vaccination data, including those occurring outside general practice (such as in pharmacies), as well as recorded invitations to vaccination sent by general practices and patient refusals

- There was lack of recording of variables such as personal beliefs, literacy levels, language barriers, access and education status, and hence we were unable to evaluate the impact of these factors on vaccination uptake and refusal

- Classification of vaccination-related endpoints was reliant on individual practitioners; however, we used an appropriately wide range of codes in our endpoint definitions

For peer review only

For peer review only - http://bmjopen.bmj.com/site/about/guidelines.xhtml

Background

Older adults are often more susceptible to infectious diseases circulating in the community, and may develop more severe health outcomes when infected due to lower immune responses associated with aging[1] and comorbidities. National influenza, pneumococcal, and shingles vaccination programs for older adults have been implemented in the UK in various phases.[2-4] Through these national vaccination programmes, 'seasonal' influenza vaccines are offered annually, pneumococcal vaccines are offered as a single dose to adults aged 65 years and above, whilst the shingles vaccine is offered as a single dose to adults aged 70-79 years.[2-4]

The World Health Organisation (WHO) recommends a target of 75% population vaccination coverage.[5] Recent reports from Public Health England (PHE) have reported 81% influenza vaccination coverage and 69% pneumococcal vaccination coverage in adults aged 65 years and above, and 47% to 77% for shingles vaccination coverage in adults aged 71 and 78, respectively.[2-4] However, some evidence suggests that there could be vast heterogeneity in terms of vaccination coverage, potentially varying by geographical region, ethnicity, deprivation, household size, and health conditions.[2-4 6 7]

For the purposes of equitable public health strategy, it is important to understand factors associated with uptake, non-uptake, and refusal of vaccinations. Prior studies have demonstrated differential uptake of existing vaccinations across sociodemographic groups, however, many studies have either studied single vaccinations, not captured the appreciable heterogeneity inherent to sociodemographic groups (such as by using broad ethnic categories), analysed a small set of relevant health conditions, and relied on potentially imprecise or biased self-report measures.[7-9] In addition, although household size is known to increase the risk of transmission for infectious diseases, evidence on the association between household size and vaccination uptake remains limited.[10] A few previous studies have suggested that individuals from larger households were less likely to be vaccinated, although these studies were small and mainly focused on childhood vaccinations.[11 12] Further, it is of interest to understand the pathway events leading to vaccine non-uptake, and to what extent these are driven by patient refusal.

Here, we evaluated factors associated with uptake and refusal of existing national vaccination programs (influenza, pneumococcal and shingles) in older adults (aged 65 years and above) in England and their associations with ethnic group, deprivation, household size, and health conditions.

For peer review only

Methods

Study population and data source

A population-based cross-sectional study using QResearch (version 45). QResearch is an electronic health record database with over 10 million current patients registered with more than 1800 practices in England using the EMIS database system. QResearch has good representation of the general population of England, particularly in terms of different ethnic groups with proportions close to those reported by Office for National Statistics.[13]

We included adults aged 65-99 years on 24th January 2020 currently registered with 1,318 EMIS general practices (GP), which comprised of 2,054,463 out of approximately 13.7 million patients aged 65 and over registered with a GP in England.[14] We assessed the uptake and refusal of influenza, pneumococcal, and shingles vaccines from 1st January 1989 until 31st October 2020 (last database update). As the shingles vaccination was rolled out nationally in England in 2013 for those aged 70 and 79 (age-specific catch-up),[15] we included in our shingles vaccine analysis only those aged 70 and above, excluding those aged 80 and above in year 2013 as they were not eligible at the time. Uptake was defined as the last recorded instance of receiving the vaccines of interest within the study period. This was mostly in GP surgeries (~99%), but also in-hospital or pharmacy administrations. Refusal was analysed in those with no record of vaccination, defined as last recorded instances of explicit refusal (74-82% of recorded code instances), consent not being given (18-26%), or non-attendance to a scheduled vaccination appointment (0.03-0.3%).[16] Outcomes were defined using code dictionaries comprising relevant Read and SNOMED codes as inputted into the EMIS software by healthcare practitioners.

We extracted demographic data including age, sex, self-reported ethnic group, Townsend deprivation index quintile (derived from patient postcode),[17] geographical region within England (n=10, see Table 1), housing status and household size. Ethnicity were grouped into 9 categories – White (White British, White Irish, Other White), Indian, Pakistani, Bangladeshi, Other Asian, Black Caribbean, Black African, Chinese, Other ethnic group (White & Black, White & Asian, Other mixed, Other Black, Other ethnic group). We also extracted data using GP Read and SNOMED codes from primary care records and ICD-10 codes from hospital records (where available) for diagnoses of asthma, chronic obstructive pulmonary disease (COPD), diabetes mellitus (type 1 and 2), hypertension, coronary heart disease (CHD), atrial fibrillation (AF), congenital heart disease, congestive cardiac failure (CCF), chronic neurological diseases (Parkinson's disease, epilepsy, cerebral palsy), learning disability, dementia, and severe mental illness (schizophrenia, severe depression, bipolar affective disorder and psychosis) and

For peer review only

For peer review only - http://bmjopen.bmj.com/site/about/guidelines.xhtml

immune suppression (based on use of immunosuppressant medications). For each vaccination outcome (uptake and refusal), people with health conditions diagnosed prior to the vaccination outcome were defined as exposed, while those diagnosed with health conditions after the outcome were defined as unexposed. The most recently recorded BMI and smoking status were identified for each individual.

Analyses

Descriptive analyses compared the uptake and refusal of the three vaccinations of interest by ethnic group, Townsend deprivation quintiles, household size and individual health conditions. Percentage uptake of each vaccination in individual general practices was plotted to display between-region variations.

Multivariable logistic regression models examined associations between ethnic group, deprivation, household size, health conditions and vaccination uptake and refusal by calculating adjusted odds ratios (OR) and their 95% confidence intervals (CI). Clustered robust standard errors were used to account for clustering of individuals within general practices. Refusals were evaluated in never-receivers of each vaccine (no uptake). Individual models for each exposure (ethnic group, deprivation, household size, health conditions) and outcome (vaccination uptake and refusal for each vaccine) were fitted separately, allowing for adjustment of confounders: age, sex, geographical region, type of home, smoking status and/or BMI as relevant according to directed acyclic graphs (DAGs) - (i) Ethnicity – no adjustments; (ii) Deprivation - adjusted for age, sex, region, ethnicity, household size; (iii) Household size – adjusted for age, sex, region, ethnicity, deprivation, (iv) Health conditions - age, sex, region, ethnicity, deprivation, household size, house type, smoking and BMI. (Figure S5).

Missing data for ethnic group (18.5%), BMI (5.6%), deprivation quintiles (0.3%) and smoking status (1.0%) were multiply imputed using chained equations under the missing at random assumption. Five imputations were generated using a single rich imputation model incorporating all outcomes, exposures and confounder covariates. Models were fitted in each of the 5 imputed datasets with model coefficients and their standard errors pooled in accordance with Rubin's rules.[18] We also performed sensitivity analyses of results using complete-case analysis.

RECORD guidelines were used for reporting.[19] Statistical analyses were performed using STATA v17.0.[20]

For peer review only - http://bmjopen.bmj.com/site/about/guidelines.xhtml

Results

This study included 2,054,463 patients aged 65 years and older registered with 1,318 general practices. Characteristics of the study population are shown in Table 1 and S1. At least one influenza vaccine was received by 1,711,465 (83.3) patients, a pneumococcal vaccine by 1,391,228 (67.7%), and a shingles vaccine by 690,783 (53.4% of over 70s). Ethnicity data was complete for 81.5% of the study population; 74.1% of the study population were White, with other ethnic minority groups comprising Indian (1.7%), Pakistani (0.9%), Bangladeshi (0.4%), Other Asian (0.9%), Black Caribbean (1.1%), Black African (0.8%), Chinese (0.3%), and 'Other ethnic group' (1.2%). Practice-level heterogeneity in the uptake of all three vaccines was observed, with the lowest median uptake in London practices. Influenza uptake was the highest among all three vaccines (~80%), followed by pneumococcal vaccine (~70%) and shingles vaccine (~50%) (Figure 1).

Vaccination uptake

Vaccination uptake differed by ethnicity, deprivation, household size, and health conditions (Figure 1). In multivariable analysis compared to the White population, those from Black Caribbean, Black African, Chinese and Other ethnic groups showed lower uptake for all three vaccines (Figure 2). Influenza vaccination uptake was significantly lower in Black Caribbean (OR 0.68, 95% CI: 0.64-0.71), Black African (OR 0.72; 0.68-0.77), Chinese (OR 0.49; 0.45-0.53) and 'Other ethnic group' (OR 0.63; 0.60-0.65), but there was significantly higher uptake in Indian (OR 1.21; 1.14-1.28), Pakistani (OR 1.39; 1.28-1.52), and Bangladeshi (OR 2.68; 2.38-3.01) ethnic groups compared to the White group.

There was a similar pattern observed for pneumococcal vaccination uptake: Black Caribbean (OR 0.70; 0.66-0.75), Black African (OR 0.56; 0.51-0.62), Chinese (OR 0.49; 0.45-0.53), 'Other ethnic group' (OR 0.58; 0.55-0.61), and also additionally for Other Asian (OR 0.87; 0.80-0.93). Pneumococcal vaccine uptake was significantly higher only in Bangladeshi ethnic group (OR 1.46; 1.29-1.65) compared to the White group. For shingles vaccine uptake, there was significantly lower uptake in all ethnic minority groups except in Indians (OR 0.98; 0.91-1.05).

For all three vaccines, vaccine uptake was generally lower among the more deprived, with the most deprived (lowest quintile) having 6% to 33% lower odds of vaccine uptake (ORs 0.67 to 0.94) compared to the most affluent. People in households with two people had 22% to 32% higher odds of having a

For peer review only - http://bmjopen.bmj.com/site/about/guidelines.xhtml

vaccine compared to one-person households. However, the odds were lower in household sizes above three, with people in households of 10 or more people having 17% to 63% lower odds to have vaccine uptake compared to one-person households. The uptake of each vaccination was also generally associated with increasing number of health conditions; with asthma being associated with higher uptake of all three vaccines, while atrial fibrillation, congestive cardiac failure, dementia, severe mental illness were being associated with lower uptake of all three vaccines. (Figure S1)

Vaccination refusals in the unvaccinated

There were consistently significantly higher odds of vaccine refusal amongst the Black Caribbean group compared to the White group for all three vaccines; influenza (OR 1.45; 1.34-1.56), pneumococcal (OR 1.29; 1.14-1.46) and shingles (OR 1.35; 1.23-1.49). Indian, Pakistani, Bangladeshi, Other Asian, Black African, Chinese, and Other ethnic groups were significantly less likely to refuse all three vaccines compared to White ethnic group, except for Pakistani and Bangladeshi, which showed no significant association with shingles vaccine refusal. (Figure 3)

There was a general trend of refusal with increasing deprivation, particularly with shingles vaccine in the two most deprived quintiles, OR 1.21; 1.15-1.28, and OR 1.23; 1.14-1.33 (4th and 5th deprivation quintiles, respectively). Higher household size was associated with lower odds of refusal of all three vaccines in households of 3+ people and more. (Figure 3)

In individuals with three or more health conditions, the odds of refusal were: influenza vaccine (OR 10.29; 7.38-14.37), pneumococcal vaccine (OR 2.55; 2.24-2.90), shingles vaccine (1.60; 1.48-1.73). Individuals with type 2 diabetes consistently showed higher vaccine refusal for all three vaccines and individuals with COPD was also associated with higher refusal for influenza and pneumococcal vaccines. (Figure S2)

Sensitivity analyses

We performed sensitivity analyses to evaluate associations of vaccine uptake and refusal using complete-case analyses and results were comparable with main multiply imputed analysis. (Figure S4)

For peer review only - http://bmjopen.bmj.com/site/about/guidelines.xhtml

Discussion

Summary

In this study, we observed generally lower uptake of influenza, pneumococcal and shingles vaccinations in particular ethnic minority groups and deprived populations. Black Caribbean, Black African, Chinese and Other ethnic groups consistently showed lower uptake of all three vaccines studied compared to the White ethnic group. In the unvaccinated population, the Black Caribbean ethnic group consistently showed lower vaccine uptake and increased odds of vaccine refusal for all three vaccines. More deprived populations also showed lower vaccine uptake with higher refusals in the unvaccinated. Household sizes above 3 persons were associated with lower vaccine uptake, but were not associated with higher refusal. Further, a lower number of pre-existing health conditions was generally associated with lower odds of vaccine uptake, although this was not reflected in terms of odds of refusal.

Strengths and limitations

Use of the QResearch database offered a population-representative population with accurately coded data, enabling capture of vaccinations occurring outside general practice (such as in pharmacies), as well as recorded invitations to vaccination sent by general practices and patient refusals. This permitted a robust evaluation of not only uptake, but also possible contributory mechanisms leading to uptake behaviours. Limitations include the lack of recording of variables such as religion, personal beliefs and reasons for refusal that predicate vaccine hesitancy in our sample. Further, our dataset also did not capture literacy levels, language barriers, access and education status, and hence were not able to evaluate the impact of these socioeconomic factors on vaccination uptake and refusal. These could be important factors influencing the complex decision-making and behavioural aspects and hence would warrant further qualitative and ethnography studies. Classification of vaccination-related endpoints was reliant on individual practitioners using Read and SNOMED codes on the EMIS software system; however, as GP surgeries are financially incentivised through 'Quality Outcome Framework' payments to record vaccination services and we used an appropriately wide range of codes in our endpoint definitions, the risk of misclassification may be low.

Comparison with existing literature

Our observations that influenza vaccination uptake is inversely correlated with deprivation and varies across ethnic groups build upon results from a recent study of adults between 2011-2016 using the

For peer review only - http://bmjopen.bmj.com/site/about/guidelines.xhtml

CPRD database.[7] This study analysed seasonal influenza vaccination uptake across 5 'seasons' and similarly found that in the over 65s, Black individuals were significantly less likely than White individuals to receive this vaccination. However, our study finds that South Asians may be more likely to have higher uptake of influenza vaccine, which may warrant further qualitative study to examine potential socioeconomic and behavioural factors driving this observation. Our examination of three vaccinations within a larger sample size (over 2 million vs. 611,000), a more granular categorisation of ethnic groups (9 vs. 4) and regions (10 vs. 4), improved handling of missing data, and our analysis of vaccination refusals in the unvaccinated substantially improves our understanding of these complex public health behaviours. Our results showed that although four ethnic minority groups (Black Caribbean, Black African, Chinese and Other ethnic group) had lower uptake of influenza vaccine, only the Black Caribbean group showed increased odds of refusal among the unvaccinated.

We also found lower vaccine uptake in household sizes above 3 persons, although they also showed lower refusals in the unvaccinated population. This suggests that lower vaccine uptake in larger households could be driven by barriers to vaccine uptake other than due to refusal alone. A study in Hong Kong showed that vaccine uptake in the elderly living with younger family members had lower vaccine uptake compared to elderly living alone or living with other elderly household members.[6] This calls for further ethnographic research to explore social and household characteristics including age structure of household members and its potential association with vaccine uptake in the elderly in England.

Lower vaccine uptake in those with fewer health conditions could potentially be attributable to reduced contact with health services in the healthier population and hence, reduced likelihood to receive 'opportunistic' vaccination offers. Despite that, it is worth noting that our study also found that in the unvaccinated population there remains significant refusal in those with type-2 diabetes and COPD. Possibly relevant factors could be resistance to lifestyle and behaviour changes, in which individuals with diabetes and COPD who might be more likely to have unhealthy lifestyles e.g. smoking[21 22] might also be less receptive to health interventions i.e. vaccines. However, this finding needs confirmation in other studies.

For peer review only - http://bmjopen.bmj.com/site/about/guidelines.xhtml

Implications for research and practice

Two key principles in health inequalities are Tudor-Hart's inverse care law,[23] where service provision is inversely proportional to the need for it, and the inverse equity hypothesis, which posits that new healthcare interventions are most likely to be taken up by those in less need and thus exacerbate pre-existing inequality in the short term. Our study may help inform policymakers regarding reducing inequity in the uptake of the studied vaccines, and tailor public health messaging to diverse communities. Elucidating the extent to which ethnic patterns in vaccine refusal are driven by cultural perceptions, institutional mistrust, variation in penetrance of misinformation, and structural barriers e.g. transport, language and occupational barriers in different ethnic groups requires further study in robust surveys and qualitative research. This may inform tailoring of information dissemination strategies and misinformation countermeasures to specific groups and geographical areas. Furthermore, judicious, longitudinal monitoring of the uptake and refusal rates of vaccines in different ethnic and social groups should enable real-time assessment of developing inequalities, which may inform adaptive public health strategies. Data from this may help develop strategies for increasing uptake in these groups including developing information about vaccines in different languages for use by community leaders, faith groups, local health care providers and community champions.[24]

Conclusions

Vaccine non-uptake amongst ethnic minority and deprived groups varies by the extent to which it is driven by refusal, while non-vaccine uptake among individuals from larger households or lesser health conditions appears likely to be driven by other factors than refusal. Understanding these associations may inform tailored public health messaging to different communities for equitable implementation of vaccination programs.

For peer review only

Author contributions

PST, MP and AKC led conceptualisation of the study, data curation, data analysis, interpretation of results, and wrote first draft and subsequent revisions. All other authors participated in conceptualisation of the study, interpretation of results, critical revision of the manuscript and approved the final version.

Declaration of interests

PST reports previous consultation with AstraZeneca and Duke-NUS outside the submitted work. KK is a Member of the Scientific Advisory Group for Emergencies (SAGE), Member of Independent SAGE, Director of the University of Leicester Centre for Black Minority Health and Trustee of the south Asian Health Foundation. JHC is a member of several SAGE committees and chair of the risk stratification subgroup of the NERVTAG. She is unpaid director of QResearch and founder and former medical director of ClinRisk Ltd (outside the submitted work). MP, AKC, HDM, DS, TAR, FZ, BRS, SJG, CC, CG have no interests to declare.

Acknowledgments

Funding – This project was funded by a grant from the Medical Research Council (MRC Grant Ref: MR/V027778/1). JHC has received grants from the National Institute for Health Research Biomedical Research Centre, Oxford, John Fell Oxford University Press Research Fund, Cancer Research UK (grant number C5255/A18085) through the Cancer Research UK Oxford Centre, and the Oxford Wellcome Institutional Strategic Support Fund (204826/Z/16/Z) during the conduct of the study, is an unpaid director of QResearch, a not-for-profit organisation which is a partnership between the University of Oxford and EMIS Health who supply the QResearch database used for this work, and is a founder and shareholder of ClinRisk Ltd and was its medical director until 31 May 2019; ClinRisk produces open and closed source software to implement clinical risk algorithms (outside this work) into clinical computer systems. AKC is funded by a Clinical Research Training Fellowship from Cancer Research UK (grant C2195/A31310). KK is supported by NIHR Applied Collaboration East Midlands and NIHR Leicester Biomedical Research Centre. The University of Cambridge has received salary support in respect of SJG from the NHS in the East of England through the Clinical Academic Reserve. We acknowledge the contribution of EMIS practices who contribute to QResearch and EMIS Health and the Universities of Nottingham and Oxford for expertise in establishing, developing, or supporting

For peer review only - http://bmjopen.bmj.com/site/about/guidelines.xhtml

the QResearch database. This project involves data derived from patient level information collected by the NHS, as part of the care and support of cancer patients. The data are collated, maintained, and quality assured by the National Cancer Registration and Analysis Service, which is part of Public Health England (PHE). Access to the data was facilitated by the PHE Office for Data Release. The Hospital Episode Statistics data used in this analysis are reused by permission from NHS Digital, which retains the copyright in that data. We thank the Office for National Statistics (ONS) for providing the mortality data. NHS Digital, PHE, and the ONS bear no responsibility for the analysis or interpretation of the data. The investigators acknowledge the philanthropic support of the donors to the University of Oxford's COVID-19 Research Response Fund.

Role of the funding source

The funder had no role in the study design, in the collection, analysis, or interpretation of data, in the writing of the report, or in the decision to submit the paper for publication.

Data statement

To guarantee the confidentiality of personal and health information, only the authors have had access to the data during the study in accordance with the relevant license agreements. Access to QResearch data is according to the information on the QResearch website (www.qresearch.org).

Ethics approval

This was part of a larger project which has been independently peer-reviewed and received ethics approval by the QResearch Scientific board (REC 18/EM/0400; project reference OX102).

Patient and public involvement reporting

Two public representatives advised on interest and appropriateness of the research questions, were involved in writing the protocol for the wider study, and input on lay-summaries describing the planned study.

For peer review only - http://bmjopen.bmj.com/site/about/guidelines.xhtml

For peer review only

References

1. Juliet M. Bartleson DR, Anthony J. Covarrubias, David Furman, Daniel A. Winer & Eric Verdin SARS-CoV-2, COVID-19 and the aging immune system. *Nat Aging* 2021;1:769–82.
2. England PH. Pneumococcal Polysaccharide Vaccine (PPV) coverage report, England, April 2019 to March 2020 2020 [Available from: https://assets.publishing.service.gov.uk/government/uploads/system/uploads/attachment_data/file/899458/hpr1320_ppv-vc.pdf
3. England PH. Seasonal influenza vaccine uptake in GP patients: winter season 2020 to 2021: Public Health England; 2021 [Available from: https://assets.publishing.service.gov.uk/government/uploads/system/uploads/attachment_data/file/996033/Annual-Report_SeasonalFlu-Vaccine_GPs_2020_to_2021.pdf
4. England PH. Shingles vaccine coverage report (adults eligible from April to September 2020 and vaccinated to end of December 2020): England

Quarter 2 report of financial year 2020 to 2021 2021 [Available from: https://assets.publishing.service.gov.uk/government/uploads/system/uploads/attachment_data/file/955523/SP_hpr0321_SHNGLS-vc_Q2-final-3_refs.pdf
5. Europe W. Evaluation of seasonal influenza vaccination policies and coverage in the WHO European Region 2014 [Available from: https://www.euro.who.int/__data/assets/pdf_file/0003/241644/Evaluation-of-seasonal-influenza-vaccination-policies-and-coverage-in-the-WHO-European-Region.pdf
6. Chan DP, Wong NS, Wong EL, et al. Household characteristics and influenza vaccination uptake in the community-dwelling elderly: a cross-sectional study. *Prev Med Rep* 2015;2:803-8. doi: 10.1016/j.pmedr.2015.09.002 [published Online First: 2015/09/21]
7. Loiacono MM, Mahmud SM, Chit A, et al. Patient and practice level factors associated with seasonal influenza vaccine uptake among at-risk adults in England, 2011 to 2016: An age-stratified retrospective cohort study. *Vaccine X* 2020;4:100054. doi: 10.1016/j.jvacx.2020.100054 [published Online First: 2020/01/13]
8. Coupland C, Harcourt S, Vinogradova Y, et al. Inequalities in uptake of influenza vaccine by deprivation and risk group: time trends analysis. *Vaccine* 2007;25(42):7363-71. doi: 10.1016/j.vaccine.2007.08.032 [published Online First: 2007/09/05]
9. Fisher H, Trotter CL, Audrey S, et al. Inequalities in the uptake of human papillomavirus vaccination: a systematic review and meta-analysis. *Int J Epidemiol* 2013;42(3):896-908. doi: 10.1093/ije/dyt049 [published Online First: 2013/04/25]
10. House T, Keeling MJ. Household structure and infectious disease transmission. *Epidemiol Infect* 2009;137(5):654-61. doi: 10.1017/S0950268808001416 [published Online First: 2008/10/08]
11. Rockliffe L, Waller J, Marlow LA, et al. Role of ethnicity in human papillomavirus vaccination uptake: a cross-sectional study of girls from ethnic minority groups attending London schools. *BMJ Open* 2017;7(2):e014527. doi: 10.1136/bmjopen-2016-014527 [published Online First: 2017/02/23]
12. Sarker AR, Akram R, Ali N, et al. Coverage and factors associated with full immunisation among children aged 12-59 months in Bangladesh: insights from the nationwide cross-sectional demographic and health survey. *BMJ Open* 2019;9(7):e028020. doi: 10.1136/bmjopen-2018-028020 [published Online First: 2019/07/09]
13. GOV.UK. Population of England and Wales [Available from: https://www.ethnicity-facts-figures.service.gov.uk/uk-population-by-ethnicity/national-and-regional-populations/population-of-england-and-wales/latest.

For peer review only - http://bmjopen.bmj.com/site/about/guidelines.xhtml

14. Digital N. Patients registered at a GP practice [Available from: https://digital.nhs.uk/data-and-information/data-tools-and-services/data-services/general-practice-data-hub/patients-registered-at-a-gp-practice
15. GOV.UK. Complete routine immunisation schedule: GOV.UK; [13 Feb 2021]. Available from: https://www.gov.uk/government/publications/the-complete-routine-immunisation-schedule.
16. QResearch Web [cited 2021. Available from: https://qweb.qresearch.org.
17. Townsend P , . DN. The Black report: Penguin 1982.
18. Rubin D. Multiple Imputation for Non-response in Surveys: John Wiley 1987.
19. Benchimol EI, Smeeth L, Guttmann A, et al. The REporting of studies Conducted using Observational Routinely-collected health Data (RECORD) statement. *PLoS Med* 2015;12(10):e1001885. doi: 10.1371/journal.pmed.1001885 [published Online First: 2015/10/06]
20. *Stata Statistical Software: Release 17* [program]. College Station, TX: StataCorp LLC.: StataCorp. .
21. Bhatt SP, Kim YI, Harrington KF, et al. Smoking duration alone provides stronger risk estimates of chronic obstructive pulmonary disease than pack-years. *Thorax* 2018;73(5):414-21. doi: 10.1136/thoraxjnl-2017-210722 [published Online First: 20180111]
22. Śliwińska-Mossoń M, Milnerowicz H. The impact of smoking on the development of diabetes and its complications. *Diab Vasc Dis Res* 2017;14(4):265-76. doi: 10.1177/1479164117701876 [published Online First: 20170408]
23. Victora CG, Joseph G, Silva ICM, et al. The Inverse Equity Hypothesis: Analyses of Institutional Deliveries in 286 National Surveys. *Am J Public Health* 2018;108(4):464-71. doi: 10.2105/AJPH.2017.304277 [published Online First: 2018/02/22]
24. Hanif W, Ali SN, Patel K, et al. Cultural competence in covid-19 vaccine rollout. *BMJ* 2020(371):m4845. doi: doi.org/10.1136/bmj.m4845

For peer review only

For peer review only - http://bmjopen.bmj.com/site/about/guidelines.xhtml

Table 1: Characteristics of study population in patients aged 65+ (70+ for shingles)

Characteristics		Study population	Vaccine uptake		
		Overall	Influenza	Pneumococcal	Shingles[a]
Total	N (row %)	2054463	1711465 (83.3)	1391228 (67.7)	690783 (53.4)
Age	Mean (SD)	75.5 (7.7)	76.3 (7.7)	77.1 (7.5)	77.2 (4.4)
	65-69	541272 (26.3)	373566 (21.8)	232831 (16.7)	-
	70-79	922198 (44.9)	793150 (46.3)	665037 (47.8)	469684 (68.0)
	80-89	471167 (22.9)	434074 (25.4)	395456 (28.4)	221099 (32.0)
	90-99	119826 (5.8)	110675 (6.5)	97904 (7.0)	-
Sex	Female	1100957 (53.6)	926592 (54.1)	749022 (53.8)	365203 (52.9)
	Male	953506 (46.4)	784873 (45.9)	642206 (46.2)	325580 (47.1)
Ethnicity	White	1522868 (74.1)	1293856 (75.6)	1064331 (76.5)	539237 (78.1)
	Indian	35618 (1.7)	31062 (1.8)	25454 (1.8)	11293 (1.6)
	Pakistani	17555 (0.9)	15588 (0.9)	12090 (0.9)	4388 (0.6)
	Bangladeshi	8138 (0.4)	7635 (0.4)	6264 (0.5)	2076 (0.3)
	Other Asian	17848 (0.9)	15171 (0.9)	11890 (0.9)	5135 (0.7)
	Black Caribbean	22859 (1.1)	18010 (1.1)	14102 (1.0)	5791 (0.8)
	Black African	16880 (0.8)	13530 (0.8)	9545 (0.7)	3518 (0.5)
	Chinese	6553 (0.3)	4835 (0.3)	3507 (0.3)	1502 (0.2)
	Other ethnic groups	25410 (1.2)	19778 (1.2)	14569 (1.0)	5832 (0.8)
	Ethnicity not recorded	380734 (18.5)	292000 (17.1)	229476 (16.5)	112011 (16.2)
Region	East Midlands	46002 (2.2)	38777 (2.3)	30526 (2.2)	16779 (2.4)
	East of England	93217 (4.5)	77645 (4.5)	64843 (4.7)	34167 (4.9)
	London	322941 (15.7)	261176 (15.3)	204112 (14.7)	92174 (13.3)
	North East	47496 (2.3)	40081 (2.3)	33271 (2.4)	15848 (2.3)
	North West	417970 (20.3)	354779 (20.7)	292600 (21.0)	140099 (20.3)
	South Central	283054 (13.8)	239109 (14.0)	199347 (14.3)	102632 (14.9)
	South East	268594 (13.1)	220952 (12.9)	179031 (12.9)	91516 (13.2)
	South West	256384 (12.5)	213037 (12.4)	169824 (12.2)	87179 (12.6)
	West Midlands	237881 (11.6)	197414 (11.5)	161606 (11.6)	81942 (11.9)
	Yorkshire & Humber	80924 (3.9)	68495 (4.0)	56068 (4.0)	28447 (4.1)
Deprivation quintile	1 (most affluent)	674004 (32.8)	569701 (33.3)	471575 (33.9)	251660 (36.4)
	2	547862 (26.7)	456956 (26.7)	373336 (26.8)	191172 (27.7)
	3	385476 (18.8)	318962 (18.6)	258842 (18.6)	123090 (17.8)
	4	267458 (13.0)	219941 (12.9)	175665 (12.6)	78550 (11.4)
	5 (most deprived)	174280 (8.5)	141551 (8.3)	108526 (7.8)	44651 (6.5)
	Not recorded	5383 (0.3)	4354 (0.3)	3284 (0.2)	1660 (0.2)
Home category	Neither in care home nor homeless	2005725 (97.6)	1665389 (97.3)	1356313 (97.5)	682316 (98.8)
	Care home	47655 (2.3)	45263 (2.6)	34352 (2.5)	8301 (1.2)
	Homeless	1083 (0.1)	813 (0.0)	563 (0.0)	166 (0.0)
Household size	1 person	875588 (42.6)	726447 (42.4)	596361 (42.9)	285715 (41.4)
	2 people	849357 (41.3)	721411 (42.2)	594481 (42.7)	326499 (47.3)

For peer review only - http://bmjopen.bmj.com/site/about/guidelines.xhtml

	3-5 people	255089 (12.4)	199611 (11.7)	152373 (11.0)	65031 (9.4)
	6-9 people	30961 (1.5)	24934 (1.5)	18767 (1.3)	6678 (1.0)
	10 or more	43468 (2.1)	39062 (2.3)	29246 (2.1)	6860 (1.0)
Number of health conditions[c]	0	667163 (32.5)	483507 (28.3)	566398 (40.7)	213919 (31.0)
	1	786798 (38.3)	671330 (39.2)	559648 (40.2)	281353 (40.7)
	2	428751 (20.9)	393220 (23.0)	215126 (15.5)	145583 (21.1)
	3+	171751 (8.4)	163408 (9.5)	50056 (3.6)	49928 (7.2)

[a]Percentage calculated using denominator of shingles eligible population, n = 1,294,176. Percentages are column percentages unless otherwise indicated. SD: standard deviation. [c]Counts only based on conditions included in this study.

For peer review only

For peer review only - http://bmjopen.bmj.com/site/about/guidelines.xhtml

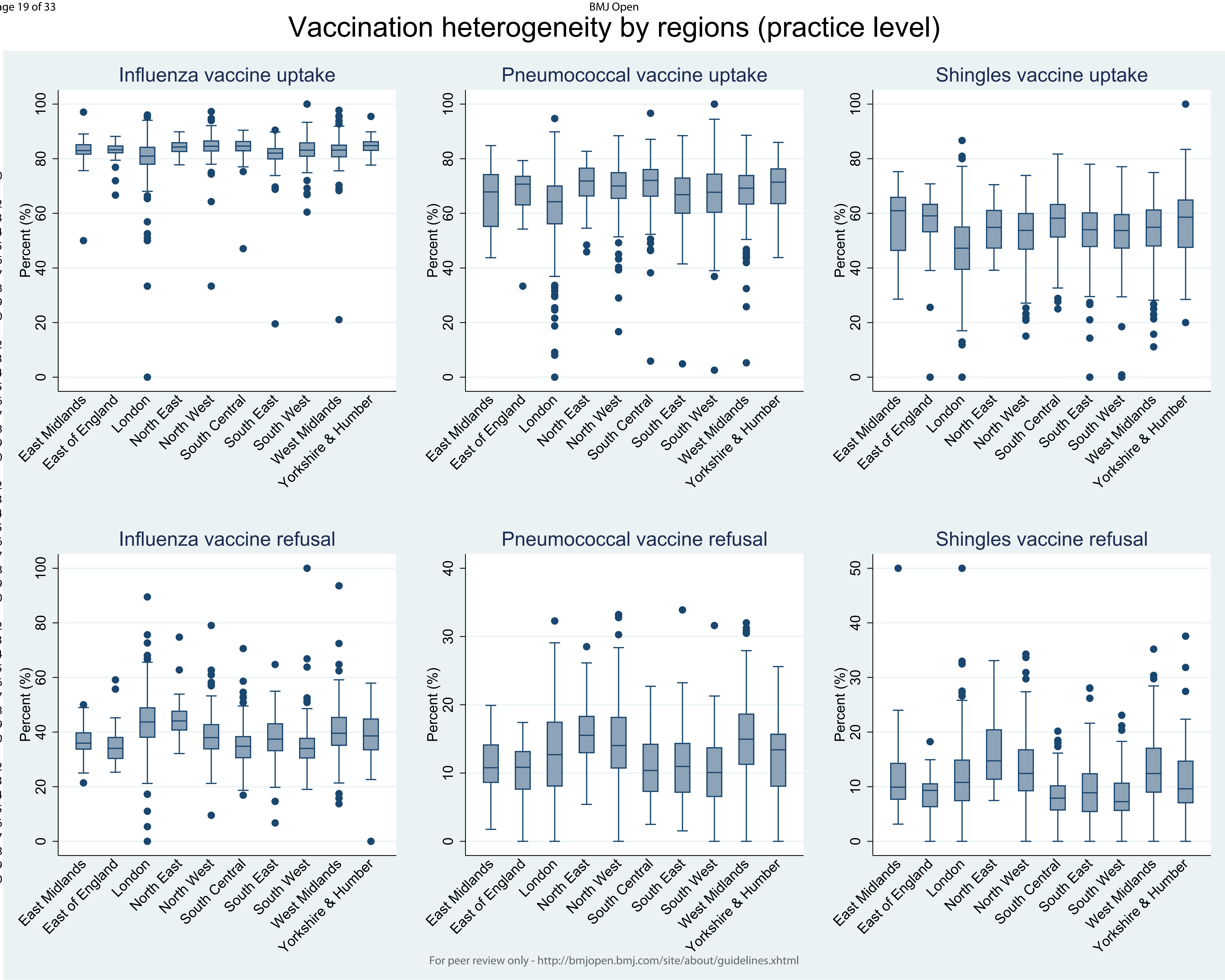
Vaccination heterogeneity by regions (practice level)
Influenza vaccine uptake
Pneumococcal vaccine uptake
Shingles vaccine uptake
Influenza vaccine refusal
Pneumococcal vaccine refusal
Shingles vaccine refusal
Percent (%)
East Midlands
East of England
London
North East
North West
South Central
South East
South West
West Midlands
Yorkshire & Humber

Uptake

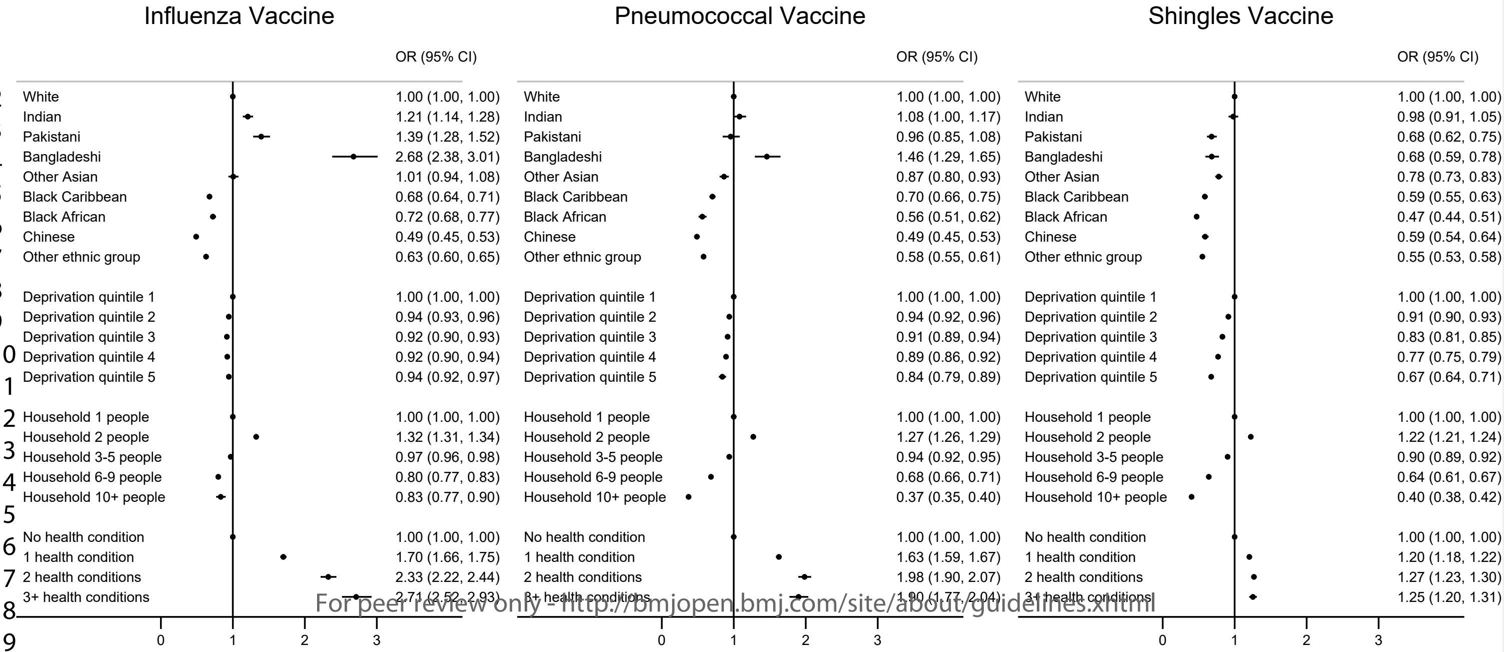

For peer review only - http://bmjopen.bmj.com/site/about/guidelines.xhtml

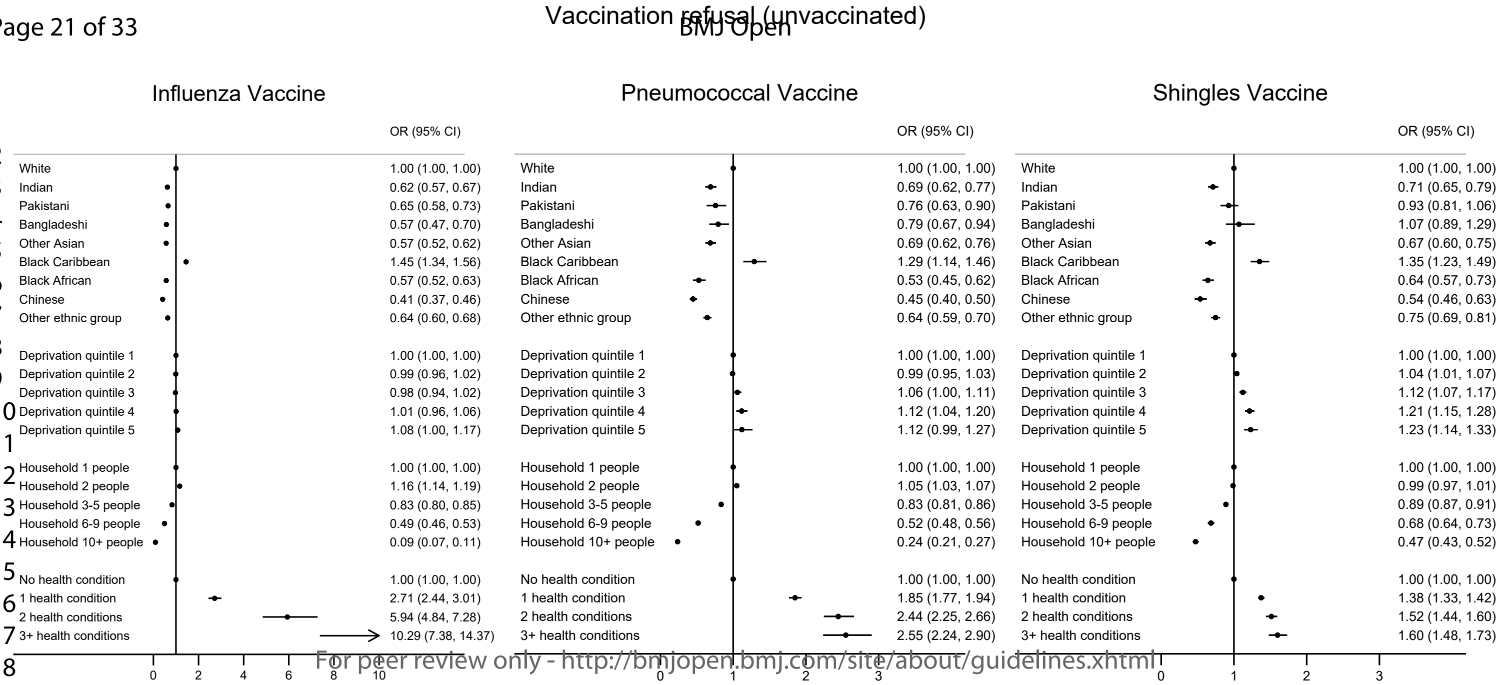

For peer review only - http://bmjopen.bmj.com/site/about/guidelines.xhtml

Supplement

Table S1: Characteristics of study population - lifestyle and health conditions

Characteristics		Study population	Vaccine uptake		
		Overall	Influenza	Pneumococcal	Shingles[a]
Total	N (row %)	2054463	1711465 (83.3)	1391228 (67.7)	690783 (53.4)
Body mass index	<18.5	36406 (1.8)	31088 (1.8)	25321 (1.8)	9351 (1.4)
	18.5-25	615113 (29.9)	515261 (30.1)	421175 (30.3)	204730 (29.6)
	25-30	754859 (36.7)	641998 (37.5)	528282 (38.0)	273645 (39.6)
	30-35	361993 (17.6)	310919 (18.2)	254754 (18.3)	128653 (18.6)
	35-40	121452 (5.9)	105199 (6.1)	85649 (6.2)	41349 (6.0)
	>=40	48792 (2.4)	42948 (2.5)	34151 (2.5)	15061 (2.2)
	Not recorded	115848 (5.6)	64052 (3.7)	41896 (3.0)	17994 (2.6)
Smoking	Non-smoker	1143669 (55.7)	955785 (55.8)	773504 (55.6)	383407 (55.5)
	Ex-smoker	712384 (34.7)	618783 (36.2)	516754 (37.1)	265778 (38.5)
	Current smoker	177685 (8.6)	132076 (7.7)	98773 (7.1)	40903 (5.9)
	Not recorded	20725 (1.0)	4821 (0.3)	2197 (0.2)	695 (0.1)
Health conditions	Asthma	254110 (12.4)	235822 (13.8)	162658 (11.7)	89598 (13.0)
	Chronic obstructive pulmonary disease	160907 (7.8)	150873 (8.8)	66827 (4.8)	52655 (7.6)
	Type-1 diabetes	6253 (0.3)	5908 (0.3)	4243 (0.3)	1882 (0.3)
	Type-2 diabetes	353860 (17.2)	327748 (19.2)	183136 (13.2)	120912 (17.5)
	Hypertension	1013241 (49.3)	901041 (52.6)	559319 (40.2)	360378 (52.2)
	Dementia	86868 (4.2)	81151 (4.7)	8622 (0.6)	10989 (1.6)
	Parkinson's disease	20720 (1.0)	18825 (1.1)	4635 (0.3)	5467 (0.8)
	Epilepsy	38404 (1.9)	33738 (2.0)	19335 (1.4)	10874 (1.6)
	Cerebral palsy	1041 (0.1)	929 (0.1)	598 (0.0)	233 (0.0)
	Learning disability	39959 (1.9)	36644 (2.1)	9192 (0.7)	9897 (1.4)
	Severe mental illness	243791 (11.9)	210885 (12.3)	133322 (9.6)	73294 (10.6)
	Coronary heart disease	294490 (14.3)	273488 (16.0)	153850 (11.1)	101948 (14.8)
	Atrial fibrillation	196503 (9.6)	180461 (10.5)	53438 (3.8)	55647 (8.1)
	Congestive cardiac failure	85674 (4.2)	79600 (4.7)	19891 (1.4)	20144 (2.9)
	Congenital heart disease	14739 (0.7)	13500 (0.8)	6590 (0.5)	4938 (0.7)
	Immunosuppression	17339 (0.8)	16188 (0.9)	8622 (0.6)	3445 (0.5)

[a]Percentage calculated using denominator of shingles eligible population, n = 1,294,176. [b] Comorbidities diagnosed prior to vaccinations in those vaccinated. Percentages are column percentages unless otherwise indicated. SD: standard deviation.

For peer review only

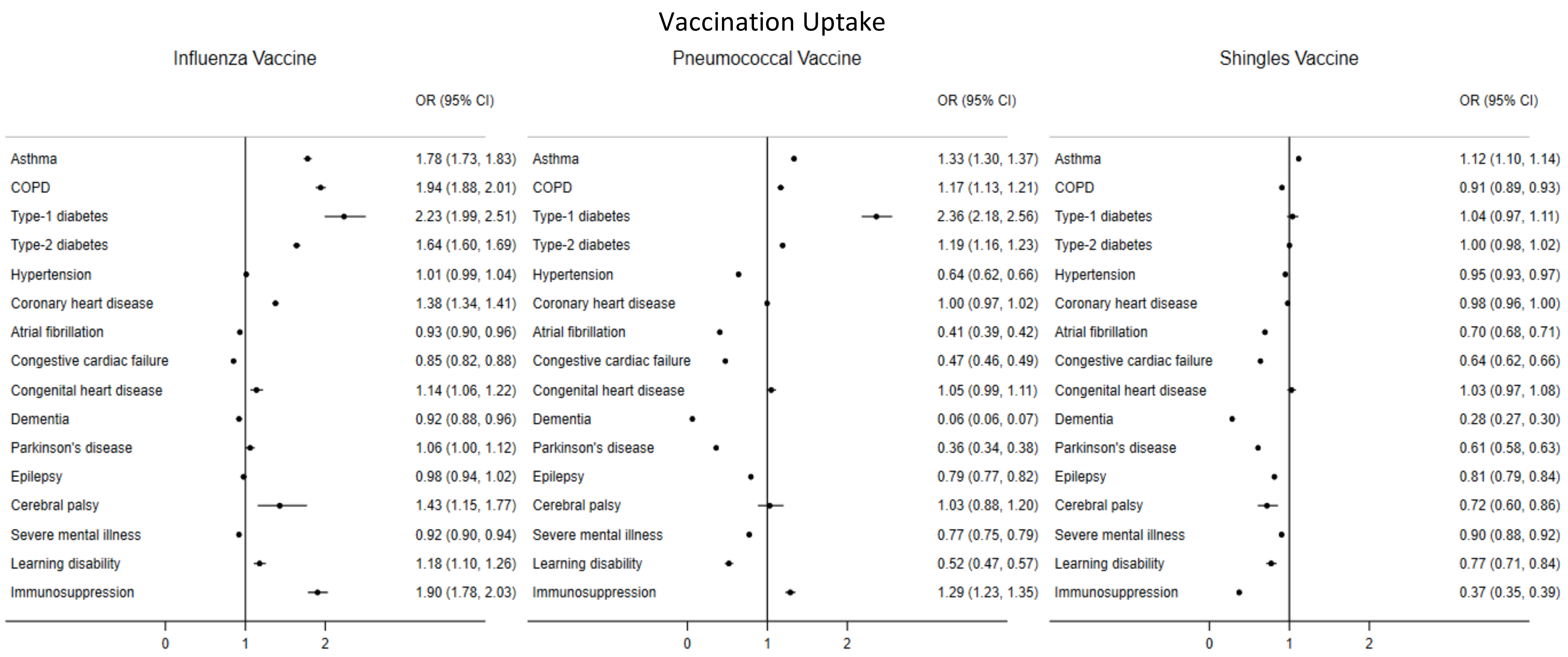

Figure S1: Associations of vaccine uptake and specific health conditions.

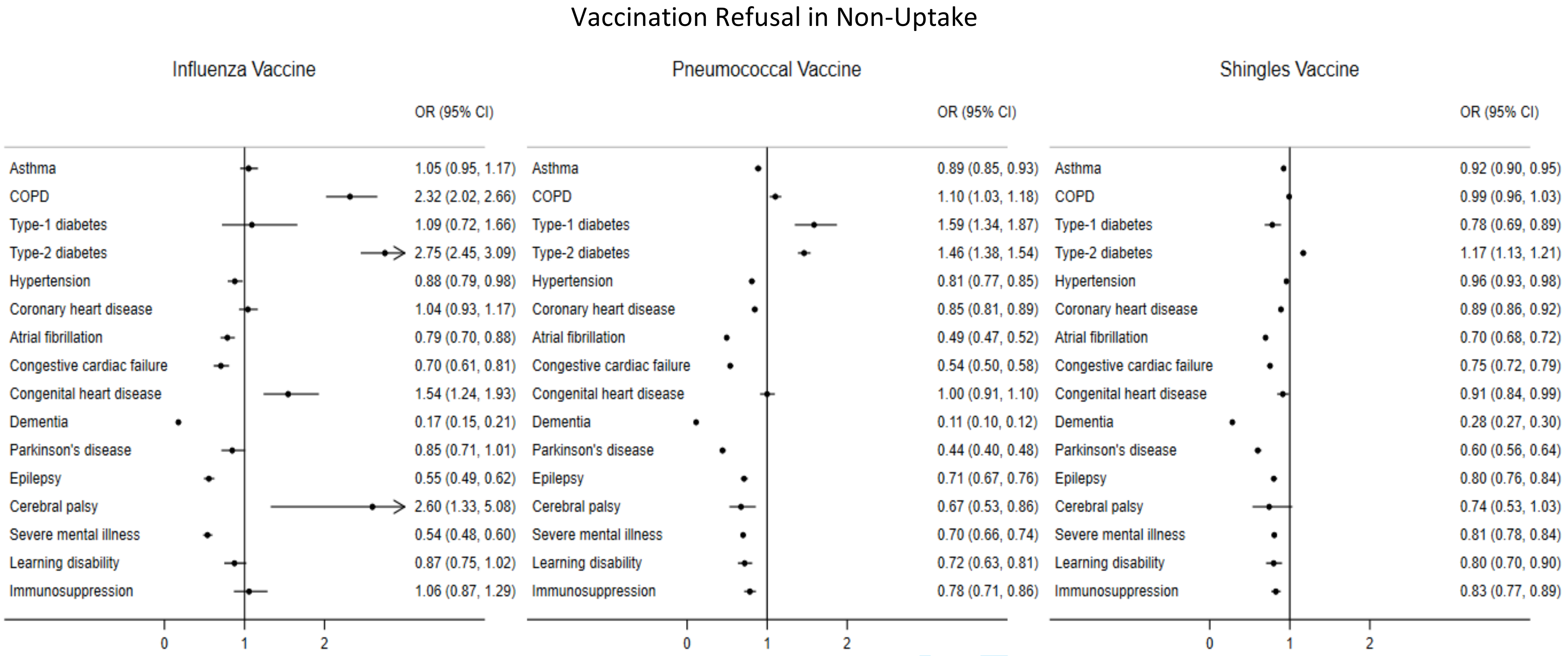

Figure S2: Associations of vaccine refusal in unvaccinated and specific health conditions.

For peer review only - http://bmjopen.bmj.com/site/about/guidelines.xhtml

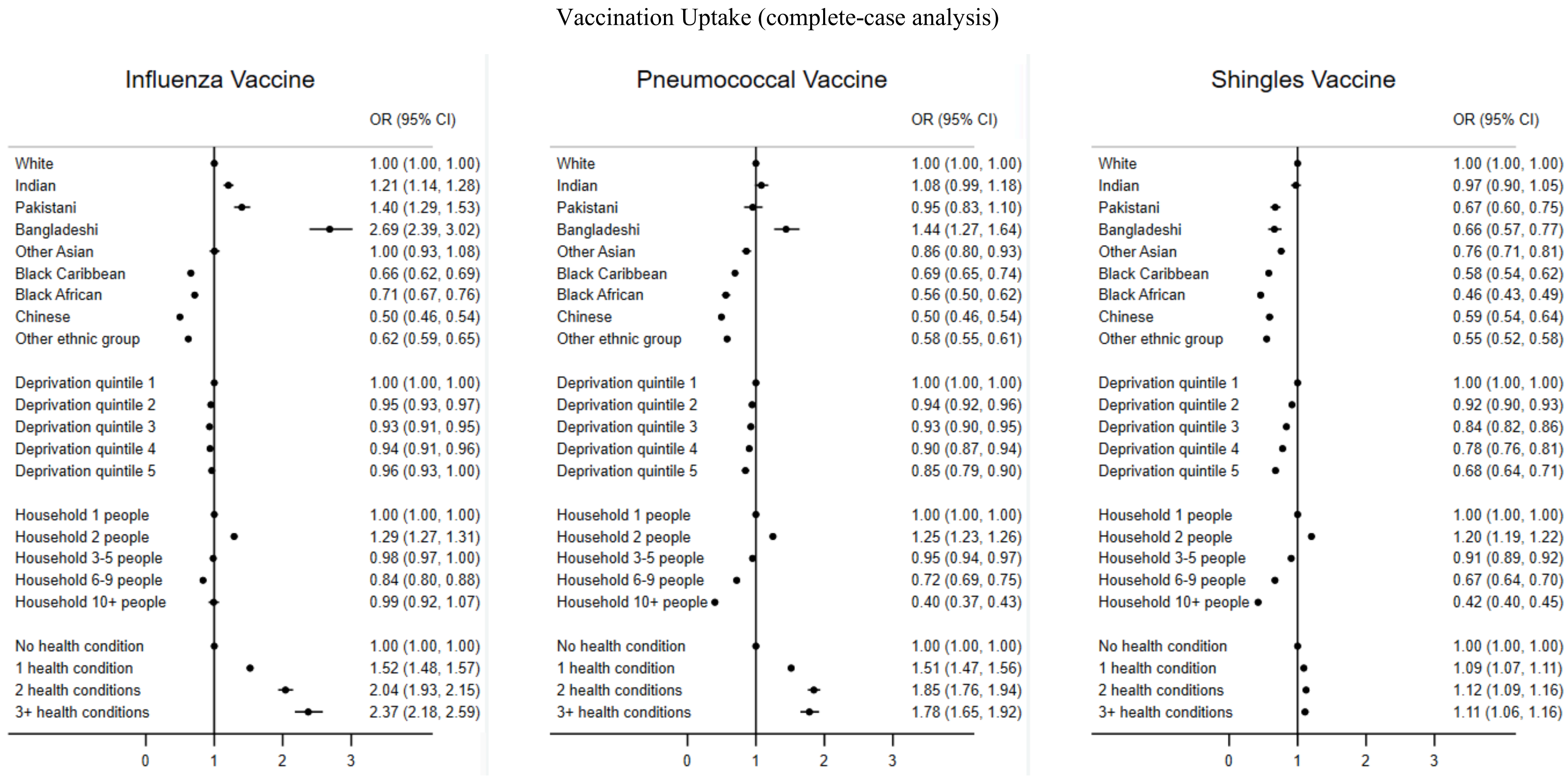

Figure S3: Sensitivity (complete-case analysis): associations of vaccine uptake and ethnic group, deprivation, household size and health conditions.

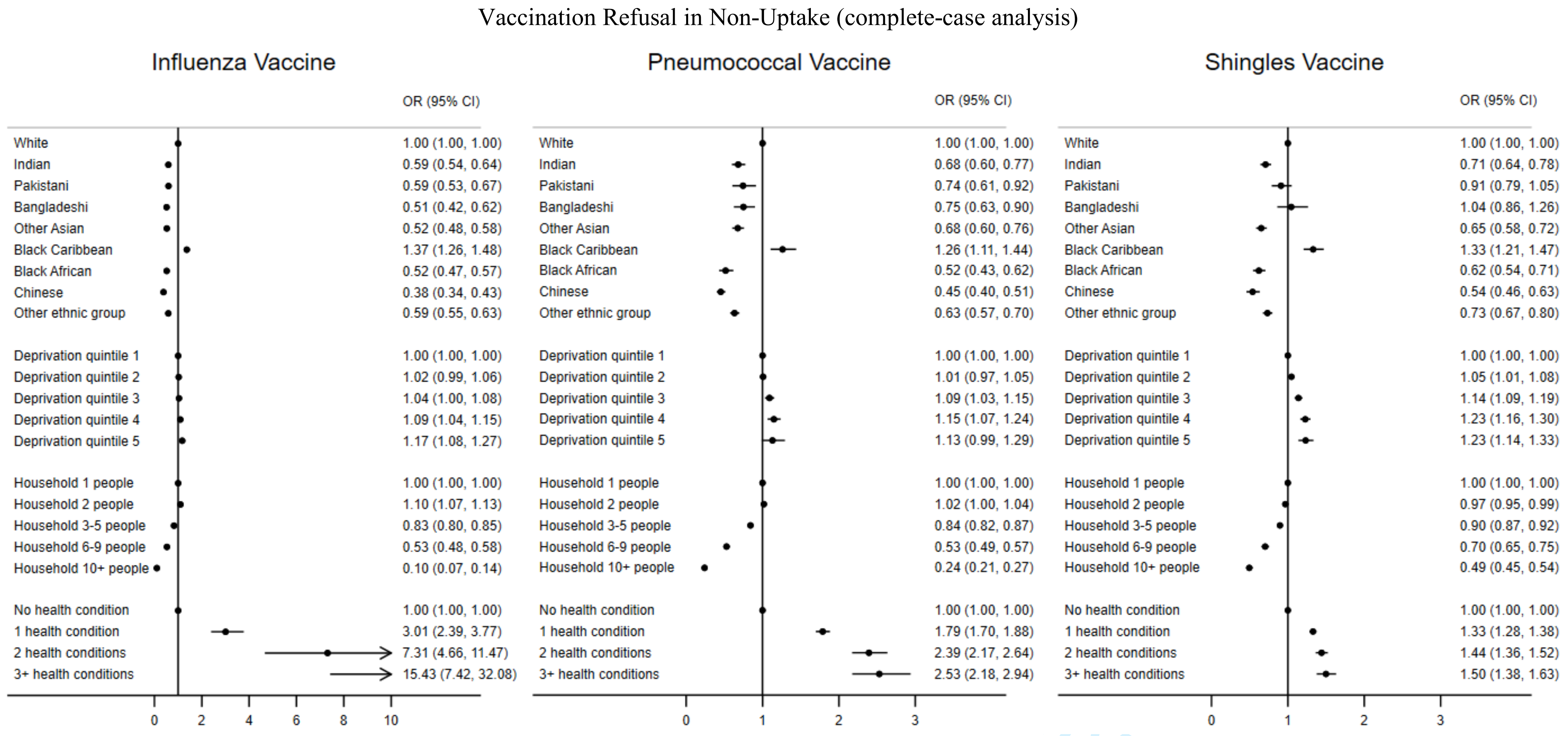

Figure S4: Sensitivity (complete-case analysis): associations of vaccine refusal (in non-vaccinated) and ethnic group, deprivation, household size and health conditions.

Figure S5: Directed acyclic graphs (DAGs) modelling exposures and corresponding outcomes. Green circles denote exposure and blue circle with "I" denote outcome. Other blue circles denote covariates for adjustment while white circles denote variable not for adjustment in each model.

Exposure: Ethnicity
Outcome: Vaccination
Confounder adjustment: None

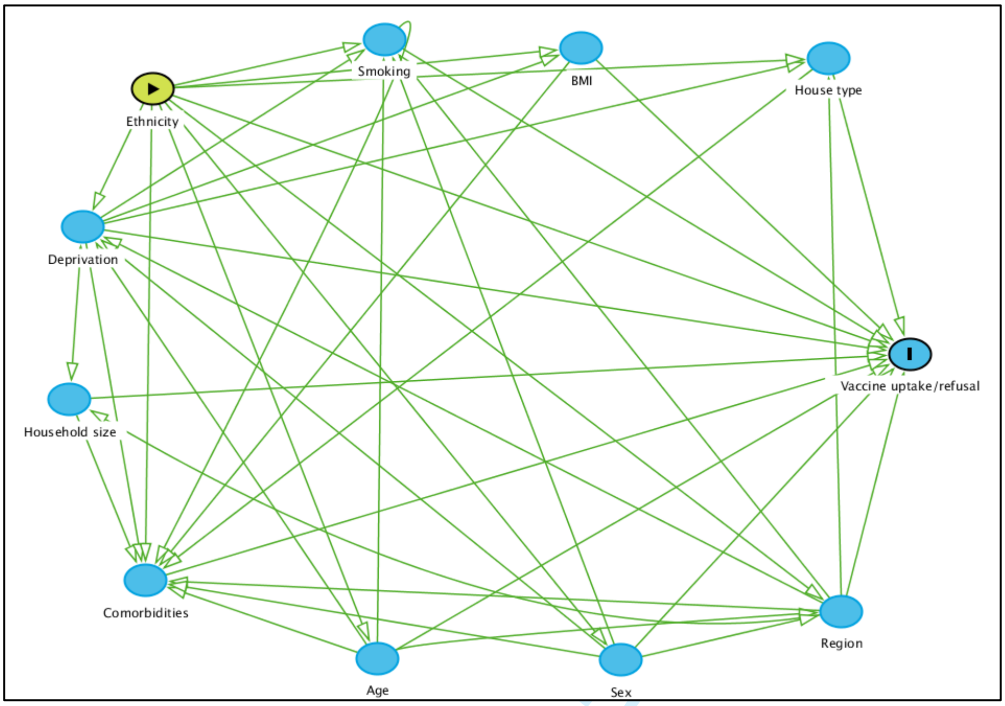

Exposure: Deprivation
Outcome: Vaccination
Confounder adjustment: age, sex, region, ethnicity, household size

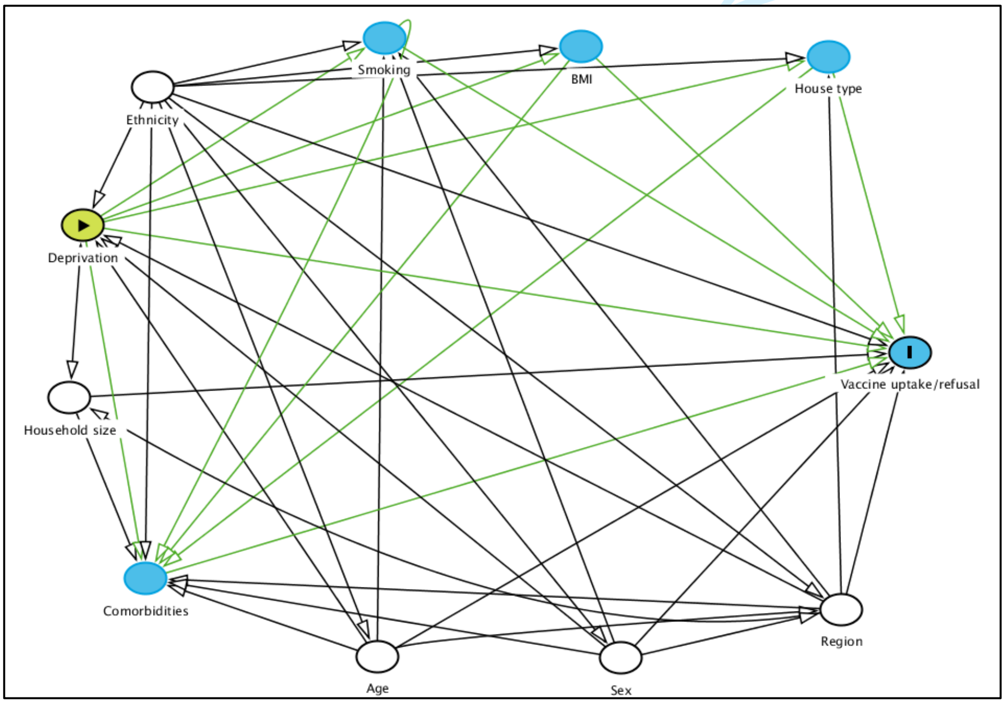

Exposure: Household size
Outcome: Vaccination
Confounder adjustment: age, sex, region, ethnicity, deprivation

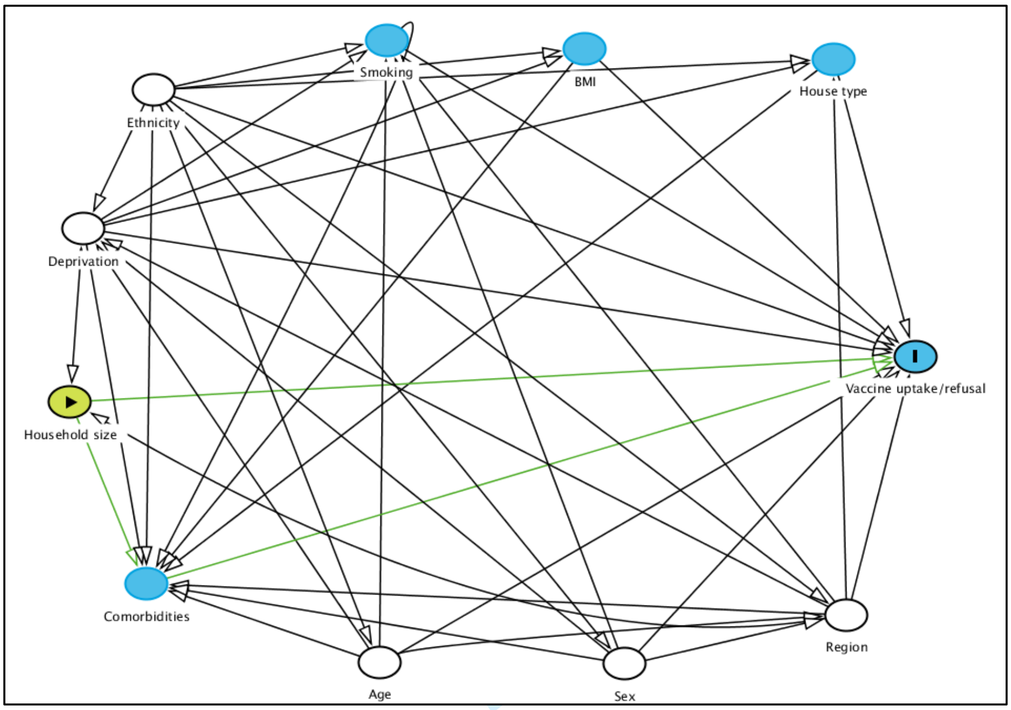

Exposure: Comorbidities
Outcome: Vaccination
Confounder adjustment: age, sex, region, ethnicity, deprivation, household size, house type, smoking, BMI

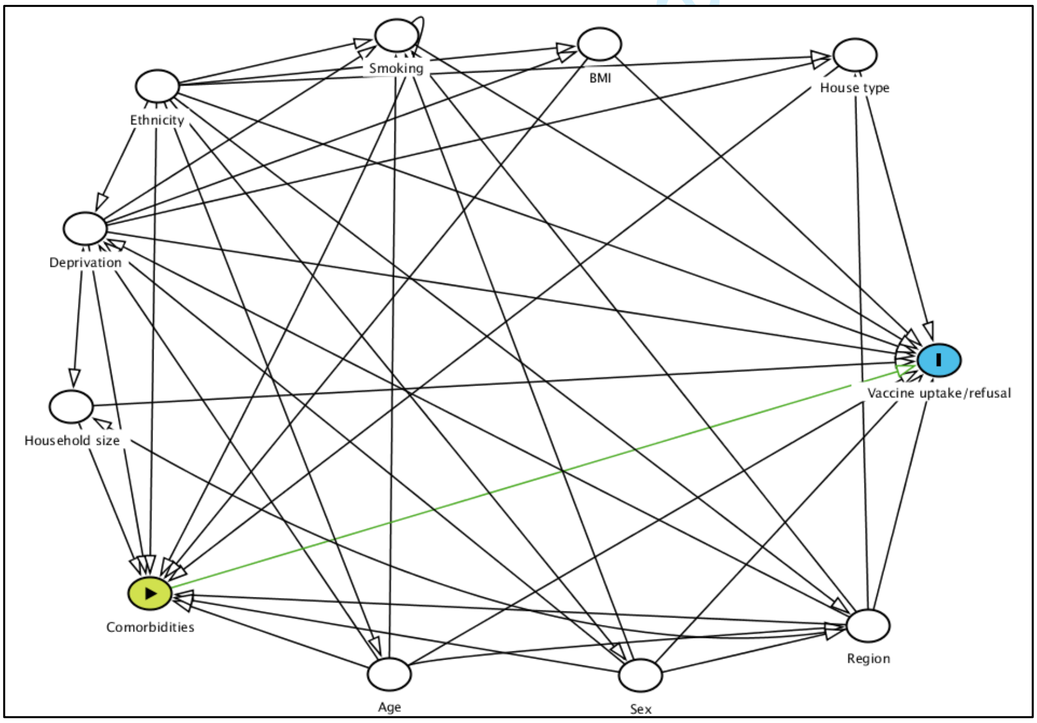

The RECORD statement – checklist of items, extended from the STROBE statement, that should be reported in observational studies using routinely collected health data.[1]

	Item No.	STROBE items	Location in manuscript where items are reported	RECORD items	Location in manuscript where items are reported
**Title and abstract**					
	1	(a) Indicate the study's design with a commonly used term in the title or the abstract (b) Provide in the abstract an informative and balanced summary of what was done and what was found	Pages 1-2	RECORD 1.1: The type of data used should be specified in the title or abstract. When possible, the name of the databases used should be included.<br><br>RECORD 1.2: If applicable, the geographic region and timeframe within which the study took place should be reported in the title or abstract.<br><br>RECORD 1.3: If linkage between databases was conducted for the study, this should be clearly stated in the title or abstract.	Page 1-2
**Introduction**					
Background rationale	2	Explain the scientific background and rationale for the investigation being reported	Page 4		
Objectives	3	State specific objectives, including any prespecified hypotheses	Page 4		
**Methods**					
Study Design	4	Present key elements of study design early in the paper	Page 5		
Setting	5	Describe the setting, locations, and relevant dates, including periods of recruitment, exposure, follow-up, and data collection	Page 5		

For peer review only

Participants	6	*(a) Cohort study* - Give the eligibility criteria, and the sources and methods of selection of participants. Describe methods of follow-up<br>*Case-control study* - Give the eligibility criteria, and the sources and methods of case ascertainment and control selection. Give the rationale for the choice of cases and controls<br>*Cross-sectional study* - Give the eligibility criteria, and the sources and methods of selection of participants<br><br>*(b) Cohort study* - For matched studies, give matching criteria and number of exposed and unexposed<br>*Case-control study* - For matched studies, give matching criteria and the number of controls per case	Page 5	RECORD 6.1: The methods of study population selection (such as codes or algorithms used to identify subjects) should be listed in detail. If this is not possible, an explanation should be provided.<br><br>RECORD 6.2: Any validation studies of the codes or algorithms used to select the population should be referenced. If validation was conducted for this study and not published elsewhere, detailed methods and results should be provided.<br><br>RECORD 6.3: If the study involved linkage of databases, consider use of a flow diagram or other graphical display to demonstrate the data linkage process, including the number of individuals with linked data at each stage.	Page 5
Variables	7	Clearly define all outcomes, exposures, predictors, potential confounders, and effect modifiers. Give diagnostic criteria, if applicable.	Pages 5-6	RECORD 7.1: A complete list of codes and algorithms used to classify exposures, outcomes, confounders, and effect modifiers should be provided. If these cannot be reported, an explanation should be provided.	Page 5-6
Data sources/ measurement	8	For each variable of interest, give sources of data and details of methods of assessment (measurement).<br>Describe comparability of assessment methods if there is more than one group	Pages 5-6		

For peer review only

Bias	9	Describe any efforts to address potential sources of bias	Page 6		
Study size	10	Explain how the study size was arrived at	Pages 5-6		
Quantitative variables	11	Explain how quantitative variables were handled in the analyses. If applicable, describe which groupings were chosen, and why	Pages 5-6		
Statistical methods	12	(a) Describe all statistical methods, including those used to control for confounding<br>(b) Describe any methods used to examine subgroups and interactions<br>(c) Explain how missing data were addressed<br>(d) *Cohort study* - If applicable, explain how loss to follow-up was addressed<br>*Case-control study* - If applicable, explain how matching of cases and controls was addressed<br>*Cross-sectional study* - If applicable, describe analytical methods taking account of sampling strategy<br>(e) Describe any sensitivity analyses	Page 6		
Data access and cleaning methods		..		RECORD 12.1: Authors should describe the extent to which the investigators had access to the database population used to create the study population.<br><br>RECORD 12.2: Authors should provide information on the data	Pages 5-6

For peer review only

				cleaning methods used in the study.	
Linkage		..		RECORD 12.3: State whether the study included person-level, institutional-level, or other data linkage across two or more databases. The methods of linkage and methods of linkage quality evaluation should be provided.	-
**Results**					
Participants	13	(a) Report the numbers of individuals at each stage of the study (*e.g.*, numbers potentially eligible, examined for eligibility, confirmed eligible, included in the study, completing follow-up, and analysed)<br>(b) Give reasons for non-participation at each stage.<br>(c) Consider use of a flow diagram	Page 7	RECORD 13.1: Describe in detail the selection of the persons included in the study (*i.e.,* study population selection) including filtering based on data quality, data availability and linkage. The selection of included persons can be described in the text and/or by means of the study flow diagram.	Page 7
Descriptive data	14	(a) Give characteristics of study participants (*e.g.*, demographic, clinical, social) and information on exposures and potential confounders<br>(b) Indicate the number of participants with missing data for each variable of interest<br>(c) *Cohort study* - summarise follow-up time (*e.g.*, average and total amount)	Page 7		
Outcome data	15	*Cohort study* - Report numbers of outcome events or summary measures over time<br>*Case-control study* - Report numbers in each exposure	Page 7		

For peer review only

		category, or summary measures of exposure *Cross-sectional study* - Report numbers of outcome events or summary measures			
Main results	16	(a) Give unadjusted estimates and, if applicable, confounder-adjusted estimates and their precision (e.g., 95% confidence interval). Make clear which confounders were adjusted for and why they were included (b) Report category boundaries when continuous variables were categorized (c) If relevant, consider translating estimates of relative risk into absolute risk for a meaningful time period	Pages 7-8		
Other analyses	17	Report other analyses done—e.g., analyses of subgroups and interactions, and sensitivity analyses	Page 8		
**Discussion**					
Key results	18	Summarise key results with reference to study objectives	Page 9		
Limitations	19	Discuss limitations of the study, taking into account sources of potential bias or imprecision. Discuss both direction and magnitude of any potential bias	Page 9	RECORD 19.1: Discuss the implications of using data that were not created or collected to answer the specific research question(s). Include discussion of misclassification bias, unmeasured confounding, missing data, and changing eligibility over time, as they pertain to the study being reported.	Page 9
Interpretation	20	Give a cautious overall interpretation of results	Pages 9-10		

For peer review only

		considering objectives, limitations, multiplicity of analyses, results from similar studies, and other relevant evidence			
Generalisability	21	Discuss the generalisability (external validity) of the study results	Pages 10-11		
**Other Information**					
Funding	22	Give the source of funding and the role of the funders for the present study and, if applicable, for the original study on which the present article is based	Page 12-13		
Accessibility of protocol, raw data, and programming code		..	Page 13	RECORD 22.1: Authors should provide information on how to access any supplemental information such as the study protocol, raw data, or programming code.	Page 13

*Reference: Benchimol EI, Smeeth L, Guttmann A, Harron K, Moher D, Petersen I, Sørensen HT, von Elm E, Langan SM, the RECORD Working Committee. The REporting of studies Conducted using Observational Routinely-collected health Data (RECORD) Statement. *PLoS Medicine* 2015; in press.

*Checklist is protected under Creative Commons Attribution (CC BY) license.

For peer review only

BMJ Open

Factors influencing influenza, pneumococcal and shingles vaccine uptake and refusal in older adults: a population-based cross-sectional study in England

Journal:	*BMJ Open*
Manuscript ID	bmjopen-2021-058705.R1
Article Type:	Original research
Date Submitted by the Author:	24-May-2022
Complete List of Authors:	Tan, Pui San; University of Oxford, Nuffield Department of Primary Care Health Sciences<br>Patone , Martina ; University of Oxford<br>Clift, Ashley; University of Oxford, Nuffield Department of Primary Care Health Sciences<br>Dambha-Miller, Hajira; University of Southampton Faculty of Medicine, Primary Care Research Centre<br>Saatci, Defne; University of Oxford, Nuffield Department of Primary Care Health Science<br>Ranger, Tom; Oxford University, Primary Care Health Sciences<br>Garriga, Cesar; University of Oxford, Nuffield Department of Primary Care Health Sciences<br>Zaccardi, Francesco; University of Leicester; University of Leicester, Diabetes Research Centre<br>Shah, Baiju; Sunnybrooke Health Sciences Centre<br>Coupland, Carol; University of Nottingham, Division of Primary Care<br>Griffin, Simon; The Primary Care Unit<br>Khunti, Kamlesh; University of Leicester, Diabetes Research Centre<br>Hippisley-Cox, Julia; University of Oxford, Nuffield Department of Primary Care Sciences
**Primary Subject Heading**:	Public health
Secondary Subject Heading:	Epidemiology, Geriatric medicine, Infectious diseases
Keywords:	EPIDEMIOLOGY, Epidemiology < INFECTIOUS DISEASES, Public health < INFECTIOUS DISEASES, PRIMARY CARE

SCHOLARONE™
Manuscripts

BMJ

I, the Submitting Author has the right to grant and does grant on behalf of all authors of the Work (as defined in the below author licence), an exclusive licence and/or a non-exclusive licence for contributions from authors who are: i) UK Crown employees; ii) where BMJ has agreed a CC-BY licence shall apply, and/or iii) in accordance with the terms applicable for US Federal Government officers or employees acting as part of their official duties; on a worldwide, perpetual, irrevocable, royalty-free basis to BMJ Publishing Group Ltd ("BMJ") its licensees and where the relevant Journal is co-owned by BMJ to the co-owners of the Journal, to publish the Work in this journal and any other BMJ products and to exploit all rights, as set out in our licence.

The Submitting Author accepts and understands that any supply made under these terms is made by BMJ to the Submitting Author unless you are acting as an employee on behalf of your employer or a postgraduate student of an affiliated institution which is paying any applicable article publishing charge ("APC") for Open Access articles. Where the Submitting Author wishes to make the Work available on an Open Access basis (and intends to pay the relevant APC), the terms of reuse of such Open Access shall be governed by a Creative Commons licence – details of these licences and which Creative Commons licence will apply to this Work are set out in our licence referred to above.

Other than as permitted in any relevant BMJ Author's Self Archiving Policies, I confirm this Work has not been accepted for publication elsewhere, is not being considered for publication elsewhere and does not duplicate material already published. I confirm all authors consent to publication of this Work and authorise the granting of this licence.

For peer review only

Factors influencing influenza, pneumococcal and shingles vaccine uptake and refusal in older adults: a population-based cross-sectional study in England

Pui San Tan[1*], Martina Patone[1*], Ash Kieran Clift[1,2*], Hajira Dambha-Miller[3], Defne Saatci[1], Tom A. Ranger[1], Cesar Garriga[1], Francesco Zaccardi[4], Baiju R. Shah[5], Carol Coupland[6], Simon J. Griffin[7,8], Kamlesh Khunti[4] & Julia Hippisley-Cox[1]

1: Nuffield Department of Primary Care Health Sciences, University of Oxford

2: Cancer Research UK Oxford Centre, Department of Oncology, University of Oxford

3: Primary Care Research Centre, University of Southampton

4: Leicester Diabetes Centre, University of Leicester

5: Department of Medicine, University of Toronto

6: Division of Primary Care, School of Medicine, University of Nottingham

7: Department of Public Health and Primary Care, School of Clinical Medicine, University of Cambridge

8: MRC Epidemiology Unit, School of Clinical Medicine, University of Cambridge

Corresponding author:

Dr Pui San Tan

Nuffield Department of Primary Care Health Sciences, University of Oxford

Radcliffe Primary Care Building, Radcliffe Observatory Quarter, Woodstock Rd,

Oxford OX2 6GG, UK

pui.tan@phc.ox.ac.uk

*Authors contributed equally to this work

For peer review only - http://bmjopen.bmj.com/site/about/guidelines.xhtml

Abstract:

Objectives: Uptake of influenza, pneumococcal and shingles vaccines in older adults remain varied across different regions and socioeconomic backgrounds. In this study, we studied the coverage and factors associated with vaccination uptake, as well as refusal in the unvaccinated population and their associations with ethnicity, deprivation, household size, and health conditions.

Design, setting and participants: This is a cross-sectional study of adults aged 65 years or older in England, using a large primary-care database. Associations of vaccine uptake and refusal in the unvaccinated with ethnicity, deprivation, household size, and health conditions were modelled using multivariable logistic regression.

Outcome measure: Influenza, pneumococcal and shingles vaccine uptake and refusal (in the unvaccinated).

Results: This study included 2,054,463 patients from 1,318 general practices. 1,711,465 (83.3%) received at least one influenza vaccine, 1,391,228 (67.7%) pneumococcal vaccine, and 690,783 (53.4%) shingles vaccine. Compared to White ethnicity, influenza vaccine uptake was lower in Chinese (odds ratio (OR) 0.49; 95% CI: 0.45-0.53), 'Other ethnic' groups (0.63; 0.60-0.65), Black Caribbean (0.68; 0.64-0.71), and Black African (0.72; 0.68 -0.77). There was generally lower vaccination uptake among more deprived individuals, people living in larger household sizes (3 or more persons) and those with lesser health conditions. Among those who were unvaccinated, higher odds of refusal was associated with the Black Caribbean ethnic group and marginally with more deprived individuals, but was not associated with higher refusal in those living in large households or those with lesser health conditions.

Conclusion: Certain ethnic minority, deprived populations, large households and healthier individuals were less likely to receive a vaccine, although higher refusal was only associated with ethnicity and deprivation but not larger households nor healthier individuals. Understanding these may inform tailored public health messaging to different communities for equitable implementation of vaccination programs.

Keywords: vaccine, uptake, refusal, ethnicity, deprivation, equality

For peer review only - http://bmjopen.bmj.com/site/about/guidelines.xhtml

Strengths and limitations of the study

- Use of a large primary care database offered a population-representative population in terms of demographics including ethnic groups and deprivation

- Using a primary care database captures comprehensive vaccination data, including those occurring outside general practice (such as in pharmacies), as well as recorded invitations to vaccination sent by general practices and patient refusals

- There was lack of recording of variables such as personal beliefs, literacy levels, language barriers, access and education status, and hence we were unable to evaluate the impact of these factors on vaccination uptake and refusal

- Classification of vaccination-related endpoints was reliant on individual practitioners; however, we used an appropriately wide range of codes in our endpoint definitions

For peer review only

For peer review only - http://bmjopen.bmj.com/site/about/guidelines.xhtml

Background

Older adults are often more susceptible to infectious diseases circulating in the community, and may develop more severe health outcomes when infected due to lower immune responses associated with aging[1] and comorbidities. National influenza, pneumococcal, and shingles vaccination programs for older adults have been implemented in the UK in various phases.[2-4] Through these national vaccination programmes, 'seasonal' influenza vaccines are offered annually, pneumococcal vaccines are offered as a single dose to adults aged 65 years and above, whilst the shingles vaccine is offered as a single dose to adults aged 70-79 years.[2-4]

The World Health Organisation (WHO) recommends a target of 75% population vaccination coverage.[5] Recent reports from Public Health England (PHE) have reported 81% influenza vaccination coverage and 69% pneumococcal vaccination coverage in adults aged 65 years and above, and 47% to 77% for shingles vaccination coverage in adults aged 71 and 78, respectively.[2-4] However, some evidence suggests that there could be vast differences in terms of vaccination coverage, potentially varying by geographical region, ethnicity, deprivation, household size, and health conditions.[2-4 6 7]

For the purposes of equitable public health strategy, it is important to understand factors associated with uptake of vaccinations, and refusal of vaccinations in the unvaccinated population. Prior studies have demonstrated differential uptake of existing vaccinations across sociodemographic groups, however, many studies have either studied single vaccinations, not captured the appreciable case-mix inherent to sociodemographic groups (such as by using broad ethnic categories), analysed a small set of relevant health conditions, and relied on potentially imprecise or biased self-report measures.[7-9] In addition, although household size is known to increase the risk of transmission for infectious diseases, evidence on the association between household size and vaccination uptake remains limited.[10] A few previous studies have suggested that individuals from larger households were less likely to be vaccinated, although these studies were small and mainly focused on childhood vaccinations.[11 12] Further, it is of interest to understand the pathway events leading to the lack of vaccine uptake, and to what extent these are driven by patient refusal.

Here, we evaluated factors associated with uptake and refusal of existing national vaccination programs (influenza, pneumococcal and shingles) in older adults (aged 65 years and above) in England and their associations with ethnic group, deprivation, household size, and health conditions.

For peer review only

Methods

Study population and data source

We performed a population-based cross-sectional study using QResearch (version 45). QResearch is a database with over 10 million current patients registered at more than 1800 practices in England. QResearch is an electronic healthcare primary care database in the UK with individual patient level records for general practices (GP) using the EMIS computer record system. The database captures information from GP consultations; among which includes patient demographics, socioeconomic status, diagnoses, laboratory test results, treatments and vaccinations. The database has good representation of the general population of England, particularly in terms of different ethnic groups with proportions close to those reported by Office for National Statistics.[13]

In this study, we included adults aged 65-99 years currently registered with 1,318 practices during the period 24th January 2020 to 31st October 2020, which comprised 2,054,463 of approximately 13.7 million patients aged 65 and over registered with a GP in England.[14] We assessed the uptake and refusal of influenza, pneumococcal, and shingles vaccines from 1st January 1989 until 31st October 2020 (last database update) as our main study outcome. As the shingles vaccination was rolled out nationally in England in 2013 for those aged 70 and up until 79 ,[15] we included in our shingles vaccine analysis only those aged 70 and above, excluding those aged 80 and above in year 2013 as they were not eligible at the time. Uptake was defined as the last recorded instance of receiving the vaccines of interest within the study period. This was mostly in GP surgeries (~99%), but also in-hospital or pharmacy administrations. Refusal was analysed in those with no record of vaccination, defined as last recorded instances of explicit refusal (74-82% of recorded code instances), consent not being given (18-26%), or non-attendance to a scheduled vaccination appointment (0.03-0.3%).[16] Outcomes were defined using code dictionaries comprising relevant Read and SNOMED codes as inputted into the EMIS software by healthcare practitioners.

We extracted demographic data including age, sex, self-reported ethnic group, Townsend deprivation index quintile,[17 18] geographical region within England (n=10, see Table 1), housing status and household size. Townsend deprivation score is an index of deprivation commonly used in the UK to measure socioeconomic status. It uses the following characteristics to measure deprivation by postcode; proportion of (1) unemployment, (2) non-car ownership, (3) non-home ownership, and (4) household crowding – with a higher score suggests greater deprivation. In this study the scores were reported in quintiles, i.e. first quintile indicates the least deprived. while fifth quintile indicates most deprived.

For peer review only - http://bmjopen.bmj.com/site/about/guidelines.xhtml

Ethnicity was grouped into 9 categories – White (White British, White Irish, Other White), Indian, Pakistani, Bangladeshi, Other Asian, Black Caribbean, Black African, Chinese, Other ethnic group (White & Black, White & Asian, Other mixed, Other Black, Other ethnic group). We also extracted data using GP Read and SNOMED codes from primary care records and ICD-10 codes from hospital records (where available) for diagnoses of asthma, chronic obstructive pulmonary disease (COPD), diabetes mellitus (type 1 and 2), hypertension, coronary heart disease (CHD), atrial fibrillation (AF), congenital heart disease, congestive cardiac failure (CCF), chronic neurological diseases (Parkinson's disease, epilepsy, cerebral palsy), learning disability, dementia, and severe mental illness (schizophrenia, severe depression, bipolar affective disorder and psychosis) and immune suppression (based on use of immunosuppressant medications). For each vaccination outcome (uptake and refusal), people with health conditions diagnosed prior to the vaccination outcome were defined as exposed, while those diagnosed with health conditions after the outcome were defined as unexposed. The most recently recorded BMI and smoking status were identified for each individual.

Analyses

Descriptive analyses compared the uptake and refusal of the three vaccinations of interest by ethnic group, Townsend deprivation quintiles, household size and individual health conditions. Percentage uptake of each vaccination in individual general practices was plotted to display between-region variations.

Multivariable logistic regression models examined associations between ethnic group, deprivation, household size, health conditions and vaccination uptake and refusal by calculating adjusted odds ratios (OR) and their 95% confidence intervals (CI). Clustered robust standard errors were used to account for clustering of individuals within general practices. Refusals were evaluated in never-receivers of each vaccine (no uptake). Individual models for each exposure (ethnic group, deprivation, household size, health conditions) and outcome (vaccination uptake and refusal for each vaccine) were fitted separately, allowing for adjustment of confounders: age, sex, geographical region, type of home, smoking status and/or BMI as relevant according to directed acyclic graphs (DAGs) - (i) Ethnicity – no adjustments; (ii) Deprivation - adjusted for age, sex, region, ethnicity, household size; (iii) Household size – adjusted for age, sex, region, ethnicity, deprivation, (iv) Health conditions - age, sex, region, ethnicity, deprivation, household size, house type, smoking and BMI. (Figure S1).

For peer review only - http://bmjopen.bmj.com/site/about/guidelines.xhtml

Missing data for ethnic group (18.5%), BMI (5.6%), deprivation quintiles (0.3%) and smoking status (1.0%) were multiply imputed using chained equations under the missing at random assumption. Five imputations were generated using a single rich imputation model incorporating all outcomes, exposures and confounder covariates. Models were fitted in each of the 5 imputed datasets with model coefficients and their standard errors pooled in accordance with Rubin's rules.[19] We also performed sensitivity analyses of results using complete-case analysis.

In addition, we performed post-hoc interaction analyses to explore potential interactive effects for vaccine uptake between ethnicity and deprivation, household size, and number of health conditions.

RECORD guidelines were used for reporting.[20] Statistical analyses were performed using STATA v17.0.[21]

For peer review only

Results

This study included 2,054,463 patients aged 65 years and older registered with 1,318 general practices. Characteristics of the study population are shown in Table 1 and S1. At least one influenza vaccine was received by 1,711,465 (83.3) patients, a pneumococcal vaccine by 1,391,228 (67.7%), and a shingles vaccine by 690,783 (53.4% of over 70s). Figure 1 showed a descriptive overview of the rate of vaccination uptake and refusals by different regions in England at the practice level. for example, the median level of shingles vaccine uptake in London practices was ~50%, compared to ~60% in East England. Overall, uptake of influenza vaccine (~80%) was the highest among all three vaccine types, followed by pneumococcal vaccine (~70%) and shingles vaccine (~50%) (Figure 1).

Vaccination uptake

Vaccination uptake differed by ethnicity, deprivation, household size, and health conditions (Figure 1). In multivariable analysis compared to the White population, those from Black Caribbean, Black African, Chinese and Other ethnic groups showed lower uptake for all three vaccines (Figure 2). Influenza vaccination uptake was significantly lower in Black Caribbean (OR 0.68, 95% CI: 0.64-0.71), Black African (OR 0.72; 0.68-0.77), Chinese (OR 0.49; 0.45-0.53) and 'Other ethnic group' (OR 0.63; 0.60-0.65), but there was significantly higher uptake in Indian (OR 1.21; 1.14-1.28), Pakistani (OR 1.39; 1.28-1.52), and Bangladeshi (OR 2.68; 2.38-3.01) ethnic groups compared to the White group.

There was a similar pattern observed for pneumococcal vaccination uptake: Black Caribbean (OR 0.70; 0.66-0.75), Black African (OR 0.56; 0.51-0.62), Chinese (OR 0.49; 0.45-0.53), 'Other ethnic group' (OR 0.58; 0.55-0.61), and also additionally for Other Asian (OR 0.87; 0.80-0.93). Pneumococcal vaccine uptake was significantly higher only in Bangladeshi ethnic group (OR 1.46; 1.29-1.65) compared to the White group. For shingles vaccine uptake, there was significantly lower uptake in all ethnic minority groups except in Indians (OR 0.98; 0.91-1.05).

For all three vaccines, vaccine uptake was generally lower among the more deprived, with the most deprived (lowest quintile) having 6% to 33% lower odds of vaccine uptake (ORs 0.67 to 0.94) compared to the most affluent. People in households with two people had 22% to 32% higher odds of having a vaccine compared to one-person households. However, the odds were lower in household sizes above

For peer review only - http://bmjopen.bmj.com/site/about/guidelines.xhtml

three, with people in households of 10 or more people having 17% to 63% lower odds to have vaccine uptake compared to one-person households.

The uptake of each vaccination was also generally associated with increasing number of health conditions; with asthma being associated with higher uptake of all three vaccines, while atrial fibrillation, congestive cardiac failure, dementia, severe mental illness were being associated with lower uptake of all three vaccines. Individuals with COPD, diabetes and immunosuppression were also more likely to be associated with higher uptake of both influenza and pneumococcal vaccines but not for shingles vaccine (Figure S2).

Vaccination refusals in the unvaccinated

There were consistently significantly higher odds of vaccine refusal amongst the Black Caribbean group compared to the White group for all three vaccines; influenza (OR 1.45; 1.34-1.56), pneumococcal (OR 1.29; 1.14-1.46) and shingles (OR 1.35; 1.23-1.49). Indian, Pakistani, Bangladeshi, Other Asian, Black African, Chinese, and Other ethnic groups were significantly less likely to refuse all three vaccines compared to White ethnic group, except for Pakistani and Bangladeshi, which showed no significant association with shingles vaccine refusal. (Figure 3)

There was a general trend of refusal with increasing deprivation, particularly with shingles vaccine in the two most deprived quintiles, OR 1.21; 1.15-1.28, and OR 1.23; 1.14-1.33 (4th and 5th deprivation quintiles, respectively). Higher household size was associated with lower odds of refusal of all three vaccines in households of 3+ people and more. (Figure 3)

In individuals with three or more health conditions, the odds of refusal were: influenza vaccine (OR 10.29; 7.38-14.37), pneumococcal vaccine (OR 2.55; 2.24-2.90), shingles vaccine (1.60; 1.48-1.73). Individuals with type 2 diabetes consistently showed higher vaccine refusal for all three vaccines and individuals with COPD was also associated with higher refusal for influenza and pneumococcal vaccines. (Figure S3)

For peer review only

For peer review only - http://bmjopen.bmj.com/site/about/guidelines.xhtml

Additional analyses

Further, we explored the interactions for vaccine uptake between ethnicity and deprivation, house size and number of health conditions. First, results suggested that certain ethnic minority groups who were more deprived could be more likely to receive a vaccine, particularly Bangladeshi and Black African. (Figure S4) Second, across all three vaccines evaluated, Bangladeshi living in larger households could be more likely to receive a vaccine (Figure S5) Third, vaccine uptake was generally more likely in individuals with higher number of health conditions, although the magnitude of effect varied slightly across different ethnic groups. (Figure S6)

Finally, we performed sensitivity analyses to evaluate associations of vaccine uptake and refusal using complete-case analyses. In this analysis we excluded individuals with missing information on covariates i.e. ethnicity, deprivation, BMI and smoking. Results on Figure S7-8 showed that estimates were comparable with multiply imputed analysis presented as our main findings above.

For peer review only

For peer review only - http://bmjopen.bmj.com/site/about/guidelines.xhtml

Discussion

Summary

In this study, we observed generally lower uptake of influenza, pneumococcal and shingles vaccinations in particular ethnic minority groups and deprived populations. Black Caribbean, Black African, Chinese and Other ethnic groups consistently showed lower uptake of all three vaccines studied compared to the White ethnic group. In the unvaccinated population, the Black Caribbean ethnic group consistently showed lower vaccine uptake and increased odds of vaccine refusal for all three vaccines. More deprived populations also showed lower vaccine uptake with higher refusals in the unvaccinated. Household sizes above 3 persons were associated with lower vaccine uptake, but were not associated with higher refusal. Further, a lower number of pre-existing health conditions was generally associated with lower odds of vaccine uptake, although this was not reflected in terms of higher odds of refusal.

Comparison with existing literature

Our observations that influenza vaccination uptake is inversely correlated with deprivation and varies across ethnic groups build upon results from a recent study of adults between 2011-2016 using the CPRD database.[7] This study analysed seasonal influenza vaccination uptake across 5 'seasons' and similarly found that in the over 65s, Black individuals were significantly less likely than White individuals to receive this vaccination. However, our study finds that South Asians may be more likely to have higher uptake of influenza vaccine, which may warrant further qualitative study to examine potential socioeconomic and behavioural factors driving this observation. Our examination of three vaccinations within a larger sample size (over 2 million vs. 611,000), a more granular categorisation of ethnic groups (9 vs. 4) and regions (10 vs. 4), improved handling of missing data, and our analysis of vaccination refusals in the unvaccinated substantially improves our understanding of these complex public health behaviours. Our results showed that although four ethnic minority groups (Black Caribbean, Black African, Chinese and Other ethnic group) had lower uptake of influenza vaccine, only the Black Caribbean group showed increased odds of refusal among the unvaccinated.

We also found lower vaccine uptake in household sizes above 3 persons, although they also showed lower refusals in the unvaccinated population. This suggests that lower vaccine uptake in larger households could be driven by barriers to vaccine uptake other than due to refusal alone. A study in Hong Kong showed that vaccine uptake in the elderly living with younger family members had lower

For peer review only - http://bmjopen.bmj.com/site/about/guidelines.xhtml

vaccine uptake compared to elderly living alone or living with other elderly household members.[6] This calls for further ethnographic research to explore social and household characteristics including age structure of household members and its potential association with vaccine uptake in the elderly in England.

Higher uptake of influenza and pneumococcal vaccinations in individuals with asthma, COPD, diabetes and immunosuppression could be related to clinical guidelines where individuals in these clinical risk groups would be more likely to be offered a vaccine by their health care providers.[22 23] On the contrary, lower vaccine uptake in those with fewer health conditions could potentially be attributable to reduced contact with health services in the healthier population and hence, reduced likelihood to receive 'opportunistic' vaccination offers. Despite that, it is worth noting that our study also found that in the unvaccinated population there remains significant refusal in those with type-2 diabetes and COPD. Possibly relevant factors could be resistance to lifestyle and behaviour changes, in which individuals with diabetes and COPD who might be more likely to have unhealthy lifestyles e.g. smoking[24 25] might also be less receptive to health interventions i.e. vaccines. However, this finding needs confirmation in other studies. In addition, interaction analyses from our study showed that certain ethnic minority groups i.e. Bangladeshis who were more deprived and living in larger households were more likely to receive a vaccine. This could potentially be due to availability of outreach programs organised by local communities and GPs in these areas to create awareness and provide health education.[26 27]

Strengths and limitations

Use of the QResearch database offered a population-representative population with accurately coded data, enabling capture of vaccinations occurring outside general practice (such as in pharmacies), as well as recorded invitations to vaccination sent by general practices and patient refusals. This permitted a robust evaluation of not only uptake, but also possible contributory mechanisms leading to uptake behaviours. Limitations include the lack of recording of variables such as religion, personal beliefs and reasons for refusal that predicate vaccine hesitancy in our sample. Further, our dataset also did not capture literacy levels, language barriers, access and education status, and hence were not able to evaluate the impact of these socioeconomic factors on vaccination uptake and refusal. These could be important factors influencing the complex decision-making and behavioural aspects and hence would warrant further qualitative and ethnography studies. Classification of vaccination-related endpoints was reliant on individual practitioners using Read and SNOMED codes on the EMIS software system;

For peer review only

however, as GP surgeries are financially incentivised through 'Quality Outcome Framework' payments to record vaccination services and we used an appropriately wide range of codes in our endpoint definitions, the risk of misclassification may be low.

Implications for research and practice

Two key principles in health inequalities are Tudor-Hart's inverse care law,[28] where service provision is inversely proportional to the need for it, and the inverse equity hypothesis, which posits that new healthcare interventions are most likely to be taken up by those in less need and thus exacerbate pre-existing inequality in the short term. Our study may help inform policymakers regarding reducing inequity in the uptake of the studied vaccines, and tailor public health messaging to diverse communities. Elucidating the extent to which ethnic patterns in vaccine refusal are driven by cultural perceptions, institutional mistrust, variation in penetrance of misinformation, and structural barriers e.g. transport, language and occupational barriers in different ethnic groups requires further study in robust surveys and qualitative research. This may inform tailoring of information dissemination strategies and misinformation countermeasures to specific groups and geographical areas. Furthermore, judicious, longitudinal monitoring of the uptake and refusal rates of vaccines in different ethnic and social groups should enable real-time assessment of developing inequalities, which may inform adaptive public health strategies. Data from this may help develop strategies for increasing uptake in these groups including developing information about vaccines in different languages for use by community leaders, faith groups, local health care providers and community champions.[29]

Conclusions

Certain ethnic minority, deprived populations, large households and healthier individuals were less likely to receive a vaccine, although in the unvaccinated population, higher odds of refusal was only associated with ethnicity and deprivation but not larger households nor healthier individuals. Understanding these associations may inform tailored public health messaging to different communities for equitable implementation of vaccination programs.

For peer review only - http://bmjopen.bmj.com/site/about/guidelines.xhtml

Author contributions

JHC and HDM obtained funding for the study. JHC extracted the data. PST, MP and AKC led data analysis and wrote first draft. PST, MP and AKC, HDM, DS, TAR, CG, FZ, BRS, CC, SJG, KK and JHC interpreted results, participated in critical revisions of manuscript and approved the final version.

Declaration of interests

PST reports previous consultation with AstraZeneca and Duke-NUS outside the submitted work. KK is a Member of the Scientific Advisory Group for Emergencies (SAGE), Member of Independent SAGE, Director of the University of Leicester Centre for Black Minority Health and Trustee of the south Asian Health Foundation. JHC is a member of several SAGE committees and chair of the risk stratification subgroup of the NERVTAG. She is unpaid director of QResearch and founder and former medical director of ClinRisk Ltd (outside the submitted work). MP, AKC, HDM, DS, TAR, FZ, BRS, SJG, CC, CG have no interests to declare.

For peer review only

Acknowledgments

Funding – This project was funded by a grant from the Medical Research Council (MRC Grant Ref: MR/V027778/1). JHC has received grants from the National Institute for Health Research Biomedical Research Centre, Oxford, John Fell Oxford University Press Research Fund, Cancer Research UK (grant number C5255/A18085) through the Cancer Research UK Oxford Centre, and the Oxford Wellcome Institutional Strategic Support Fund (204826/Z/16/Z) during the conduct of the study, is an unpaid director of QResearch, a not-for-profit organisation which is a partnership between the University of Oxford and EMIS Health who supply the QResearch database used for this work, and is a founder and shareholder of ClinRisk Ltd and was its medical director until 31 May 2019; ClinRisk produces open and closed source software to implement clinical risk algorithms (outside this work) into clinical computer systems. AKC is funded by a Clinical Research Training Fellowship from Cancer Research UK (grant C2195/A31310). KK is supported by NIHR Applied Collaboration East Midlands and NIHR Leicester Biomedical Research Centre. The University of Cambridge has received salary support in respect of SJG from the NHS in the East of England through the Clinical Academic Reserve. We acknowledge the contribution of EMIS practices who contribute to QResearch and EMIS Health and the Universities of Nottingham and Oxford for expertise in establishing, developing, or supporting the QResearch database. This project involves data derived from patient level information collected by

the NHS, as part of the care and support of cancer patients. The data are collated, maintained, and quality assured by the National Cancer Registration and Analysis Service, which is part of Public Health England (PHE). Access to the data was facilitated by the PHE Office for Data Release. The Hospital Episode Statistics data used in this analysis are reused by permission from NHS Digital, which retains the copyright in that data. We thank the Office for National Statistics (ONS) for providing the mortality data. NHS Digital, PHE, and the ONS bear no responsibility for the analysis or interpretation of the data. The investigators acknowledge the philanthropic support of the donors to the University of Oxford's COVID-19 Research Response Fund.

Role of the funding source

The funder had no role in the study design, in the collection, analysis, or interpretation of data, in the writing of the report, or in the decision to submit the paper for publication.

Data statement

To guarantee the confidentiality of personal and health information, only the authors have had access to the data during the study in accordance with the relevant license agreements. Access to QResearch data is according to the information on the QResearch website (www.qresearch.org).

Ethics approval

This was part of a larger project which has been independently peer-reviewed and received ethics approval by the QResearch Scientific board (REC 18/EM/0400; project reference OX102).

Patient and public involvement reporting

Two public representatives advised on interest and appropriateness of the research questions, were involved in writing the protocol for the wider study, and input on lay-summaries describing the planned study.

For peer review only - http://bmjopen.bmj.com/site/about/guidelines.xhtml

References

1. Juliet M. Bartleson DR, Anthony J. Covarrubias, David Furman, Daniel A. Winer & Eric Verdin SARS-CoV-2, COVID-19 and the aging immune system. *Nat Aging* 2021;1:769–82.
2. England PH. Pneumococcal Polysaccharide Vaccine (PPV) coverage report, England, April 2019 to March 2020 2020 [Available from: https://assets.publishing.service.gov.uk/government/uploads/system/uploads/attachment_data/file/899458/hpr1320_ppv-vc.pdf
3. England PH. Seasonal influenza vaccine uptake in GP patients: winter season 2020 to 2021: Public Health England; 2021 [Available from: https://assets.publishing.service.gov.uk/government/uploads/system/uploads/attachment_data/file/996033/Annual-Report_SeasonalFlu-Vaccine_GPs_2020_to_2021.pdf
4. England PH. Shingles vaccine coverage report (adults eligible from April to September 2020 and vaccinated to end of December 2020): England

Quarter 2 report of financial year 2020 to 2021 2021 [Available from: https://assets.publishing.service.gov.uk/government/uploads/system/uploads/attachment_data/file/955523/SP_hpr0321_SHNGLS-vc_Q2-final-3_refs.pdf

5. Europe W. Evaluation of seasonal influenza vaccination policies and coverage in the WHO European Region 2014 [Available from: https://www.euro.who.int/__data/assets/pdf_file/0003/241644/Evaluation-of-seasonal-influenza-vaccination-policies-and-coverage-in-the-WHO-European-Region.pdf
6. Chan DP, Wong NS, Wong EL, et al. Household characteristics and influenza vaccination uptake in the community-dwelling elderly: a cross-sectional study. *Prev Med Rep* 2015;2:803-8. doi: 10.1016/j.pmedr.2015.09.002 [published Online First: 2015/09/21]
7. Loiacono MM, Mahmud SM, Chit A, et al. Patient and practice level factors associated with seasonal influenza vaccine uptake among at-risk adults in England, 2011 to 2016: An age-stratified retrospective cohort study. *Vaccine X* 2020;4:100054. doi: 10.1016/j.jvacx.2020.100054 [published Online First: 2020/01/13]
8. Coupland C, Harcourt S, Vinogradova Y, et al. Inequalities in uptake of influenza vaccine by deprivation and risk group: time trends analysis. *Vaccine* 2007;25(42):7363-71. doi: 10.1016/j.vaccine.2007.08.032 [published Online First: 2007/09/05]
9. Fisher H, Trotter CL, Audrey S, et al. Inequalities in the uptake of human papillomavirus vaccination: a systematic review and meta-analysis. *Int J Epidemiol* 2013;42(3):896-908. doi: 10.1093/ije/dyt049 [published Online First: 2013/04/25]
10. House T, Keeling MJ. Household structure and infectious disease transmission. *Epidemiol Infect* 2009;137(5):654-61. doi: 10.1017/S0950268808001416 [published Online First: 2008/10/08]
11. Rockliffe L, Waller J, Marlow LA, et al. Role of ethnicity in human papillomavirus vaccination uptake: a cross-sectional study of girls from ethnic minority groups attending London schools. *BMJ Open* 2017;7(2):e014527. doi: 10.1136/bmjopen-2016-014527 [published Online First: 2017/02/23]
12. Sarker AR, Akram R, Ali N, et al. Coverage and factors associated with full immunisation among children aged 12-59 months in Bangladesh: insights from the nationwide cross-sectional demographic and health survey. *BMJ Open* 2019;9(7):e028020. doi: 10.1136/bmjopen-2018-028020 [published Online First: 2019/07/09]
13. GOV.UK. Population of England and Wales [Available from: https://www.ethnicity-facts-figures.service.gov.uk/uk-population-by-ethnicity/national-and-regional-populations/population-of-england-and-wales/latest.

For peer review only

14. Digital N. Patients registered at a GP practice [Available from: https://digital.nhs.uk/data-and-information/data-tools-and-services/data-services/general-practice-data-hub/patients-registered-at-a-gp-practice
15. Gov.UK. Vaccination against shingles guide [updated 2 December 2021. Available from: https://www.gov.uk/government/publications/shingles-vaccination-for-adults-aged-70-or-79-years-of-age-a5-leaflet/vaccination-against-shingles-guide accessed 14 April 2022.
16. QResearch Web [cited 2021. Available from: https://qweb.qresearch.org.
17. Townsend P , . DN. The Black report: Penguin 1982.
18. 2011 UK Townsend Deprivation Scores - Townsend Deprivation Scores Report. 2011
19. Rubin D. Multiple Imputation for Non-response in Surveys: John Wiley 1987.
20. Benchimol EI, Smeeth L, Guttmann A, et al. The REporting of studies Conducted using Observational Routinely-collected health Data (RECORD) statement. *PLoS Med* 2015;12(10):e1001885. doi: 10.1371/journal.pmed.1001885 [published Online First: 2015/10/06]
21. *Stata Statistical Software: Release 17* [program]. College Station, TX: StataCorp LLC.: StataCorp. .
22. Green Book - Pneumococcal 2018
23. Seasonal influenza vaccine uptake in GP patients: winter season 2020 to 2021: Public Health England; 2021 [Available from: https://assets.publishing.service.gov.uk/government/uploads/system/uploads/attachment_data/file/996033/Annual-Report_SeasonalFlu-Vaccine_GPs_2020_to_2021.pdf accessed 20 April 2022.
24. Bhatt SP, Kim YI, Harrington KF, et al. Smoking duration alone provides stronger risk estimates of chronic obstructive pulmonary disease than pack-years. *Thorax* 2018;73(5):414-21. doi: 10.1136/thoraxjnl-2017-210722 [published Online First: 20180111]
25. Śliwińska-Mossoń M, Milnerowicz H. The impact of smoking on the development of diabetes and its complications. *Diab Vasc Dis Res* 2017;14(4):265-76. doi: 10.1177/1479164117701876 [published Online First: 20170408]
26. Abbott S, Riga M. Delivering services to the Bangladeshi community: the views of healthcare professionals in East London. *Public Health* 2007;121(12):935-41. doi: 10.1016/j.puhe.2007.04.014 [published Online First: 20070725]
27. Latif S, Ahmed I, Amin MS, et al. Exploring the potential impact of health promotion videos as a low cost intervention to reduce health inequalities: a pilot before and after study on Bangladeshis in inner-city London. *London J Prim Care (Abingdon)* 2016;8(4):66-71. doi: 10.1080/17571472.2016.1208382 [published Online First: 20160803]
28. Victora CG, Joseph G, Silva ICM, et al. The Inverse Equity Hypothesis: Analyses of Institutional Deliveries in 286 National Surveys. *Am J Public Health* 2018;108(4):464-71. doi: 10.2105/AJPH.2017.304277 [published Online First: 2018/02/22]
29. Hanif W, Ali SN, Patel K, et al. Cultural competence in covid-19 vaccine rollout. *BMJ* 2020(371):m4845. doi: doi.org/10.1136/bmj.m4845

For peer review only - http://bmjopen.bmj.com/site/about/guidelines.xhtml

Table 1: Characteristics of study population in patients aged 65+ (70+ for shingles)

Characteristics		Study population	Vaccine uptake		
		Overall	Influenza	Pneumococcal	Shingles[a]
Total	N (row %)	2054463	1711465 (83.3)	1391228 (67.7)	690783 (53.4)
Age	Mean (SD)	75.5 (7.7)	76.3 (7.7)	77.1 (7.5)	77.2 (4.4)
	65-69	541272 (26.3)	373566 (21.8)	232831 (16.7)	-
	70-79	922198 (44.9)	793150 (46.3)	665037 (47.8)	469684 (68.0)
	80-89	471167 (22.9)	434074 (25.4)	395456 (28.4)	221099 (32.0)
	90-99	119826 (5.8)	110675 (6.5)	97904 (7.0)	-
Sex	Female	1100957 (53.6)	926592 (54.1)	749022 (53.8)	365203 (52.9)
	Male	953506 (46.4)	784873 (45.9)	642206 (46.2)	325580 (47.1)
Ethnicity	White	1522868 (74.1)	1293856 (75.6)	1064331 (76.5)	539237 (78.1)
	Indian	35618 (1.7)	31062 (1.8)	25454 (1.8)	11293 (1.6)
	Pakistani	17555 (0.9)	15588 (0.9)	12090 (0.9)	4388 (0.6)
	Bangladeshi	8138 (0.4)	7635 (0.4)	6264 (0.5)	2076 (0.3)
	Other Asian	17848 (0.9)	15171 (0.9)	11890 (0.9)	5135 (0.7)
	Black Caribbean	22859 (1.1)	18010 (1.1)	14102 (1.0)	5791 (0.8)
	Black African	16880 (0.8)	13530 (0.8)	9545 (0.7)	3518 (0.5)
	Chinese	6553 (0.3)	4835 (0.3)	3507 (0.3)	1502 (0.2)
	Other ethnic groups	25410 (1.2)	19778 (1.2)	14569 (1.0)	5832 (0.8)
	Ethnicity not recorded	380734 (18.5)	292000 (17.1)	229476 (16.5)	112011 (16.2)
Region	East Midlands	46002 (2.2)	38777 (2.3)	30526 (2.2)	16779 (2.4)
	East of England	93217 (4.5)	77645 (4.5)	64843 (4.7)	34167 (4.9)
	London	322941 (15.7)	261176 (15.3)	204112 (14.7)	92174 (13.3)
	North East	47496 (2.3)	40081 (2.3)	33271 (2.4)	15848 (2.3)
	North West	417970 (20.3)	354779 (20.7)	292600 (21.0)	140099 (20.3)
	South Central	283054 (13.8)	239109 (14.0)	199347 (14.3)	102632 (14.9)
	South East	268594 (13.1)	220952 (12.9)	179031 (12.9)	91516 (13.2)
	South West	256384 (12.5)	213037 (12.4)	169824 (12.2)	87179 (12.6)
	West Midlands	237881 (11.6)	197414 (11.5)	161606 (11.6)	81942 (11.9)
	Yorkshire & Humber	80924 (3.9)	68495 (4.0)	56068 (4.0)	28447 (4.1)
Deprivation quintile	1 (most affluent)	674004 (32.8)	569701 (33.3)	471575 (33.9)	251660 (36.4)
	2	547862 (26.7)	456956 (26.7)	373336 (26.8)	191172 (27.7)
	3	385476 (18.8)	318962 (18.6)	258842 (18.6)	123090 (17.8)
	4	267458 (13.0)	219941 (12.9)	175665 (12.6)	78550 (11.4)
	5 (most deprived)	174280 (8.5)	141551 (8.3)	108526 (7.8)	44651 (6.5)
	Not recorded	5383 (0.3)	4354 (0.3)	3284 (0.2)	1660 (0.2)
Home category	Neither in care home nor homeless	2005725 (97.6)	1665389 (97.3)	1356313 (97.5)	682316 (98.8)
	Care home	47655 (2.3)	45263 (2.6)	34352 (2.5)	8301 (1.2)
	Homeless	1083 (0.1)	813 (<0.01)	563 (<0.01)	166 (<0.01)
Household size	1 person	875588 (42.6)	726447 (42.4)	596361 (42.9)	285715 (41.4)
	2 people	849357 (41.3)	721411 (42.2)	594481 (42.7)	326499 (47.3)

For peer review only - http://bmjopen.bmj.com/site/about/guidelines.xhtml

	3-5 people	255089 (12.4)	199611 (11.7)	152373 (11.0)	65031 (9.4)
	6-9 people	30961 (1.5)	24934 (1.5)	18767 (1.3)	6678 (1.0)
	10 or more	43468 (2.1)	39062 (2.3)	29246 (2.1)	6860 (1.0)
Number of health conditions[c]	0	667163 (32.5)	483507 (28.3)	566398 (40.7)	213919 (31.0)
	1	786798 (38.3)	671330 (39.2)	559648 (40.2)	281353 (40.7)
	2	428751 (20.9)	393220 (23.0)	215126 (15.5)	145583 (21.1)
	3+	171751 (8.4)	163408 (9.5)	50056 (3.6)	49928 (7.2)

[a]Percentage calculated using denominator of shingles eligible population, n = 1,294,176. Percentages are column percentages unless otherwise indicated. SD: standard deviation. [c]Counts only based on conditions included in this study.

For peer review only

Figure labels and footnotes

Figure 1: Box and whiskers diagrams summarising influenza, pneumococcal and shingles vaccination uptake/refusal rates in practices across different regions in England. The mid-line of box represents median uptake/refusal rate, lower and upper boundaries of box represent first and third quartile, lower and upper whiskers represent minimum and maximum rates. Each individual dot was also presented to represent individual practice uptake/refusal rate.

Figure 2: Associations of ethnicity, deprivation, household size and number of health conditions on influenza, pneumococcal and shingles vaccine uptake.

Footnote: Logistic models for ethnicity, deprivation, household size and health conditions were run separately as each exposure factor required different sets of adjustment variables as informed by DAG evaluation. The following adjustment covariates were included in each of these models as the following: (1) Ethnicity – no adjustment; (2) Deprivation - adjusted for age, sex, region, ethnicity, household size; (3) Household size – adjusted for age, sex, region, ethnicity, deprivation, (4) Health conditions – adjusted for age, sex, region, ethnicity, deprivation, household size, house type, smoking and BMI.

Figure 3: Associations of ethnicity, deprivation, household size and number of health conditions on influenza, pneumococcal and shingles vaccine refusal in the unvaccinated population.

Footnote: Logistic models for ethnicity, deprivation, household size and health conditions were run separately as each exposure factor required different sets of adjustment variables as informed by DAG evaluation. The following adjustment covariates were included in each of these models as the following: (1) Ethnicity – no adjustment; (2) Deprivation - adjusted for age, sex, region, ethnicity, household size; (3) Household size – adjusted for age, sex, region, ethnicity, deprivation, (4) Health conditions – adjusted for age, sex, region, ethnicity, deprivation, household size, house type, smoking and BMI.

For peer review only

For peer review only - http://bmjopen.bmj.com/site/about/guidelines.xhtml

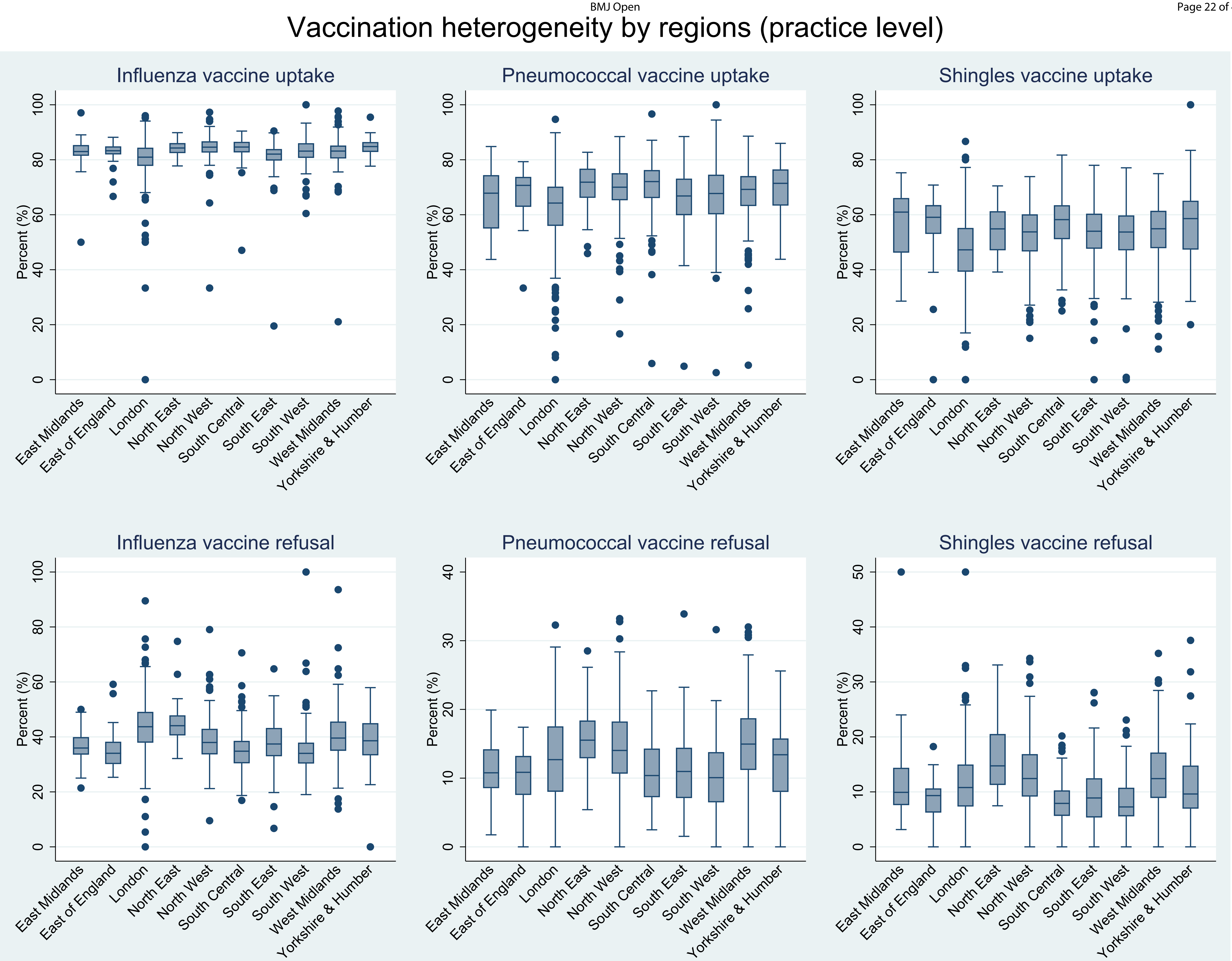
Vaccination heterogeneity by regions (practice level)
Influenza vaccine uptake
Pneumococcal vaccine uptake
Shingles vaccine uptake
Influenza vaccine refusal
Pneumococcal vaccine refusal
Shingles vaccine refusal
Percent (%)
0
10
20
30
40
50
60
80
100
East Midlands
East of England
London
North East
North West
South Central
South East
South West
West Midlands
Yorkshire & Humber

Uptake

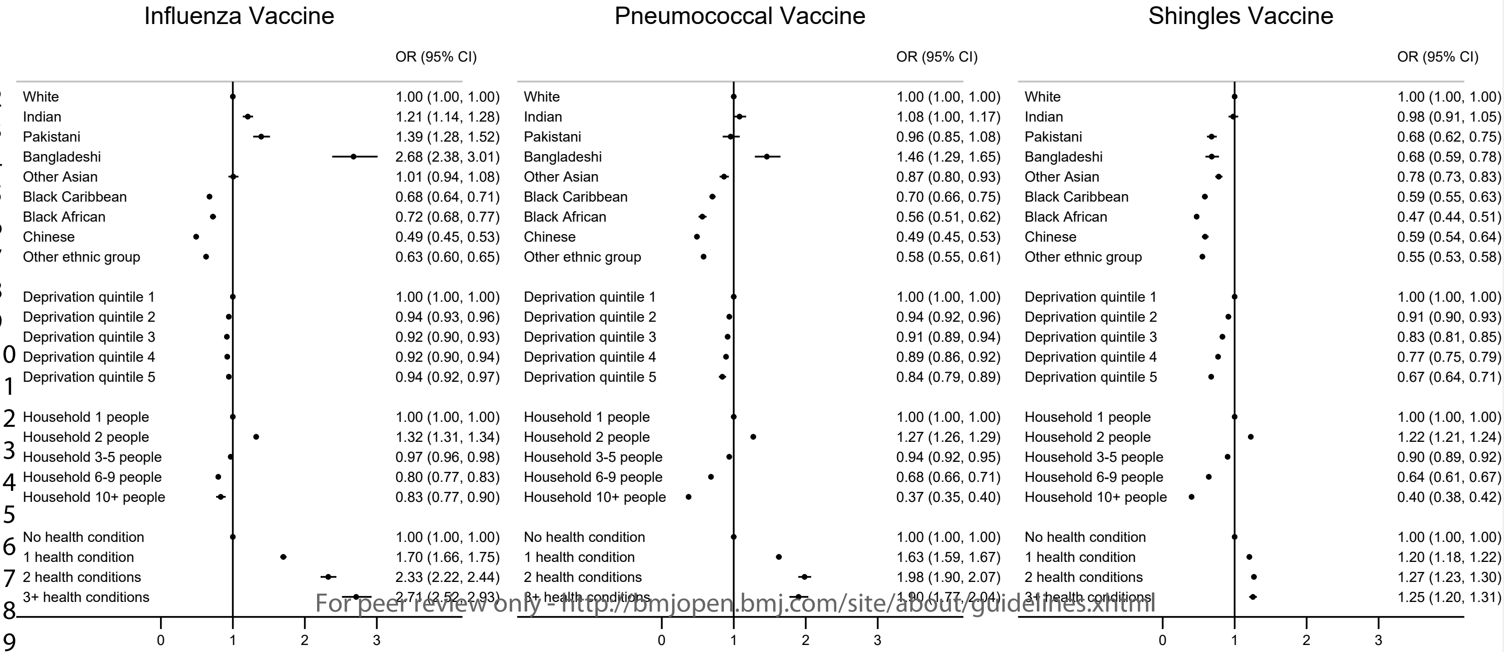

For peer review only - http://bmjopen.bmj.com/site/about/guidelines.xhtml

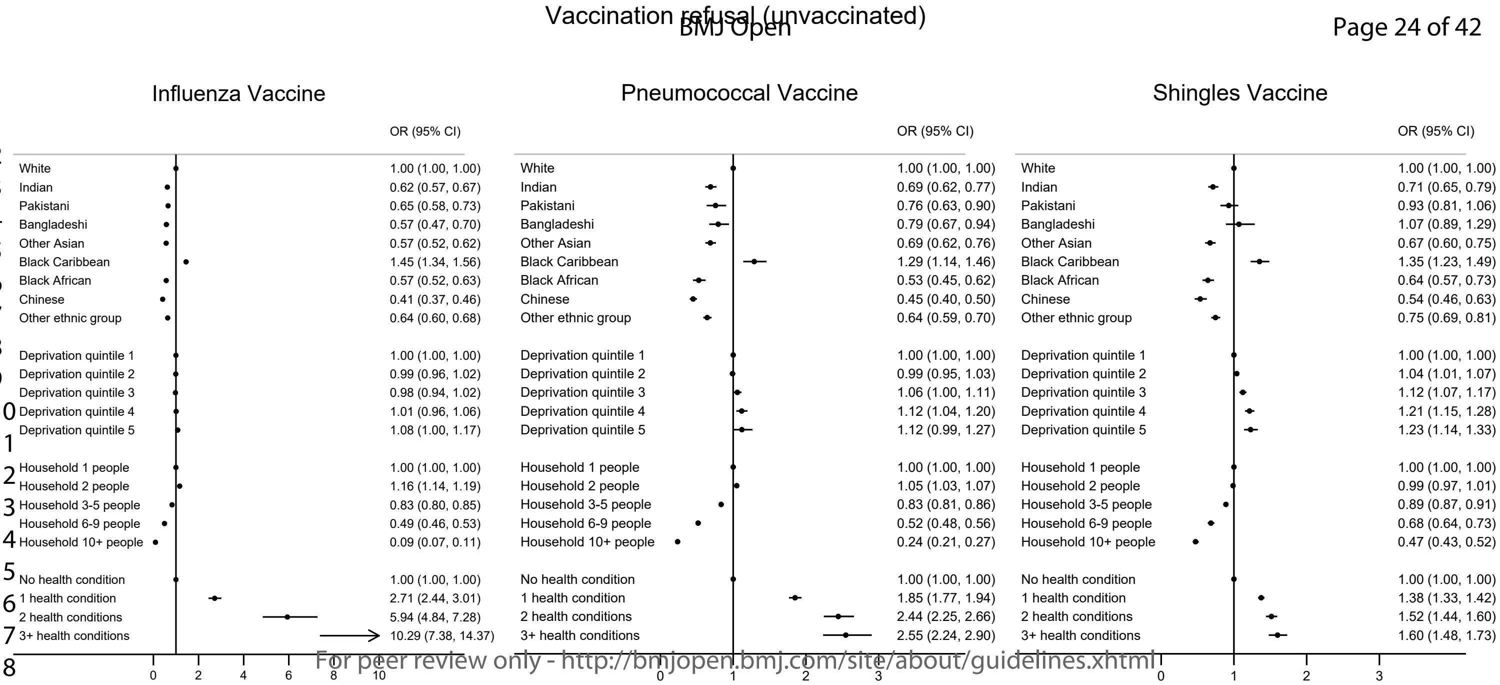

For peer review only - http://bmjopen.bmj.com/site/about/guidelines.xhtml

Supplement

Table S1: Characteristics of study population - lifestyle and health conditions

Characteristics		Study population	Vaccine uptake		
		Overall	Influenza	Pneumococcal	Shingles[a]
Total	N (row %)	2054463	1711465 (83.3)	1391228 (67.7)	690783 (53.4)
Body mass index	<18.5	36406 (1.8)	31088 (1.8)	25321 (1.8)	9351 (1.4)
	18.5-25	615113 (29.9)	515261 (30.1)	421175 (30.3)	204730 (29.6)
	25-30	754859 (36.7)	641998 (37.5)	528282 (38.0)	273645 (39.6)
	30-35	361993 (17.6)	310919 (18.2)	254754 (18.3)	128653 (18.6)
	35-40	121452 (5.9)	105199 (6.1)	85649 (6.2)	41349 (6.0)
	>=40	48792 (2.4)	42948 (2.5)	34151 (2.5)	15061 (2.2)
	Not recorded	115848 (5.6)	64052 (3.7)	41896 (3.0)	17994 (2.6)
Smoking	Non-smoker	1143669 (55.7)	955785 (55.8)	773504 (55.6)	383407 (55.5)
	Ex-smoker	712384 (34.7)	618783 (36.2)	516754 (37.1)	265778 (38.5)
	Current smoker	177685 (8.6)	132076 (7.7)	98773 (7.1)	40903 (5.9)
	Not recorded	20725 (1.0)	4821 (0.3)	2197 (0.2)	695 (0.1)
Health conditions	Asthma	254110 (12.4)	235822 (13.8)	162658 (11.7)	89598 (13.0)
	Chronic obstructive pulmonary disease	160907 (7.8)	150873 (8.8)	66827 (4.8)	52655 (7.6)
	Type-1 diabetes	6253 (0.3)	5908 (0.3)	4243 (0.3)	1882 (0.3)
	Type-2 diabetes	353860 (17.2)	327748 (19.2)	183136 (13.2)	120912 (17.5)
	Hypertension	1013241 (49.3)	901041 (52.6)	559319 (40.2)	360378 (52.2)
	Dementia	86868 (4.2)	81151 (4.7)	8622 (0.6)	10989 (1.6)
	Parkinson's disease	20720 (1.0)	18825 (1.1)	4635 (0.3)	5467 (0.8)
	Epilepsy	38404 (1.9)	33738 (2.0)	19335 (1.4)	10874 (1.6)
	Cerebral palsy	1041 (0.1)	929 (0.1)	598 (0.0)	233 (0.0)
	Learning disability	39959 (1.9)	36644 (2.1)	9192 (0.7)	9897 (1.4)
	Severe mental illness	243791 (11.9)	210885 (12.3)	133322 (9.6)	73294 (10.6)
	Coronary heart disease	294490 (14.3)	273488 (16.0)	153850 (11.1)	101948 (14.8)
	Atrial fibrillation	196503 (9.6)	180461 (10.5)	53438 (3.8)	55647 (8.1)
	Congestive cardiac failure	85674 (4.2)	79600 (4.7)	19891 (1.4)	20144 (2.9)
	Congenital heart disease	14739 (0.7)	13500 (0.8)	6590 (0.5)	4938 (0.7)
	Immunosuppression	17339 (0.8)	16188 (0.9)	8622 (0.6)	3445 (0.5)

[a]Percentage calculated using denominator of shingles eligible population, n = 1,294,176. [b] Comorbidities diagnosed prior to vaccinations in those vaccinated. Percentages are column percentages unless otherwise indicated. SD: standard deviation.

For peer review only

Figure S1: Directed acyclic graphs (DAGs) modelling exposures and corresponding outcomes. DAGs were used to map out the relationships between exposure and outcome of interest, and how they were related to other covariates to evaluate which variables were considered a confounder and would need to be adjusted for in the regression models.

Interpretation of DAGs
Green circles denote exposure and blue circle with "I" denote outcome.
White circles denote adjusted covariates while other blue circles denote variables not for adjustment in each model.

Model 1:
Exposure: Ethnicity
Outcome: Vaccination uptake/refusal
Confounder adjustment: None (no other variables were identified as a confounder for the association between ethnicity and vaccine uptake/refusal)

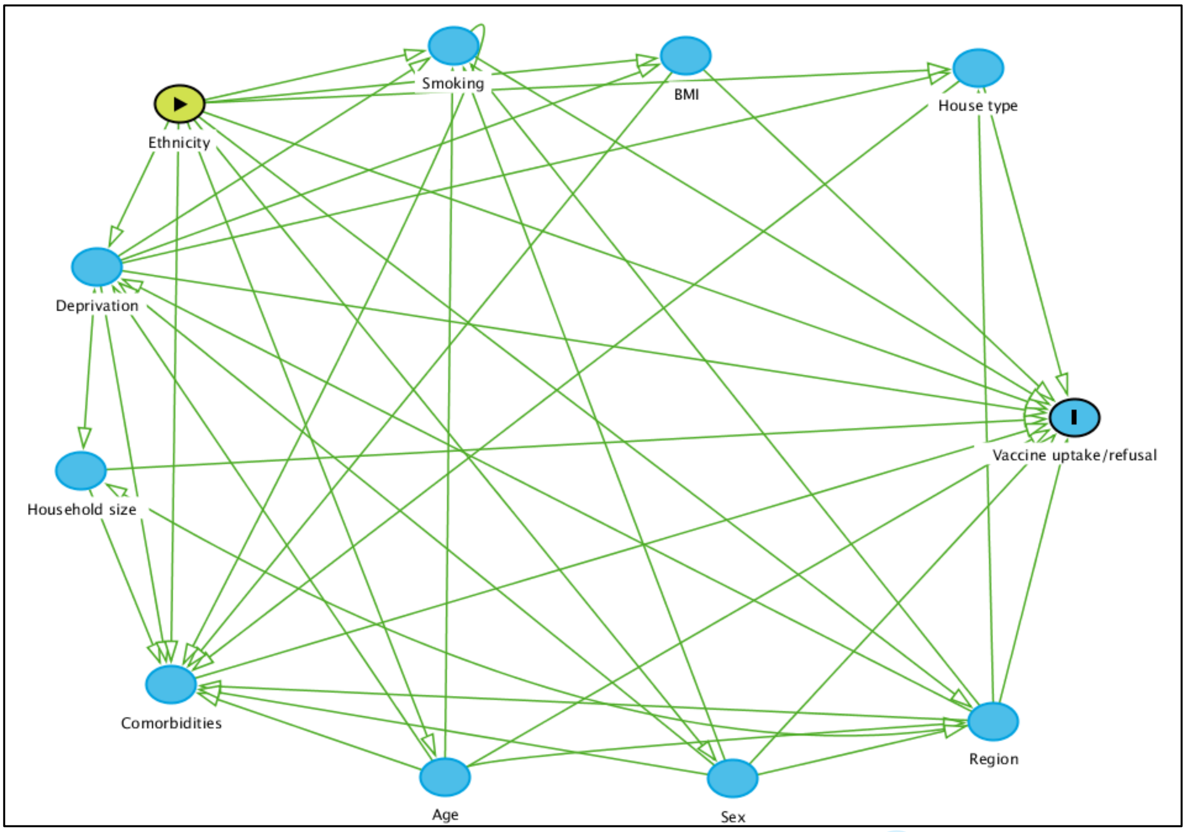

only

Model 2:
Exposure: Deprivation
Outcome: Vaccination uptake/refusal
Confounder adjustment: age, sex, region, ethnicity, household size (identified as confounders for the association between deprivation and vaccine uptake/refusal)

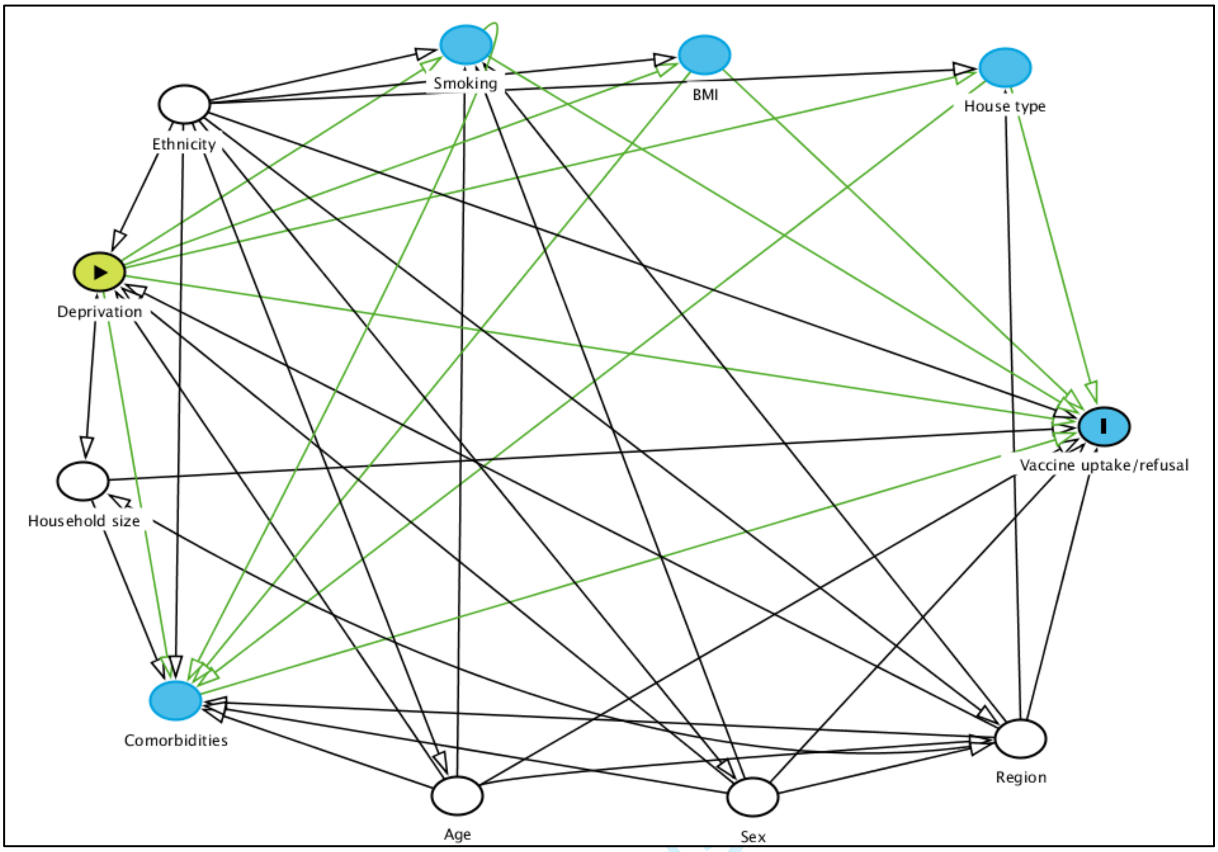

Model 3:
Exposure: Household size
Outcome: Vaccination uptake/refusal
Confounder adjustment: age, sex, region, ethnicity, deprivation (identified as confounders for the association between household size and vaccine uptake/refusal)

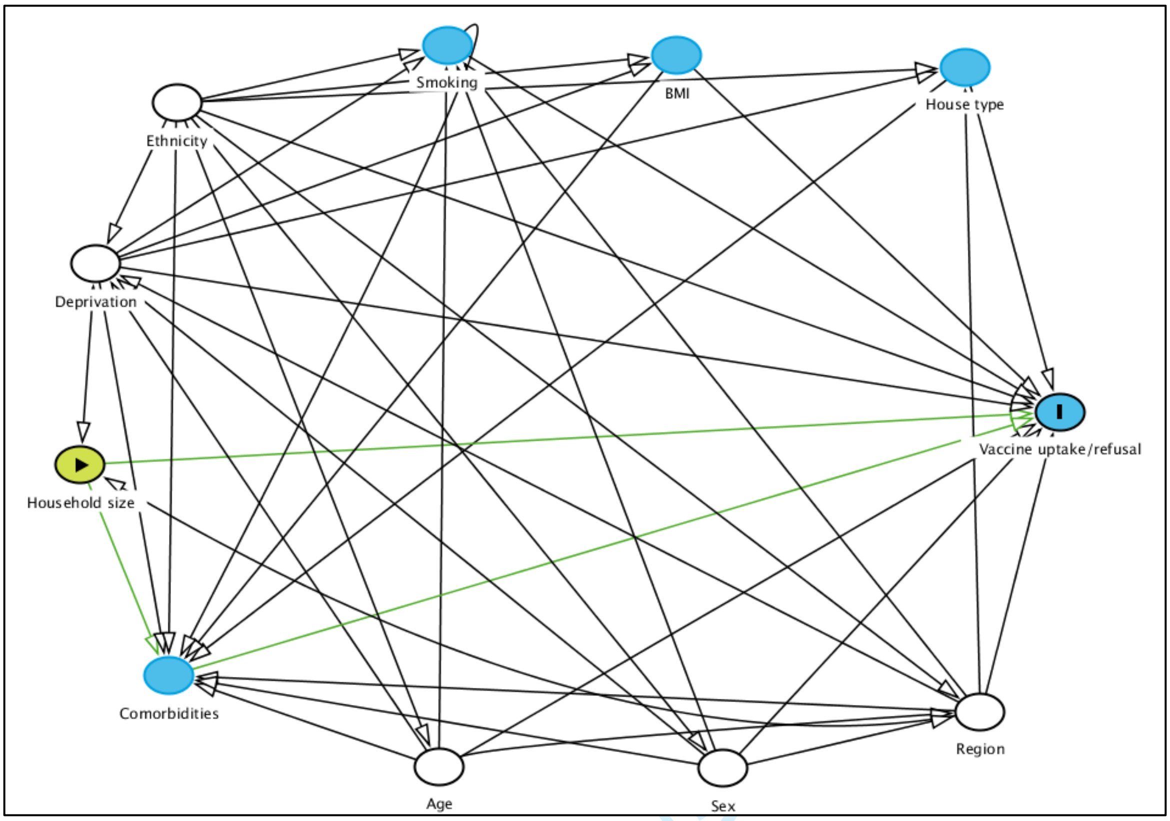

Model 4:
Exposure: Health conditions (comorbidities)
Outcome: Vaccination uptake/refusal
Confounder adjustment: age, sex, region, ethnicity, deprivation, household size, house type, smoking, BMI (identified as confounders for the association between health conditions (comorbidities) and vaccine uptake/refusal)

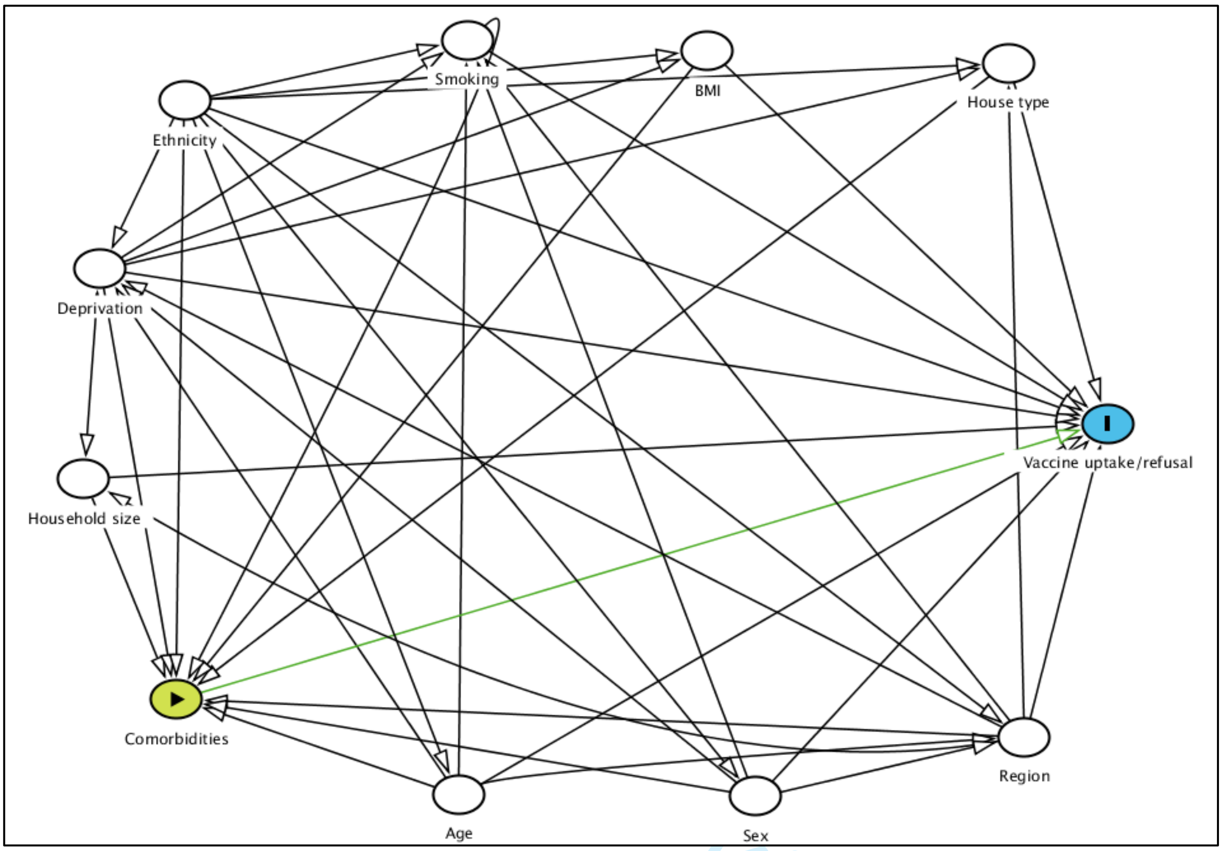

view only

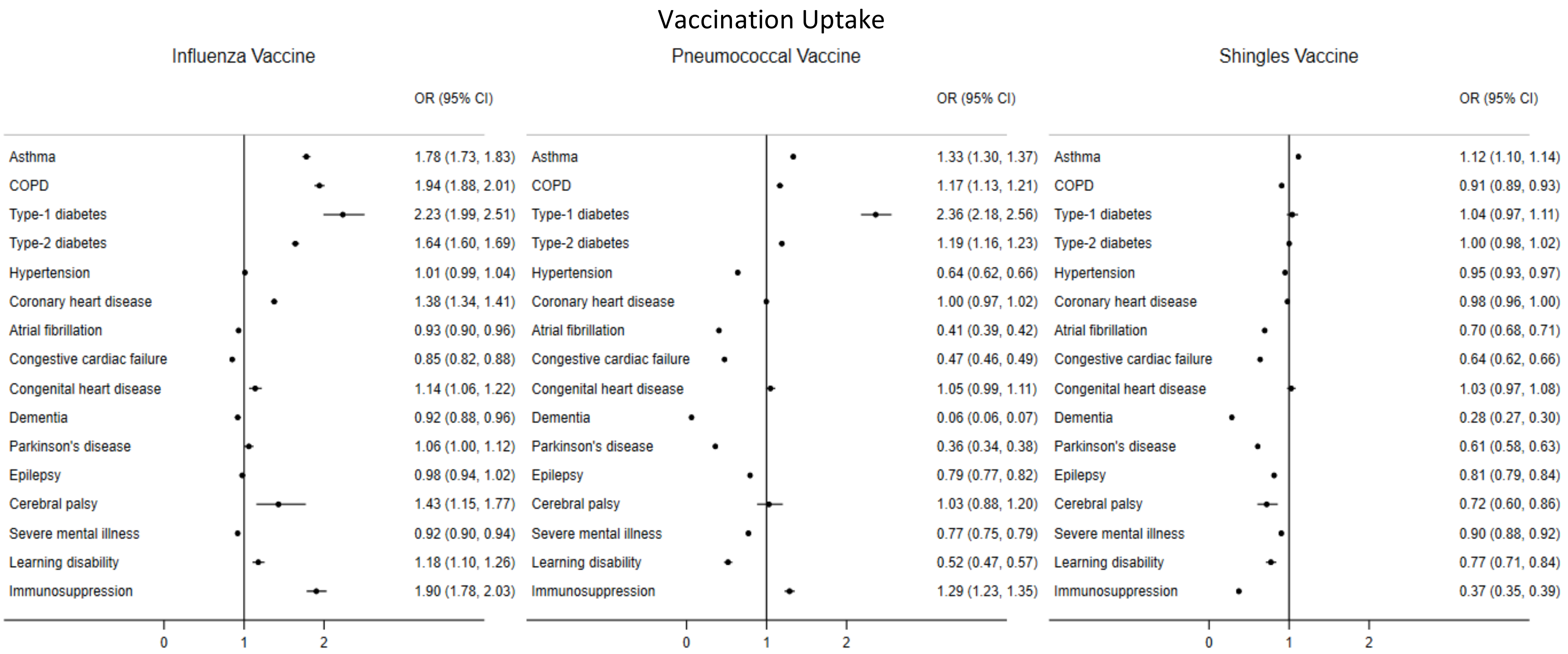

Figure S2: Associations of vaccine uptake and specific health conditions.

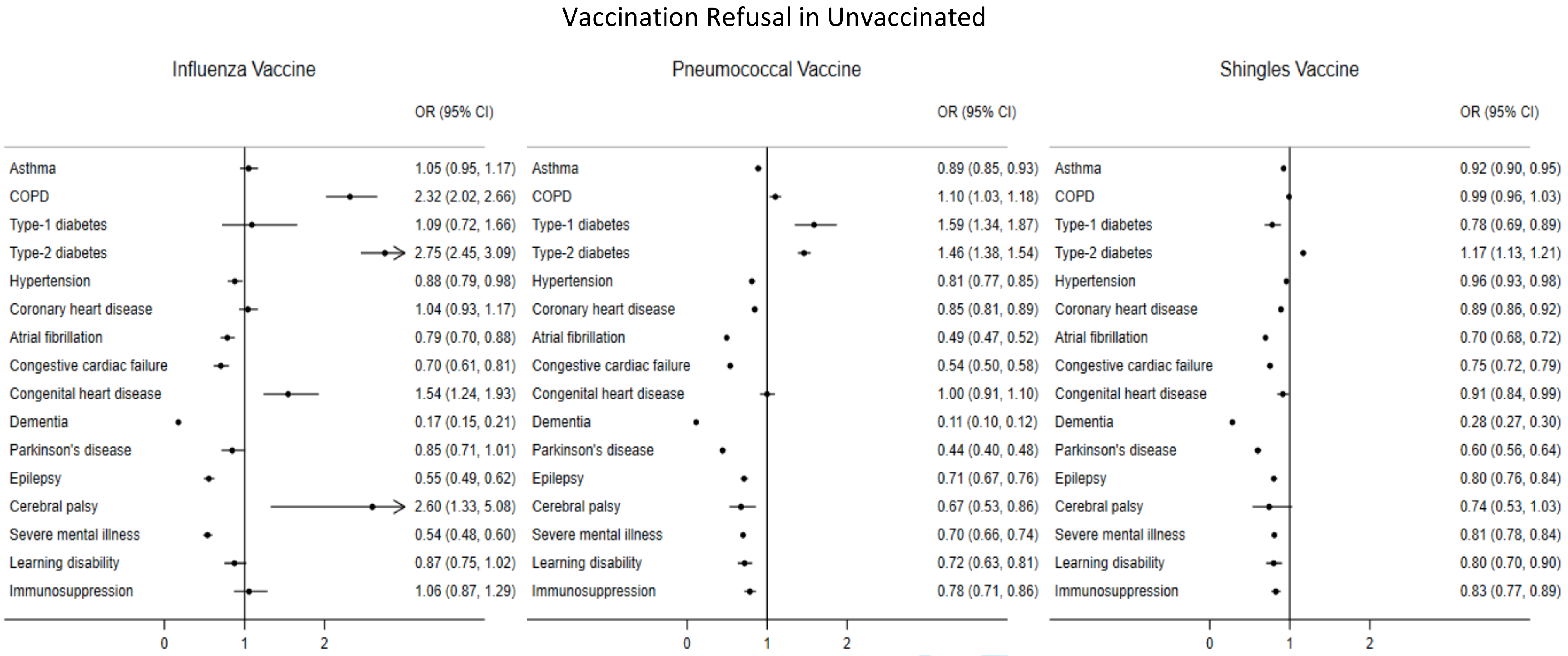

Figure S3: Associations of vaccine refusal in unvaccinated and specific health conditions.

Figure S4: Interaction analyses for vaccine uptake: ethnicity and deprivation

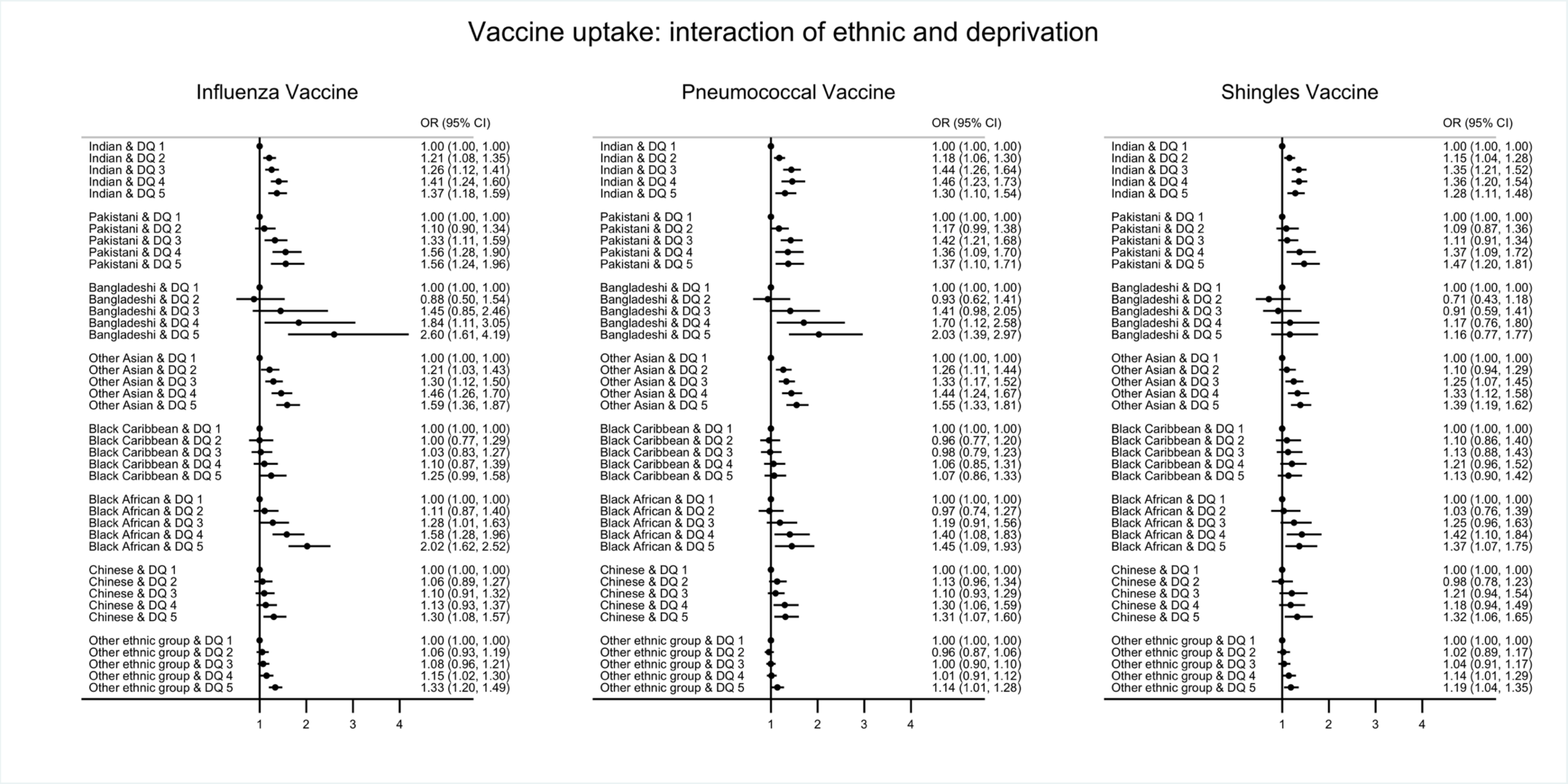

Interaction p values were <0.01 for uptake of each vaccines evaluated; DQ: deprivation quintile

Figure S5: Interaction analyses for vaccine uptake: ethnicity and household size

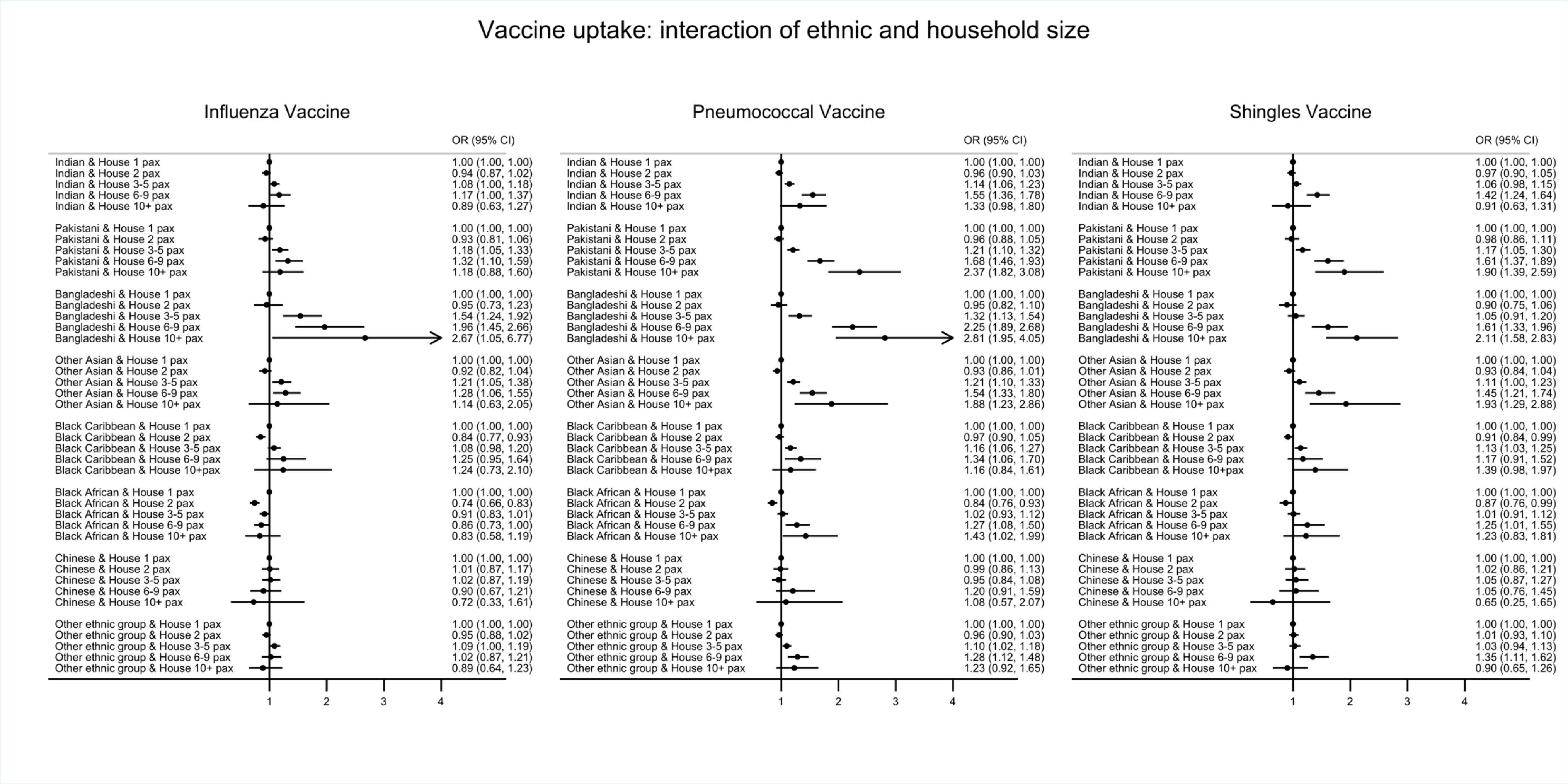

Interaction p values were <0.01 for uptake of each vaccines evaluated.

Figure S6: Interaction analyses for vaccine uptake: ethnicity and number of health conditions

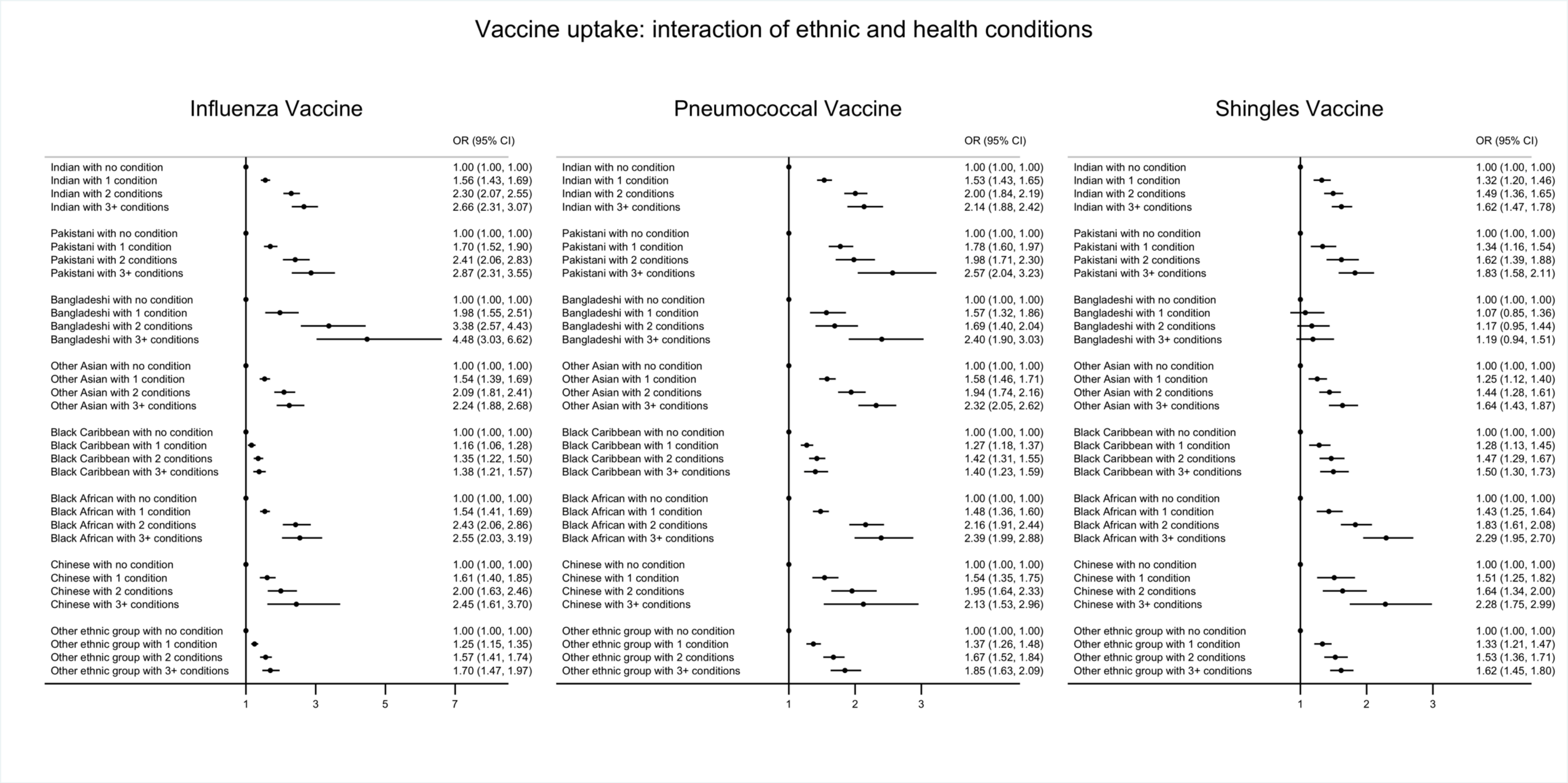

Interaction p values were <0.01 for uptake of each vaccines evaluated.

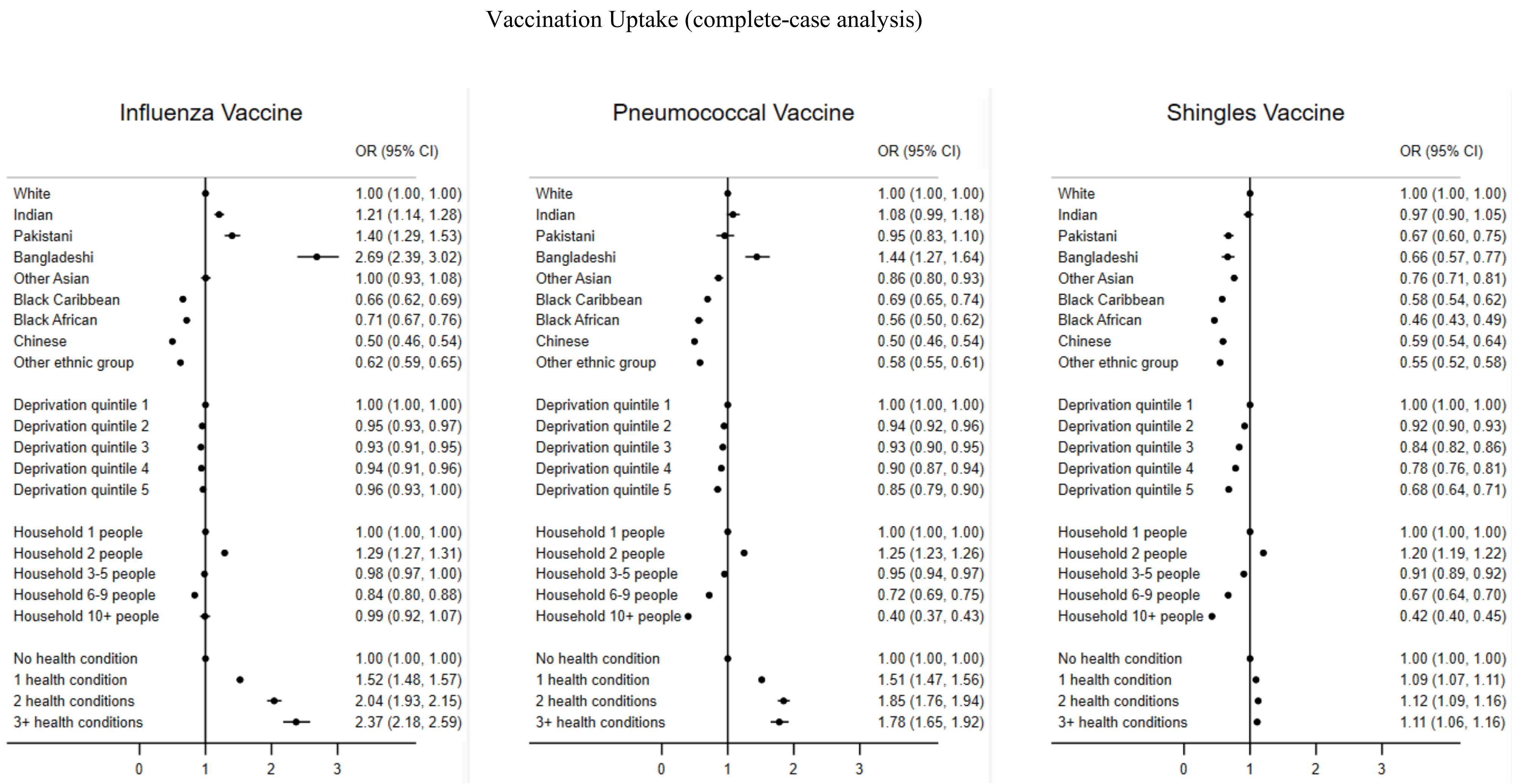

Figure S7: Sensitivity (complete-case analysis): associations of vaccine uptake and ethnic group, deprivation, household size and health conditions.

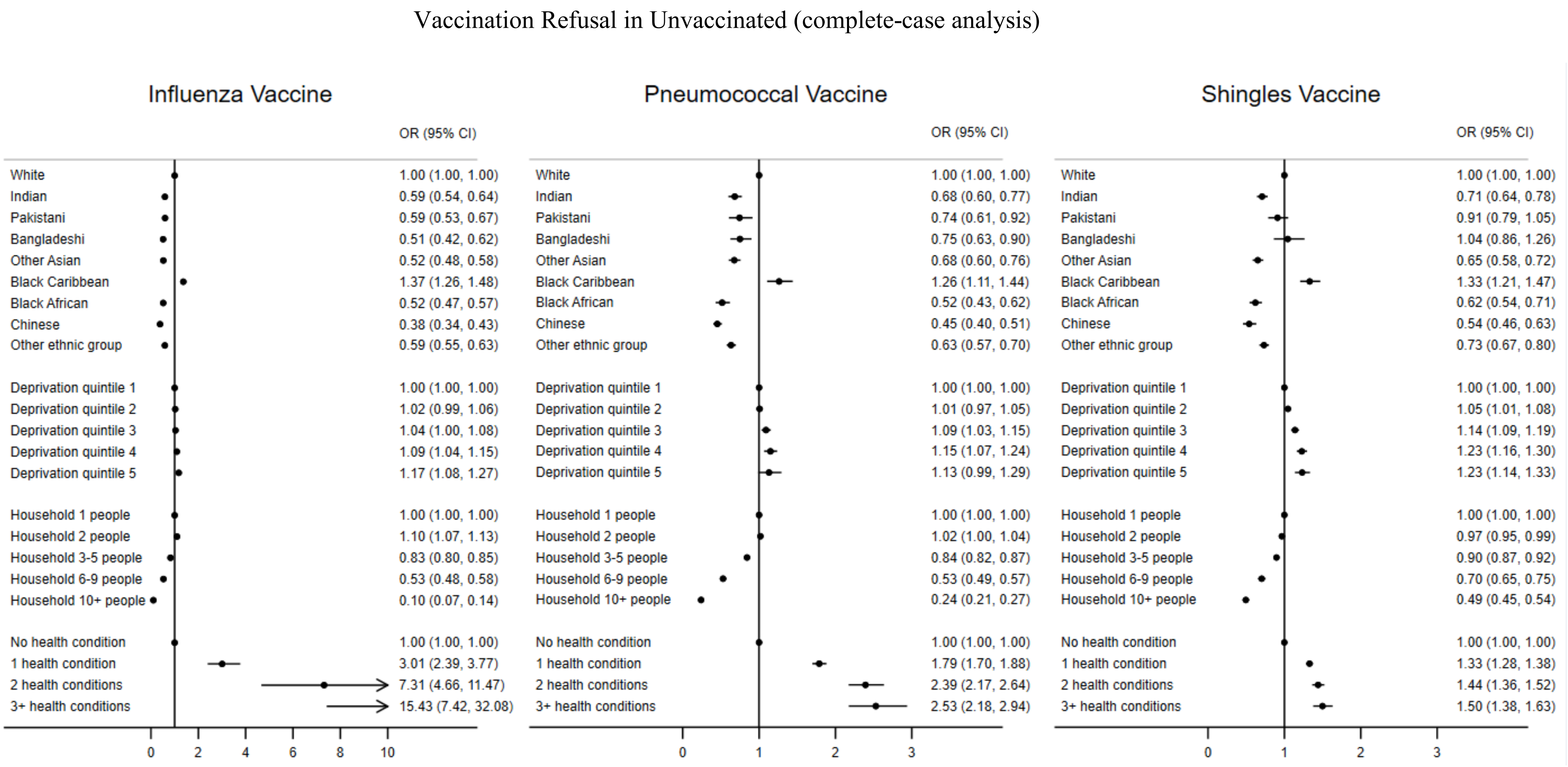

Figure S8: Sensitivity (complete-case analysis): associations of vaccine refusal (in non-vaccinated) and ethnic group, deprivation, household size and health conditions.

For peer review only

The RECORD statement – checklist of items, extended from the STROBE statement, that should be reported in observational studies using routinely collected health data.[1]

	Item No.	STROBE items	Location in manuscript where items are reported	RECORD items	Location in manuscript where items are reported
**Title and abstract**					
	1	(a) Indicate the study's design with a commonly used term in the title or the abstract (b) Provide in the abstract an informative and balanced summary of what was done and what was found	Pages 1-2	RECORD 1.1: The type of data used should be specified in the title or abstract. When possible, the name of the databases used should be included.<br><br>RECORD 1.2: If applicable, the geographic region and timeframe within which the study took place should be reported in the title or abstract.<br><br>RECORD 1.3: If linkage between databases was conducted for the study, this should be clearly stated in the title or abstract.	Page 1-2
**Introduction**					
Background rationale	2	Explain the scientific background and rationale for the investigation being reported	Page 4		
Objectives	3	State specific objectives, including any prespecified hypotheses	Page 4		
**Methods**					
Study Design	4	Present key elements of study design early in the paper	Page 5		
Setting	5	Describe the setting, locations, and relevant dates, including periods of recruitment, exposure, follow-up, and data collection	Page 5		

For peer review only

Participants	6	*(a) Cohort study* - Give the eligibility criteria, and the sources and methods of selection of participants. Describe methods of follow-up<br>*Case-control study* - Give the eligibility criteria, and the sources and methods of case ascertainment and control selection. Give the rationale for the choice of cases and controls<br>*Cross-sectional study* - Give the eligibility criteria, and the sources and methods of selection of participants<br><br>*(b) Cohort study* - For matched studies, give matching criteria and number of exposed and unexposed<br>*Case-control study* - For matched studies, give matching criteria and the number of controls per case	Page 5	RECORD 6.1: The methods of study population selection (such as codes or algorithms used to identify subjects) should be listed in detail. If this is not possible, an explanation should be provided.<br><br>RECORD 6.2: Any validation studies of the codes or algorithms used to select the population should be referenced. If validation was conducted for this study and not published elsewhere, detailed methods and results should be provided.<br><br>RECORD 6.3: If the study involved linkage of databases, consider use of a flow diagram or other graphical display to demonstrate the data linkage process, including the number of individuals with linked data at each stage.	Page 5
Variables	7	Clearly define all outcomes, exposures, predictors, potential confounders, and effect modifiers. Give diagnostic criteria, if applicable.	Pages 5-6	RECORD 7.1: A complete list of codes and algorithms used to classify exposures, outcomes, confounders, and effect modifiers should be provided. If these cannot be reported, an explanation should be provided.	Page 5-6
Data sources/ measurement	8	For each variable of interest, give sources of data and details of methods of assessment (measurement).<br>Describe comparability of assessment methods if there is more than one group	Pages 5-6		

For peer review only

Bias	9	Describe any efforts to address potential sources of bias	Page 6		
Study size	10	Explain how the study size was arrived at	Pages 5-6		
Quantitative variables	11	Explain how quantitative variables were handled in the analyses. If applicable, describe which groupings were chosen, and why	Pages 5-6		
Statistical methods	12	(a) Describe all statistical methods, including those used to control for confounding<br>(b) Describe any methods used to examine subgroups and interactions<br>(c) Explain how missing data were addressed<br>(d) *Cohort study* - If applicable, explain how loss to follow-up was addressed<br>*Case-control study* - If applicable, explain how matching of cases and controls was addressed<br>*Cross-sectional study* - If applicable, describe analytical methods taking account of sampling strategy<br>(e) Describe any sensitivity analyses	Page 6-7		
Data access and cleaning methods		..		RECORD 12.1: Authors should describe the extent to which the investigators had access to the database population used to create the study population.<br><br>RECORD 12.2: Authors should provide information on the data	Pages 5-6

For peer review only

				cleaning methods used in the study.	
Linkage		..		RECORD 12.3: State whether the study included person-level, institutional-level, or other data linkage across two or more databases. The methods of linkage and methods of linkage quality evaluation should be provided.	-
**Results**					
Participants	13	(a) Report the numbers of individuals at each stage of the study (*e.g.*, numbers potentially eligible, examined for eligibility, confirmed eligible, included in the study, completing follow-up, and analysed)<br>(b) Give reasons for non-participation at each stage.<br>(c) Consider use of a flow diagram	Page 8	RECORD 13.1: Describe in detail the selection of the persons included in the study (*i.e.,* study population selection) including filtering based on data quality, data availability and linkage. The selection of included persons can be described in the text and/or by means of the study flow diagram.	Page 8
Descriptive data	14	(a) Give characteristics of study participants (*e.g.*, demographic, clinical, social) and information on exposures and potential confounders<br>(b) Indicate the number of participants with missing data for each variable of interest<br>(c) *Cohort study* - summarise follow-up time (*e.g.*, average and total amount)	Page 8		
Outcome data	15	*Cohort study* - Report numbers of outcome events or summary measures over time<br>*Case-control study* - Report numbers in each exposure	Page 8		

For peer review only

		category, or summary measures of exposure<br>*Cross-sectional study* - Report numbers of outcome events or summary measures			
Main results	16	(a) Give unadjusted estimates and, if applicable, confounder-adjusted estimates and their precision (e.g., 95% confidence interval). Make clear which confounders were adjusted for and why they were included<br>(b) Report category boundaries when continuous variables were categorized<br>(c) If relevant, consider translating estimates of relative risk into absolute risk for a meaningful time period	Pages 8-9		
Other analyses	17	Report other analyses done—e.g., analyses of subgroups and interactions, and sensitivity analyses	Page 10		
**Discussion**					
Key results	18	Summarise key results with reference to study objectives	Page 11		
Limitations	19	Discuss limitations of the study, taking into account sources of potential bias or imprecision. Discuss both direction and magnitude of any potential bias	Page 12-13	RECORD 19.1: Discuss the implications of using data that were not created or collected to answer the specific research question(s). Include discussion of misclassification bias, unmeasured confounding, missing data, and changing eligibility over time, as they pertain to the study being reported.	Page 12-13
Interpretation	20	Give a cautious overall interpretation of results	Pages 11-13		

For peer review only

		considering objectives, limitations, multiplicity of analyses, results from similar studies, and other relevant evidence			
Generalisability	21	Discuss the generalisability (external validity) of the study results	Pages 13		
**Other Information**					
Funding	22	Give the source of funding and the role of the funders for the present study and, if applicable, for the original study on which the present article is based	Page 14-15		
Accessibility of protocol, raw data, and programming code		..	Page 15	RECORD 22.1: Authors should provide information on how to access any supplemental information such as the study protocol, raw data, or programming code.	Page 15

*Reference: Benchimol EI, Smeeth L, Guttmann A, Harron K, Moher D, Petersen I, Sørensen HT, von Elm E, Langan SM, the RECORD Working Committee. The REporting of studies Conducted using Observational Routinely-collected health Data (RECORD) Statement. *PLoS Medicine* 2015; in press.

*Checklist is protected under Creative Commons Attribution (CC BY) license.

For peer review only

BMJ Open

Factors influencing influenza, pneumococcal and shingles vaccine uptake and refusal in older adults: a population-based cross-sectional study in England

Journal:	*BMJ Open*
Manuscript ID	bmjopen-2021-058705.R2
Article Type:	Original research
Date Submitted by the Author:	27-Jun-2022
Complete List of Authors:	Tan, Pui San; University of Oxford, Nuffield Department of Primary Care Health Sciences<br>Patone , Martina ; University of Oxford<br>Clift, Ashley; University of Oxford, Nuffield Department of Primary Care Health Sciences<br>Dambha-Miller, Hajira; University of Southampton Faculty of Medicine, Primary Care Research Centre<br>Saatci, Defne; University of Oxford, Nuffield Department of Primary Care Health Science<br>Ranger, Tom; Oxford University, Primary Care Health Sciences<br>Garriga, Cesar; University of Oxford, Nuffield Department of Primary Care Health Sciences<br>Zaccardi, Francesco; University of Leicester; University of Leicester, Diabetes Research Centre<br>Shah, Baiju; Sunnybrooke Health Sciences Centre<br>Coupland, Carol; University of Nottingham, Division of Primary Care<br>Griffin, Simon; The Primary Care Unit<br>Khunti, Kamlesh; University of Leicester, Diabetes Research Centre<br>Hippisley-Cox, Julia; University of Oxford, Nuffield Department of Primary Care Sciences
**Primary Subject Heading**:	Public health
Secondary Subject Heading:	Epidemiology, Geriatric medicine, Infectious diseases
Keywords:	EPIDEMIOLOGY, Epidemiology < INFECTIOUS DISEASES, Public health < INFECTIOUS DISEASES, PRIMARY CARE

SCHOLARONE™
Manuscripts

I, the Submitting Author has the right to grant and does grant on behalf of all authors of the Work (as defined in the below author licence), an exclusive licence and/or a non-exclusive licence for contributions from authors who are: i) UK Crown employees; ii) where BMJ has agreed a CC-BY licence shall apply, and/or iii) in accordance with the terms applicable for US Federal Government officers or employees acting as part of their official duties; on a worldwide, perpetual, irrevocable, royalty-free basis to BMJ Publishing Group Ltd ("BMJ") its licensees and where the relevant Journal is co-owned by BMJ to the co-owners of the Journal, to publish the Work in this journal and any other BMJ products and to exploit all rights, as set out in our licence.

The Submitting Author accepts and understands that any supply made under these terms is made by BMJ to the Submitting Author unless you are acting as an employee on behalf of your employer or a postgraduate student of an affiliated institution which is paying any applicable article publishing charge ("APC") for Open Access articles. Where the Submitting Author wishes to make the Work available on an Open Access basis (and intends to pay the relevant APC), the terms of reuse of such Open Access shall be governed by a Creative Commons licence – details of these licences and which Creative Commons licence will apply to this Work are set out in our licence referred to above.

Other than as permitted in any relevant BMJ Author's Self Archiving Policies, I confirm this Work has not been accepted for publication elsewhere, is not being considered for publication elsewhere and does not duplicate material already published. I confirm all authors consent to publication of this Work and authorise the granting of this licence.

Factors influencing influenza, pneumococcal and shingles vaccine uptake and refusal in older adults: a population-based cross-sectional study in England

Pui San Tan[1*], Martina Patone[1*], Ash Kieran Clift[1,2*], Hajira Dambha-Miller[3], Defne Saatci[1], Tom A. Ranger[1], Cesar Garriga[1], Francesco Zaccardi[4], Baiju R. Shah[5], Carol Coupland[6], Simon J. Griffin[7,8], Kamlesh Khunti[4] & Julia Hippisley-Cox[1]

1: Nuffield Department of Primary Care Health Sciences, University of Oxford

2: Cancer Research UK Oxford Centre, Department of Oncology, University of Oxford

3: Primary Care Research Centre, University of Southampton

4: Leicester Diabetes Centre, University of Leicester

5: Department of Medicine, University of Toronto

6: Division of Primary Care, School of Medicine, University of Nottingham

7: Department of Public Health and Primary Care, School of Clinical Medicine, University of Cambridge

8: MRC Epidemiology Unit, School of Clinical Medicine, University of Cambridge

Corresponding author:

Dr Pui San Tan

Nuffield Department of Primary Care Health Sciences, University of Oxford

Radcliffe Primary Care Building, Radcliffe Observatory Quarter, Woodstock Rd,

Oxford OX2 6GG, UK

pui.tan@phc.ox.ac.uk

*Authors contributed equally to this work

For peer review only - http://bmjopen.bmj.com/site/about/guidelines.xhtml

Abstract:

Objectives: Uptake of influenza, pneumococcal and shingles vaccines in older adults remain varied across different regions and socioeconomic backgrounds. In this study, we studied the coverage and factors associated with vaccination uptake, as well as refusal in the unvaccinated population and their associations with ethnicity, deprivation, household size, and health conditions.

Design, setting and participants: This is a cross-sectional study of adults aged 65 years or older in England, using a large primary-care database. Associations of vaccine uptake and refusal in the unvaccinated with ethnicity, deprivation, household size, and health conditions were modelled using multivariable logistic regression.

Outcome measure: Influenza, pneumococcal and shingles vaccine uptake and refusal (in the unvaccinated).

Results: This study included 2,054,463 patients from 1,318 general practices. 1,711,465 (83.3%) received at least one influenza vaccine, 1,391,228 (67.7%) pneumococcal vaccine, and 690,783 (53.4%) shingles vaccine. Compared to White ethnicity, influenza vaccine uptake was lower in Chinese (odds ratio (OR) 0.49; 95% CI: 0.45-0.53), 'Other ethnic' groups (0.63; 0.60-0.65), Black Caribbean (0.68; 0.64-0.71), and Black African (0.72; 0.68 -0.77). There was generally lower vaccination uptake among more deprived individuals, people living in larger household sizes (3 or more persons) and those with lesser health conditions. Among those who were unvaccinated, higher odds of refusal was associated with the Black Caribbean ethnic group and marginally with more deprived individuals, but was not associated with higher refusal in those living in large households or those with lesser health conditions.

Conclusion: Certain ethnic minority, deprived populations, large households and healthier individuals were less likely to receive a vaccine, although higher refusal was only associated with ethnicity and deprivation but not larger households nor healthier individuals. Understanding these may inform tailored public health messaging to different communities for equitable implementation of vaccination programs.

Keywords: vaccine, uptake, refusal, ethnicity, deprivation, equality

For peer review only

Strengths and limitations of the study

- Use of a large primary care database offered a population-representative population in terms of demographics including ethnic groups and deprivation

- Using a primary care database captures comprehensive vaccination data, including those occurring outside general practice (such as in pharmacies), as well as recorded invitations to vaccination sent by general practices and patient refusals

- There was lack of recording of variables such as personal beliefs, literacy levels, language barriers, access and education status, and hence we were unable to evaluate the impact of these factors on vaccination uptake and refusal

- Classification of vaccination-related endpoints was reliant on individual practitioners; however, we used an appropriately wide range of codes in our endpoint definitions

For peer review only

For peer review only - http://bmjopen.bmj.com/site/about/guidelines.xhtml

Background

Older adults are often more susceptible to infectious diseases circulating in the community, and may develop more severe health outcomes when infected due to lower immune responses associated with aging[1] and comorbidities. National influenza, pneumococcal, and shingles vaccination programs for older adults have been implemented in the UK in various phases.[2-4] Through these national vaccination programmes, 'seasonal' influenza vaccines are offered annually, pneumococcal vaccines are offered as a single dose to adults aged 65 years and above, whilst the shingles vaccine is offered as a single dose to adults aged 70-79 years.[2-4]

The World Health Organisation (WHO) recommends a target of 75% population vaccination coverage.[5] Recent reports from Public Health England (PHE) have reported 81% influenza vaccination coverage and 69% pneumococcal vaccination coverage in adults aged 65 years and above, and 47% to 77% for shingles vaccination coverage in adults aged 71 and 78, respectively.[2-4] However, some evidence suggests that there could be vast differences in terms of vaccination coverage, potentially varying by geographical region, ethnicity, deprivation, household size, and health conditions.[2-4, 6, 7]

For the purposes of equitable public health strategy, it is important to understand factors associated with uptake of vaccinations, and refusal of vaccinations in the unvaccinated population. Prior studies have demonstrated differential uptake of existing vaccinations across sociodemographic groups, however, many studies have either studied single vaccinations, not captured the appreciable case-mix inherent to sociodemographic groups (such as by using broad ethnic categories), analysed a small set of relevant health conditions, and relied on potentially imprecise or biased self-report measures.[7-9] In addition, although household size is known to increase the risk of transmission for infectious diseases, evidence on the association between household size and vaccination uptake remains limited.[10] A few previous studies have suggested that individuals from larger households were less likely to be vaccinated, although these studies were small and mainly focused on childhood vaccinations.[11, 12] Further, it is of interest to understand the pathway events leading to the lack of vaccine uptake, and to what extent these are driven by patient refusal.

Here, we evaluated factors associated with uptake and refusal of existing national vaccination programs (influenza, pneumococcal and shingles) in older adults (aged 65 years and above) in England and their

For peer review only

associations with ethnic group, deprivation, household size, and health conditions.

For peer review only

For peer review only - http://bmjopen.bmj.com/site/about/guidelines.xhtml

Methods

Study population and data source

We performed a population-based cross-sectional study using QResearch (version 45). QResearch is a database with over 10 million current patients registered at more than 1800 practices in England. QResearch is an electronic healthcare primary care database in the UK with individual patient level records for general practices (GP) using the EMIS computer record system. The database captures information from GP consultations; among which includes patient demographics, socioeconomic status, diagnoses, laboratory test results, treatments and vaccinations. The database has good representation of the general population of England, particularly in terms of different ethnic groups with proportions close to those reported by Office for National Statistics.[13]

In this study, we included adults aged 65-99 years currently registered with 1,318 practices during the period 24th January 2020 to 31st October 2020, which comprised 2,054,463 of approximately 13.7 million patients aged 65 and over registered with a GP in England.[14] We assessed the uptake and refusal of influenza, pneumococcal, and shingles vaccines from 1st January 1989 until 31st October 2020 (last database update) as our main study outcome. As the shingles vaccination was rolled out nationally in England in 2013 for those aged 70 and up until 79 ,[15] we included in our shingles vaccine analysis only those aged 70 and above, excluding those aged 80 and above in year 2013 as they were not eligible at the time. Uptake was defined as the last recorded instance of receiving the vaccines of interest within the study period. This was mostly in GP surgeries (~99%), but also in-hospital or pharmacy administrations. Refusal was analysed in those with no record of vaccination, defined as last recorded instances of explicit refusal (74-82% of recorded code instances), consent not being given (18-26%), or non-attendance to a scheduled vaccination appointment (0.03-0.3%).[16] Outcomes were defined using code dictionaries comprising relevant Read and SNOMED codes as inputted into the EMIS software by healthcare practitioners.

We extracted demographic data including age, sex, self-reported ethnic group, Townsend deprivation index quintile,[17, 18] geographical region within England (n=10, see Table 1), housing status and household size. Townsend deprivation score is an index of deprivation commonly used in the UK to measure socioeconomic status. It uses the following characteristics to measure deprivation by postcode; proportion of (1) unemployment, (2) non-car ownership, (3) non-home ownership, and (4) household crowding – with a higher score suggests greater deprivation. In this study the scores were reported in quintiles, i.e. first quintile indicates the least deprived. while fifth quintile indicates most deprived.

For peer review only - http://bmjopen.bmj.com/site/about/guidelines.xhtml

Ethnicity was grouped into 9 categories – White (White British, White Irish, Other White), Indian, Pakistani, Bangladeshi, Other Asian, Black Caribbean, Black African, Chinese, Other ethnic group (White & Black, White & Asian, Other mixed, Other Black, Other ethnic group). We also extracted data using GP Read and SNOMED codes from primary care records and ICD-10 codes from hospital records (where available) for diagnoses of asthma, chronic obstructive pulmonary disease (COPD), diabetes mellitus (type 1 and 2), hypertension, coronary heart disease (CHD), atrial fibrillation (AF), congenital heart disease, congestive cardiac failure (CCF), chronic neurological diseases (Parkinson's disease, epilepsy, cerebral palsy), learning disability, dementia, and severe mental illness (schizophrenia, severe depression, bipolar affective disorder and psychosis) and immune suppression (based on use of immunosuppressant medications). For each vaccination outcome (uptake and refusal), people with health conditions diagnosed prior to the vaccination outcome were defined as exposed, while those diagnosed with health conditions after the outcome were defined as unexposed. The most recently recorded BMI and smoking status were identified for each individual.

Analyses

Descriptive analyses compared the uptake and refusal of the three vaccinations of interest by ethnic group, Townsend deprivation quintiles, household size and individual health conditions. Percentage uptake of each vaccination in individual general practices was plotted to display between-region variations.

Multivariable logistic regression models examined associations between ethnic group, deprivation, household size, health conditions and vaccination uptake and refusal by calculating adjusted odds ratios (OR) and their 95% confidence intervals (CI). Clustered robust standard errors were used to account for clustering of individuals within general practices. Refusals were evaluated in never-receivers of each vaccine (no uptake). Individual models for each exposure (ethnic group, deprivation, household size, health conditions) and outcome (vaccination uptake and refusal for each vaccine) were fitted separately, allowing for adjustment of confounders: age, sex, geographical region, type of home, smoking status and/or BMI as relevant according to directed acyclic graphs (DAGs) - (i) Ethnicity – no adjustments; (ii) Deprivation - adjusted for age, sex, region, ethnicity, household size; (iii) Household size – adjusted for age, sex, region, ethnicity, deprivation, (iv) Health conditions - age, sex, region, ethnicity, deprivation, household size, house type, smoking and BMI. (Figure S1).

For peer review only - http://bmjopen.bmj.com/site/about/guidelines.xhtml

Missing data for ethnic group (18.5%), BMI (5.6%), deprivation quintiles (0.3%) and smoking status (1.0%) were multiply imputed using chained equations under the missing at random assumption. Five imputations were generated using a single rich imputation model incorporating all outcomes, exposures and confounder covariates. Models were fitted in each of the 5 imputed datasets with model coefficients and their standard errors pooled in accordance with Rubin's rules.[19] We also performed sensitivity analyses of results using complete-case analysis.

In addition, we performed post-hoc interaction analyses to explore potential interactive effects for vaccine uptake between ethnicity and deprivation, household size, and number of health conditions.

RECORD guidelines were used for reporting.[20] Statistical analyses were performed using STATA v17.0.[21]

Patient and public involvement reporting

Two public representatives advised on interest and appropriateness of the research questions, were involved in writing the protocol for the wider study, and input on lay-summaries describing the planned study.

For peer review only - http://bmjopen.bmj.com/site/about/guidelines.xhtml

Results

This study included 2,054,463 patients aged 65 years and older registered with 1,318 general practices. Characteristics of the study population are shown in Table 1 and S1. At least one influenza vaccine was received by 1,711,465 (83.3) patients, a pneumococcal vaccine by 1,391,228 (67.7%), and a shingles vaccine by 690,783 (53.4% of over 70s). Figure 1 showed a descriptive overview of the rate of vaccination uptake and refusals by different regions in England at the practice level. for example, the median level of shingles vaccine uptake in London practices was ~50%, compared to ~60% in East England. Overall, uptake of influenza vaccine (~80%) was the highest among all three vaccine types, followed by pneumococcal vaccine (~70%) and shingles vaccine (~50%) (Figure 1).

Vaccination uptake

Vaccination uptake differed by ethnicity, deprivation, household size, and health conditions (Figure 1). In multivariable analysis compared to the White population, those from Black Caribbean, Black African, Chinese and Other ethnic groups showed lower uptake for all three vaccines (Figure 2). Influenza vaccination uptake was significantly lower in Black Caribbean (OR 0.68, 95% CI: 0.64-0.71), Black African (OR 0.72; 0.68-0.77), Chinese (OR 0.49; 0.45-0.53) and 'Other ethnic group' (OR 0.63; 0.60-0.65), but there was significantly higher uptake in Indian (OR 1.21; 1.14-1.28), Pakistani (OR 1.39; 1.28-1.52), and Bangladeshi (OR 2.68; 2.38-3.01) ethnic groups compared to the White group.

There was a similar pattern observed for pneumococcal vaccination uptake: Black Caribbean (OR 0.70; 0.66-0.75), Black African (OR 0.56; 0.51-0.62), Chinese (OR 0.49; 0.45-0.53), 'Other ethnic group' (OR 0.58; 0.55-0.61), and also additionally for Other Asian (OR 0.87; 0.80-0.93). Pneumococcal vaccine uptake was significantly higher only in Bangladeshi ethnic group (OR 1.46; 1.29-1.65) compared to the White group. For shingles vaccine uptake, there was significantly lower uptake in all ethnic minority groups except in Indians (OR 0.98; 0.91-1.05).

For all three vaccines, vaccine uptake was generally lower among the more deprived, with the most deprived (lowest quintile) having 6% to 33% lower odds of vaccine uptake (ORs 0.67 to 0.94) compared to the most affluent. People in households with two people had 22% to 32% higher odds of having a vaccine compared to one-person households. However, the odds were lower in household sizes above

For peer review only - http://bmjopen.bmj.com/site/about/guidelines.xhtml

three, with people in households of 10 or more people having 17% to 63% lower odds to have vaccine uptake compared to one-person households.

The uptake of each vaccination was also generally associated with increasing number of health conditions; with asthma being associated with higher uptake of all three vaccines, while atrial fibrillation, congestive cardiac failure, dementia, severe mental illness were being associated with lower uptake of all three vaccines. Individuals with COPD, diabetes and immunosuppression were also more likely to be associated with higher uptake of both influenza and pneumococcal vaccines but not for shingles vaccine (Figure S2).

Vaccination refusals in the unvaccinated

There were consistently significantly higher odds of vaccine refusal amongst the Black Caribbean group compared to the White group for all three vaccines; influenza (OR 1.45; 1.34-1.56), pneumococcal (OR 1.29; 1.14-1.46) and shingles (OR 1.35; 1.23-1.49). Indian, Pakistani, Bangladeshi, Other Asian, Black African, Chinese, and Other ethnic groups were significantly less likely to refuse all three vaccines compared to White ethnic group, except for Pakistani and Bangladeshi, which showed no significant association with shingles vaccine refusal. (Figure 3)

There was a general trend of refusal with increasing deprivation, particularly with shingles vaccine in the two most deprived quintiles, OR 1.21; 1.15-1.28, and OR 1.23; 1.14-1.33 (4th and 5th deprivation quintiles, respectively). Higher household size was associated with lower odds of refusal of all three vaccines in households of 3+ people and more. (Figure 3)

In individuals with three or more health conditions, the odds of refusal were: influenza vaccine (OR 10.29; 7.38-14.37), pneumococcal vaccine (OR 2.55; 2.24-2.90), shingles vaccine (1.60; 1.48-1.73). Individuals with type 2 diabetes consistently showed higher vaccine refusal for all three vaccines and individuals with COPD was also associated with higher refusal for influenza and pneumococcal vaccines. (Figure S3)

For peer review only - http://bmjopen.bmj.com/site/about/guidelines.xhtml

For peer review only

Additional analyses

Further, we explored the interactions for vaccine uptake between ethnicity and deprivation, house size and number of health conditions. First, results suggested that certain ethnic minority groups who were more deprived could be more likely to receive a vaccine, particularly Bangladeshi and Black African. (Figure S4) Second, across all three vaccines evaluated, Bangladeshi living in larger households could be more likely to receive a vaccine (Figure S5) Third, vaccine uptake was generally more likely in individuals with higher number of health conditions, although the magnitude of effect varied slightly across different ethnic groups. (Figure S6)

Finally, we performed sensitivity analyses to evaluate associations of vaccine uptake and refusal using complete-case analyses. In this analysis we excluded individuals with missing information on covariates i.e. ethnicity, deprivation, BMI and smoking. Results on Figure S7-8 showed that estimates were comparable with multiply imputed analysis presented as our main findings above.

For peer review only

For peer review only - http://bmjopen.bmj.com/site/about/guidelines.xhtml

Discussion

Summary

In this study, we observed generally lower uptake of influenza, pneumococcal and shingles vaccinations in particular ethnic minority groups and deprived populations. Black Caribbean, Black African, Chinese and Other ethnic groups consistently showed lower uptake of all three vaccines studied compared to the White ethnic group. In the unvaccinated population, the Black Caribbean ethnic group consistently showed lower vaccine uptake and increased odds of vaccine refusal for all three vaccines. More deprived populations also showed lower vaccine uptake with higher refusals in the unvaccinated. Household sizes above 3 persons were associated with lower vaccine uptake, but were not associated with higher refusal. Further, a lower number of pre-existing health conditions was generally associated with lower odds of vaccine uptake, although this was not reflected in terms of higher odds of refusal.

Comparison with existing literature

Our observations that influenza vaccination uptake is inversely correlated with deprivation and varies across ethnic groups build upon results from a recent study of adults between 2011-2016 using the CPRD database.[7] This study analysed seasonal influenza vaccination uptake across 5 'seasons' and similarly found that in the over 65s, Black individuals were significantly less likely than White individuals to receive this vaccination. However, our study finds that South Asians may be more likely to have higher uptake of influenza vaccine, which may warrant further qualitative study to examine potential socioeconomic and behavioural factors driving this observation. Our examination of three vaccinations within a larger sample size (over 2 million vs. 611,000), a more granular categorisation of ethnic groups (9 vs. 4) and regions (10 vs. 4), improved handling of missing data, and our analysis of vaccination refusals in the unvaccinated substantially improves our understanding of these complex public health behaviours. Our results showed that although four ethnic minority groups (Black Caribbean, Black African, Chinese and Other ethnic group) had lower uptake of influenza vaccine, only the Black Caribbean group showed increased odds of refusal among the unvaccinated.

We also found lower vaccine uptake in household sizes above 3 persons, although they also showed lower refusals in the unvaccinated population. This suggests that lower vaccine uptake in larger households could be driven by barriers to vaccine uptake other than due to refusal alone. A study in Hong Kong showed that vaccine uptake in the elderly living with younger family members had lower

For peer review only - http://bmjopen.bmj.com/site/about/guidelines.xhtml

vaccine uptake compared to elderly living alone or living with other elderly household members.[6] This calls for further ethnographic research to explore social and household characteristics including age structure of household members and its potential association with vaccine uptake in the elderly in England.

Higher uptake of influenza and pneumococcal vaccinations in individuals with asthma, COPD, diabetes and immunosuppression could be related to clinical guidelines where individuals in these clinical risk groups would be more likely to be offered a vaccine by their health care providers.[22, 23] On the contrary, lower vaccine uptake in those with fewer health conditions could potentially be attributable to reduced contact with health services in the healthier population and hence, reduced likelihood to receive 'opportunistic' vaccination offers. Despite that, it is worth noting that our study also found that in the unvaccinated population there remains significant refusal in those with type-2 diabetes and COPD. Possibly relevant factors could be resistance to lifestyle and behaviour changes, in which individuals with diabetes and COPD who might be more likely to have unhealthy lifestyles e.g. smoking[24, 25] might also be less receptive to health interventions i.e. vaccines. However, this finding needs confirmation in other studies. In addition, interaction analyses from our study showed that certain ethnic minority groups i.e. Bangladeshis who were more deprived and living in larger households were more likely to receive a vaccine. This could potentially be due to availability of outreach programs organised by local communities and GPs in these areas to create awareness and provide health education.[26, 27]

Vaccine hesitancy findings from this study may also be relevant to ongoing COVID-19 vaccine hesitancy in the population. In a population study in older adults using National Immunisation Management System (NIMS) in the England, UK, it has been similarly shown that ethnic minority Black African and Black Caribbean and more deprived populations were less likely to receive COVID-19 vaccine.[28] These similarities in findings across different vaccines suggest possible shared drivers of vaccine hesitancy, which might help inform future public health strategies for equitable implementation of vaccination programs in general.

For peer review only - http://bmjopen.bmj.com/site/about/guidelines.xhtml

Strengths and limitations

Use of the QResearch database offered a population-representative population with accurately coded data, enabling capture of vaccinations occurring outside general practice (such as in pharmacies), as well as recorded invitations to vaccination sent by general practices and patient refusals. This permitted a robust evaluation of not only uptake, but also possible contributory mechanisms leading to uptake behaviours. Limitations include the lack of recording of variables such as religion, personal beliefs and reasons for refusal that predicate vaccine hesitancy in our sample. Further, our dataset also did not capture literacy levels, language barriers, access and education status, and hence were not able to evaluate the impact of these socioeconomic factors on vaccination uptake and refusal. These could be important factors influencing the complex decision-making and behavioural aspects and hence would warrant further qualitative and ethnography studies. Classification of vaccination-related endpoints was reliant on individual practitioners using Read and SNOMED codes on the EMIS software system; however, as GP surgeries are financially incentivised through 'Quality Outcome Framework' payments to record vaccination services and we used an appropriately wide range of codes in our endpoint definitions, the risk of misclassification may be low.

Implications for research and practice

Two key principles in health inequalities are Tudor-Hart's inverse care law,[29] where service provision is inversely proportional to the need for it, and the inverse equity hypothesis, which posits that new healthcare interventions are most likely to be taken up by those in less need and thus exacerbate pre-existing inequality in the short term. Our study may help inform policymakers regarding reducing inequity in the uptake of the studied vaccines, and tailor public health messaging to diverse communities. Elucidating the extent to which ethnic patterns in vaccine refusal are driven by cultural perceptions, institutional mistrust, variation in penetrance of misinformation, and structural barriers e.g. transport, language and occupational barriers in different ethnic groups requires further study in robust surveys and qualitative research. This may inform tailoring of information dissemination strategies and misinformation countermeasures to specific groups and geographical areas. Furthermore, judicious, longitudinal monitoring of the uptake and refusal rates of vaccines in different ethnic and social groups should enable real-time assessment of developing inequalities, which may inform adaptive public health strategies. Data from this may help develop strategies for increasing uptake in these groups including developing information about vaccines in different languages for use by community leaders, faith groups, local health care providers and community champions.[30]

For peer review only

Conclusions

Certain ethnic minority, deprived populations, large households and healthier individuals were less likely to receive a vaccine, although in the unvaccinated population, higher odds of refusal was only associated with ethnicity and deprivation but not larger households nor healthier individuals. Understanding these associations may inform tailored public health messaging to different communities for equitable implementation of vaccination programs.

For peer review only

For peer review only - http://bmjopen.bmj.com/site/about/guidelines.xhtml

Author contributions

JHC and HDM obtained funding for the study. JHC extracted the data. PST, MP and AKC led data analysis and wrote first draft. PST, MP and AKC, HDM, DS, TAR, CG, FZ, BRS, CC, SJG, KK and JHC interpreted results, participated in critical revisions of manuscript and approved the final version.

Declaration of interests

PST reports previous consultation with AstraZeneca and Duke-NUS outside the submitted work. KK is a Member of the Scientific Advisory Group for Emergencies (SAGE), Member of Independent SAGE, Director of the University of Leicester Centre for Black Minority Health and Trustee of the south Asian Health Foundation. JHC is a member of several SAGE committees and chair of the risk stratification subgroup of the NERVTAG. She is unpaid director of QResearch and founder and former medical director of ClinRisk Ltd (outside the submitted work). MP, AKC, HDM, DS, TAR, FZ, BRS, SJG, CC, CG have no interests to declare.

Acknowledgments

Funding – This project was funded by a grant from the Medical Research Council (MRC Grant Ref: MR/V027778/1). JHC has received grants from the National Institute for Health Research Biomedical Research Centre, Oxford, John Fell Oxford University Press Research Fund, Cancer Research UK (grant number C5255/A18085) through the Cancer Research UK Oxford Centre, and the Oxford Wellcome Institutional Strategic Support Fund (204826/Z/16/Z) during the conduct of the study, is an unpaid director of QResearch, a not-for-profit organisation which is a partnership between the University of Oxford and EMIS Health who supply the QResearch database used for this work, and is a founder and shareholder of ClinRisk Ltd and was its medical director until 31 May 2019; ClinRisk produces open and closed source software to implement clinical risk algorithms (outside this work) into clinical computer systems. AKC is funded by a Clinical Research Training Fellowship from Cancer Research UK (grant C2195/A31310). KK is supported by NIHR Applied Collaboration East Midlands and NIHR Leicester Biomedical Research Centre. The University of Cambridge has received salary support in respect of SJG from the NHS in the East of England through the Clinical Academic Reserve. We acknowledge the contribution of EMIS practices who contribute to QResearch and EMIS Health and the Universities of Nottingham and Oxford for expertise in establishing, developing, or supporting the QResearch database. This project involves data derived from patient level information collected by

For peer review only

the NHS, as part of the care and support of cancer patients. The data are collated, maintained, and quality assured by the National Cancer Registration and Analysis Service, which is part of Public Health England (PHE). Access to the data was facilitated by the PHE Office for Data Release. The Hospital Episode Statistics data used in this analysis are reused by permission from NHS Digital, which retains the copyright in that data. We thank the Office for National Statistics (ONS) for providing the mortality data. NHS Digital, PHE, and the ONS bear no responsibility for the analysis or interpretation of the data. The investigators acknowledge the philanthropic support of the donors to the University of Oxford's COVID-19 Research Response Fund.

Role of the funding source

The funder had no role in the study design, in the collection, analysis, or interpretation of data, in the writing of the report, or in the decision to submit the paper for publication.

Data statement

To guarantee the confidentiality of personal and health information, only the authors have had access to the data during the study in accordance with the relevant license agreements. Access to QResearch data is according to the information on the QResearch website (www.qresearch.org).

Ethics approval

This was part of a larger project which has been independently peer-reviewed and received ethics approval by the QResearch Scientific board (REC 18/EM/0400; project reference OX102).

For peer review only

References

[1] D. R. Juliet M. Bartleson, Anthony J. Covarrubias, David Furman, Daniel A. Winer & Eric Verdin "SARS-CoV-2, COVID-19 and the aging immune system," *Nat Aging,* vol. 1, pp. 769–782, 2021. [Online]. Available: https://www.nature.com/articles/s43587-021-00114-7.

[2] P. H. England. "Pneumococcal Polysaccharide Vaccine (PPV) coverage report, England, April 2019 to March 2020." https://assets.publishing.service.gov.uk/government/uploads/system/uploads/attachment_data/file/899458/hpr1320_ppv-vc.pdf (accessed.

[3] P. H. England. "Seasonal influenza vaccine uptake in GP patients: winter season 2020 to 2021." Public Health England. https://assets.publishing.service.gov.uk/government/uploads/system/uploads/attachment_data/file/996033/Annual-Report_SeasonalFlu-Vaccine_GPs_2020_to_2021.pdf (accessed.

[4] P. H. England. "Shingles vaccine coverage report (adults eligible from April to September 2020 and vaccinated to end of December 2020): England

Quarter 2 report of financial year 2020 to 2021." https://assets.publishing.service.gov.uk/government/uploads/system/uploads/attachment_data/file/955523/SP_hpr0321_SHNGLS-vc_Q2-final-3_refs.pdf (accessed.

[5] W. Europe. "Evaluation of seasonal influenza vaccination policies and coverage in the WHO European Region." https://www.euro.who.int/__data/assets/pdf_file/0003/241644/Evaluation-of-seasonal-influenza-vaccination-policies-and-coverage-in-the-WHO-European-Region.pdf (accessed.

[6] D. P. Chan, N. S. Wong, E. L. Wong, A. W. Cheung, and S. S. Lee, "Household characteristics and influenza vaccination uptake in the community-dwelling elderly: a cross-sectional study," (in eng), *Prev Med Rep,* vol. 2, pp. 803-8, 2015, doi: 10.1016/j.pmedr.2015.09.002.

[7] M. M. Loiacono *et al.*, "Patient and practice level factors associated with seasonal influenza vaccine uptake among at-risk adults in England, 2011 to 2016: An age-stratified retrospective cohort study," (in eng), *Vaccine X,* vol. 4, p. 100054, Apr 2020, doi: 10.1016/j.jvacx.2020.100054.

[8] C. Coupland *et al.*, "Inequalities in uptake of influenza vaccine by deprivation and risk group: time trends analysis," (in eng), *Vaccine,* vol. 25, no. 42, pp. 7363-71, Oct 2007, doi: 10.1016/j.vaccine.2007.08.032.

[9] H. Fisher, C. L. Trotter, S. Audrey, K. MacDonald-Wallis, and M. Hickman, "Inequalities in the uptake of human papillomavirus vaccination: a systematic review and meta-analysis," (in eng), *Int J Epidemiol,* vol. 42, no. 3, pp. 896-908, Jun 2013, doi: 10.1093/ije/dyt049.

[10] T. House and M. J. Keeling, "Household structure and infectious disease transmission," (in eng), *Epidemiol Infect,* vol. 137, no. 5, pp. 654-61, May 2009, doi: 10.1017/S0950268808001416.

[11] L. Rockliffe, J. Waller, L. A. Marlow, and A. S. Forster, "Role of ethnicity in human papillomavirus vaccination uptake: a cross-sectional study of girls from ethnic minority groups attending London schools," (in eng), *BMJ Open,* vol. 7, no. 2, p. e014527, 02 2017, doi: 10.1136/bmjopen-2016-014527.

[12] A. R. Sarker, R. Akram, N. Ali, and M. Sultana, "Coverage and factors associated with full immunisation among children aged 12-59 months in Bangladesh: insights from the nationwide cross-sectional demographic and health survey," (in eng), *BMJ Open,* vol. 9, no. 7, p. e028020, 07 2019, doi: 10.1136/bmjopen-2018-028020.

For peer review only - http://bmjopen.bmj.com/site/about/guidelines.xhtml

[13] GOV.UK. "Population of England and Wales." https://www.ethnicity-facts-figures.service.gov.uk/uk-population-by-ethnicity/national-and-regional-populations/population-of-england-and-wales/latest (accessed.
[14] N. Digital. "Patients registered at a GP practice." https://digital.nhs.uk/data-and-information/data-tools-and-services/data-services/general-practice-data-hub/patients-registered-at-a-gp-practice (accessed.
[15] Gov.UK. "Vaccination against shingles guide." https://www.gov.uk/government/publications/shingles-vaccination-for-adults-aged-70-or-79-years-of-age-a5-leaflet/vaccination-against-shingles-guide (accessed 14 April 2022.
[16] "QResearch Web." https://qweb.qresearch.org (accessed.
[17] Townsend P and D. N. . *The Black report*. Penguin 1982.
[18] "2011 UK Townsend Deprivation Scores - Townsend Deprivation Scores Report," 2011. [Online]. Available: https://statistics.ukdataservice.ac.uk/dataset/2011-uk-townsend-deprivation-scores/resource/f500b512-2f49-4e6f-8403-9417b9133f64.
[19] D. Rubin, *Multiple Imputation for Non-response in Surveys*. John Wiley, 1987.
[20] E. I. Benchimol *et al.*, "The REporting of studies Conducted using Observational Routinely-collected health Data (RECORD) statement," (in eng), *PLoS Med,* vol. 12, no. 10, p. e1001885, Oct 2015, doi: 10.1371/journal.pmed.1001885.
[21] *Stata Statistical Software: Release 17*. StataCorp. , College Station, TX: StataCorp LLC.
[22] "Green Book - Pneumococcal " 2018. [Online]. Available: https://assets.publishing.service.gov.uk/government/uploads/system/uploads/attachment_data/file/674074/GB_Chapter_25_Pneumococcal_V7_0.pdf.
[23] "Seasonal influenza vaccine uptake in GP patients: winter season 2020 to 2021." Public Health England. https://assets.publishing.service.gov.uk/government/uploads/system/uploads/attachment_data/file/996033/Annual-Report_SeasonalFlu-Vaccine_GPs_2020_to_2021.pdf (accessed 20 April 2022.
[24] S. P. Bhatt *et al.*, "Smoking duration alone provides stronger risk estimates of chronic obstructive pulmonary disease than pack-years," (in eng), *Thorax,* vol. 73, no. 5, pp. 414-421, 05 2018, doi: 10.1136/thoraxjnl-2017-210722.
[25] M. Śliwińska-Mossoń and H. Milnerowicz, "The impact of smoking on the development of diabetes and its complications," (in eng), *Diab Vasc Dis Res,* vol. 14, no. 4, pp. 265-276, 07 2017, doi: 10.1177/1479164117701876.
[26] S. Abbott and M. Riga, "Delivering services to the Bangladeshi community: the views of healthcare professionals in East London," (in eng), *Public Health,* vol. 121, no. 12, pp. 935-41, Dec 2007, doi: 10.1016/j.puhe.2007.04.014.
[27] S. Latif, I. Ahmed, M. S. Amin, I. Syed, and N. Ahmed, "Exploring the potential impact of health promotion videos as a low cost intervention to reduce health inequalities: a pilot before and after study on Bangladeshis in inner-city London," (in eng), *London J Prim Care (Abingdon),* vol. 8, no. 4, pp. 66-71, 2016, doi: 10.1080/17571472.2016.1208382.
[28] V. Nafilyan *et al.*, "Sociodemographic inequality in COVID-19 vaccination coverage among elderly adults in England: a national linked data study," (in eng), *BMJ Open,* vol. 11, no. 7, p. e053402, 07 23 2021, doi: 10.1136/bmjopen-2021-053402.
[29] C. G. Victora *et al.*, "The Inverse Equity Hypothesis: Analyses of Institutional Deliveries in 286 National Surveys," (in eng), *Am J Public Health,* vol. 108, no. 4, pp. 464-471, 04 2018, doi: 10.2105/AJPH.2017.304277.
[30] W. Hanif, S. N. Ali, K. Patel, and K. Khunti, "Cultural competence in covid-19 vaccine rollout," *BMJ,* no. 371, p. m4845, 2020, doi: doi.org/10.1136/bmj.m4845.

For peer review only - http://bmjopen.bmj.com/site/about/guidelines.xhtml

For peer review only

For peer review only - http://bmjopen.bmj.com/site/about/guidelines.xhtml

Table 1: Characteristics of study population in patients aged 65+ (70+ for shingles)

Characteristics		Study population	Vaccine uptake		
		Overall	Influenza	Pneumococcal	Shingles[a]
Total	N (row %)	2054463	1711465 (83.3)	1391228 (67.7)	690783 (53.4)
Age	Mean (SD)	75.5 (7.7)	76.3 (7.7)	77.1 (7.5)	77.2 (4.4)
	65-69	541272 (26.3)	373566 (21.8)	232831 (16.7)	-
	70-79	922198 (44.9)	793150 (46.3)	665037 (47.8)	469684 (68.0)
	80-89	471167 (22.9)	434074 (25.4)	395456 (28.4)	221099 (32.0)
	90-99	119826 (5.8)	110675 (6.5)	97904 (7.0)	-
Sex	Female	1100957 (53.6)	926592 (54.1)	749022 (53.8)	365203 (52.9)
	Male	953506 (46.4)	784873 (45.9)	642206 (46.2)	325580 (47.1)
Ethnicity	White	1522868 (74.1)	1293856 (75.6)	1064331 (76.5)	539237 (78.1)
	Indian	35618 (1.7)	31062 (1.8)	25454 (1.8)	11293 (1.6)
	Pakistani	17555 (0.9)	15588 (0.9)	12090 (0.9)	4388 (0.6)
	Bangladeshi	8138 (0.4)	7635 (0.4)	6264 (0.5)	2076 (0.3)
	Other Asian	17848 (0.9)	15171 (0.9)	11890 (0.9)	5135 (0.7)
	Black Caribbean	22859 (1.1)	18010 (1.1)	14102 (1.0)	5791 (0.8)
	Black African	16880 (0.8)	13530 (0.8)	9545 (0.7)	3518 (0.5)
	Chinese	6553 (0.3)	4835 (0.3)	3507 (0.3)	1502 (0.2)
	Other ethnic groups	25410 (1.2)	19778 (1.2)	14569 (1.0)	5832 (0.8)
	Ethnicity not recorded	380734 (18.5)	292000 (17.1)	229476 (16.5)	112011 (16.2)
Region	East Midlands	46002 (2.2)	38777 (2.3)	30526 (2.2)	16779 (2.4)
	East of England	93217 (4.5)	77645 (4.5)	64843 (4.7)	34167 (4.9)
	London	322941 (15.7)	261176 (15.3)	204112 (14.7)	92174 (13.3)
	North East	47496 (2.3)	40081 (2.3)	33271 (2.4)	15848 (2.3)
	North West	417970 (20.3)	354779 (20.7)	292600 (21.0)	140099 (20.3)
	South Central	283054 (13.8)	239109 (14.0)	199347 (14.3)	102632 (14.9)
	South East	268594 (13.1)	220952 (12.9)	179031 (12.9)	91516 (13.2)
	South West	256384 (12.5)	213037 (12.4)	169824 (12.2)	87179 (12.6)
	West Midlands	237881 (11.6)	197414 (11.5)	161606 (11.6)	81942 (11.9)
	Yorkshire & Humber	80924 (3.9)	68495 (4.0)	56068 (4.0)	28447 (4.1)
Deprivation quintile	1 (most affluent)	674004 (32.8)	569701 (33.3)	471575 (33.9)	251660 (36.4)
	2	547862 (26.7)	456956 (26.7)	373336 (26.8)	191172 (27.7)
	3	385476 (18.8)	318962 (18.6)	258842 (18.6)	123090 (17.8)
	4	267458 (13.0)	219941 (12.9)	175665 (12.6)	78550 (11.4)
	5 (most deprived)	174280 (8.5)	141551 (8.3)	108526 (7.8)	44651 (6.5)
	Not recorded	5383 (0.3)	4354 (0.3)	3284 (0.2)	1660 (0.2)
Home category	Neither in care home nor homeless	2005725 (97.6)	1665389 (97.3)	1356313 (97.5)	682316 (98.8)
	Care home	47655 (2.3)	45263 (2.6)	34352 (2.5)	8301 (1.2)
	Homeless	1083 (0.1)	813 (<0.01)	563 (<0.01)	166 (<0.01)
Household size	1 person	875588 (42.6)	726447 (42.4)	596361 (42.9)	285715 (41.4)
	2 people	849357 (41.3)	721411 (42.2)	594481 (42.7)	326499 (47.3)

For peer review only - http://bmjopen.bmj.com/site/about/guidelines.xhtml

	3-5 people	255089 (12.4)	199611 (11.7)	152373 (11.0)	65031 (9.4)
	6-9 people	30961 (1.5)	24934 (1.5)	18767 (1.3)	6678 (1.0)
	10 or more	43468 (2.1)	39062 (2.3)	29246 (2.1)	6860 (1.0)
Number of health conditions[c]	0	667163 (32.5)	483507 (28.3)	566398 (40.7)	213919 (31.0)
	1	786798 (38.3)	671330 (39.2)	559648 (40.2)	281353 (40.7)
	2	428751 (20.9)	393220 (23.0)	215126 (15.5)	145583 (21.1)
	3+	171751 (8.4)	163408 (9.5)	50056 (3.6)	49928 (7.2)

[a]Percentage calculated using denominator of shingles eligible population, n = 1,294,176. Percentages are column percentages unless otherwise indicated. SD: standard deviation. [c]Counts only based on conditions included in this study.

For peer review only

Figure labels and footnotes

Figure 1: Box and whiskers diagrams summarising influenza, pneumococcal and shingles vaccination uptake/refusal rates in practices across different regions in England. The mid-line of box represents median uptake/refusal rate, lower and upper boundaries of box represent first and third quartile, lower and upper whiskers represent minimum and maximum rates. Each individual dot was also presented to represent individual practice uptake/refusal rate.

Figure 2: Associations of ethnicity, deprivation, household size and number of health conditions on influenza, pneumococcal and shingles vaccine uptake.

Footnote: Logistic models for ethnicity, deprivation, household size and health conditions were run separately as each exposure factor required different sets of adjustment variables as informed by DAG evaluation. The following adjustment covariates were included in each of these models as the following: (1) Ethnicity – no adjustment; (2) Deprivation - adjusted for age, sex, region, ethnicity, household size; (3) Household size – adjusted for age, sex, region, ethnicity, deprivation, (4) Health conditions – adjusted for age, sex, region, ethnicity, deprivation, household size, house type, smoking and BMI.

Figure 3: Associations of ethnicity, deprivation, household size and number of health conditions on influenza, pneumococcal and shingles vaccine refusal in the unvaccinated population.

Footnote: Logistic models for ethnicity, deprivation, household size and health conditions were run separately as each exposure factor required different sets of adjustment variables as informed by DAG evaluation. The following adjustment covariates were included in each of these models as the following: (1) Ethnicity – no adjustment; (2) Deprivation - adjusted for age, sex, region, ethnicity, household size; (3) Household size – adjusted for age, sex, region, ethnicity, deprivation, (4) Health conditions – adjusted for age, sex, region, ethnicity, deprivation, household size, house type, smoking and BMI.

For peer review only - http://bmjopen.bmj.com/site/about/guidelines.xhtml

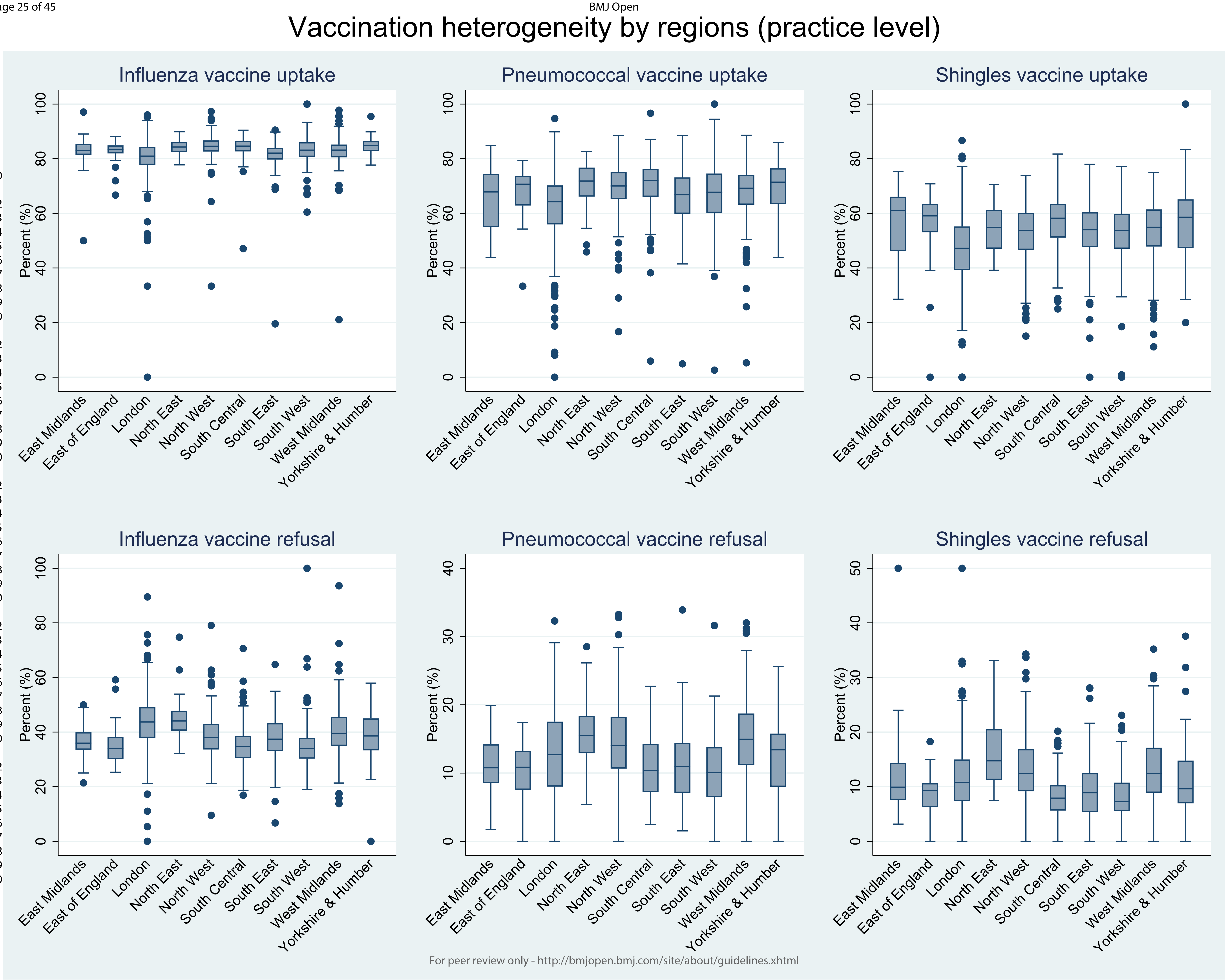
Vaccination heterogeneity by regions (practice level)
Influenza vaccine uptake
Pneumococcal vaccine uptake
Shingles vaccine uptake
Influenza vaccine refusal
Pneumococcal vaccine refusal
Shingles vaccine refusal
Percent (%)
East Midlands
East of England
London
North East
North West
South Central
South East
South West
West Midlands
Yorkshire & Humber

Uptake

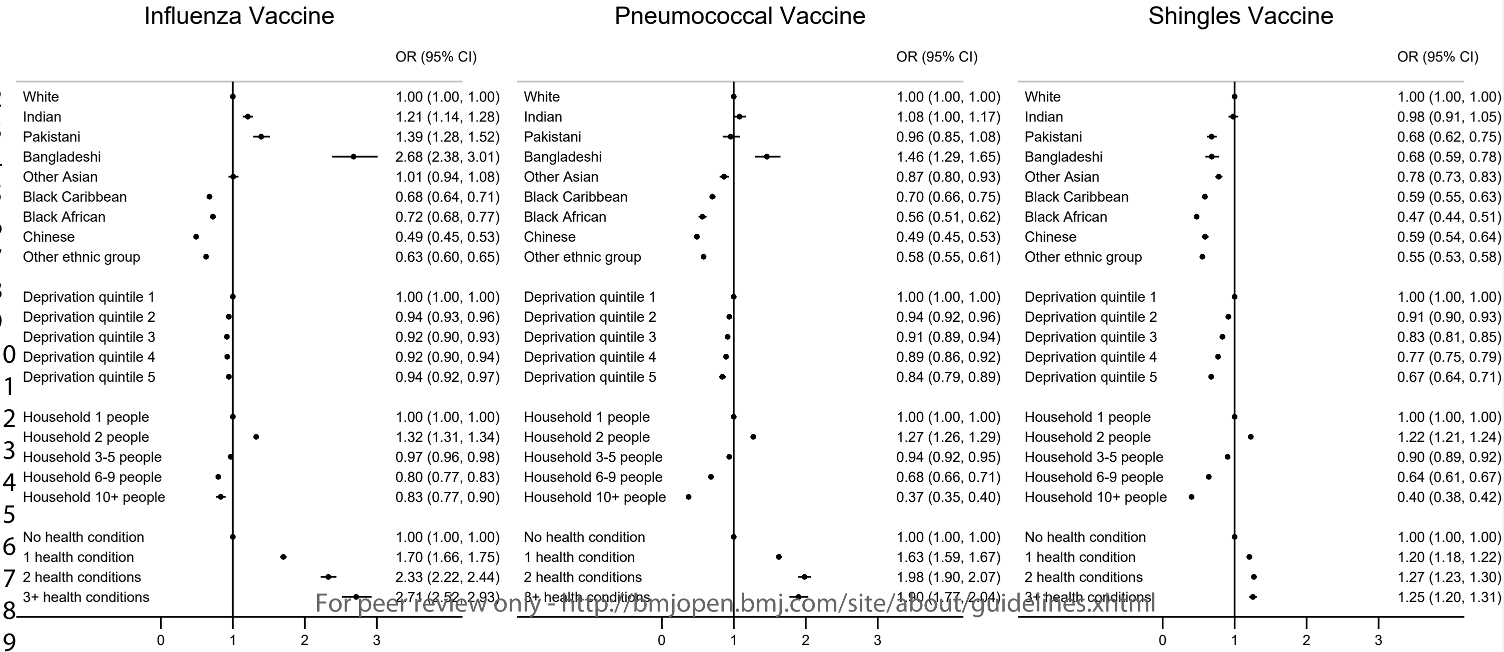

For peer review only - http://bmjopen.bmj.com/site/about/guidelines.xhtml

Vaccination refusal (unvaccinated)

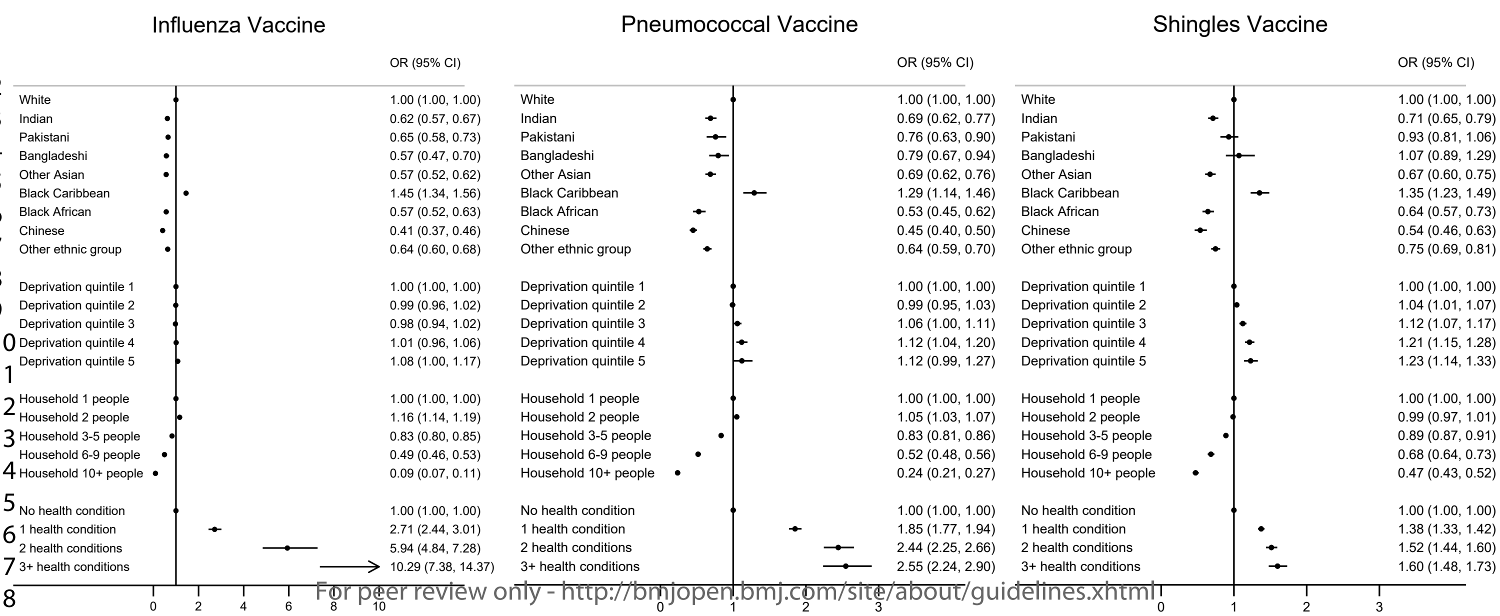

For peer review only - http://bmjopen.bmj.com/site/about/guidelines.xhtml

Supplement

Table S1: Characteristics of study population - lifestyle and health conditions

Characteristics		Study population	Vaccine uptake		
		Overall	Influenza	Pneumococcal	Shingles[a]
Total	N (row %)	2054463	1711465 (83.3)	1391228 (67.7)	690783 (53.4)
Body mass index	<18.5	36406 (1.8)	31088 (1.8)	25321 (1.8)	9351 (1.4)
	18.5-25	615113 (29.9)	515261 (30.1)	421175 (30.3)	204730 (29.6)
	25-30	754859 (36.7)	641998 (37.5)	528282 (38.0)	273645 (39.6)
	30-35	361993 (17.6)	310919 (18.2)	254754 (18.3)	128653 (18.6)
	35-40	121452 (5.9)	105199 (6.1)	85649 (6.2)	41349 (6.0)
	>=40	48792 (2.4)	42948 (2.5)	34151 (2.5)	15061 (2.2)
	Not recorded	115848 (5.6)	64052 (3.7)	41896 (3.0)	17994 (2.6)
Smoking	Non-smoker	1143669 (55.7)	955785 (55.8)	773504 (55.6)	383407 (55.5)
	Ex-smoker	712384 (34.7)	618783 (36.2)	516754 (37.1)	265778 (38.5)
	Current smoker	177685 (8.6)	132076 (7.7)	98773 (7.1)	40903 (5.9)
	Not recorded	20725 (1.0)	4821 (0.3)	2197 (0.2)	695 (0.1)
Health conditions	Asthma	254110 (12.4)	235822 (13.8)	162658 (11.7)	89598 (13.0)
	Chronic obstructive pulmonary disease	160907 (7.8)	150873 (8.8)	66827 (4.8)	52655 (7.6)
	Type-1 diabetes	6253 (0.3)	5908 (0.3)	4243 (0.3)	1882 (0.3)
	Type-2 diabetes	353860 (17.2)	327748 (19.2)	183136 (13.2)	120912 (17.5)
	Hypertension	1013241 (49.3)	901041 (52.6)	559319 (40.2)	360378 (52.2)
	Dementia	86868 (4.2)	81151 (4.7)	8622 (0.6)	10989 (1.6)
	Parkinson's disease	20720 (1.0)	18825 (1.1)	4635 (0.3)	5467 (0.8)
	Epilepsy	38404 (1.9)	33738 (2.0)	19335 (1.4)	10874 (1.6)
	Cerebral palsy	1041 (0.1)	929 (0.1)	598 (0.0)	233 (0.0)
	Learning disability	39959 (1.9)	36644 (2.1)	9192 (0.7)	9897 (1.4)
	Severe mental illness	243791 (11.9)	210885 (12.3)	133322 (9.6)	73294 (10.6)
	Coronary heart disease	294490 (14.3)	273488 (16.0)	153850 (11.1)	101948 (14.8)
	Atrial fibrillation	196503 (9.6)	180461 (10.5)	53438 (3.8)	55647 (8.1)
	Congestive cardiac failure	85674 (4.2)	79600 (4.7)	19891 (1.4)	20144 (2.9)
	Congenital heart disease	14739 (0.7)	13500 (0.8)	6590 (0.5)	4938 (0.7)
	Immunosuppression	17339 (0.8)	16188 (0.9)	8622 (0.6)	3445 (0.5)

[a]Percentage calculated using denominator of shingles eligible population, n = 1,294,176. [b] Comorbidities diagnosed prior to vaccinations in those vaccinated. Percentages are column percentages unless otherwise indicated. SD: standard deviation.

For peer review only

Figure S1: Directed acyclic graphs (DAGs) modelling exposures and corresponding outcomes. DAGs were used to map out the relationships between exposure and outcome of interest, and how they were related to other covariates to evaluate which variables were considered a confounder and would need to be adjusted for in the regression models.

Interpretation of DAGs
Green circles denote exposure and blue circle with "I" denote outcome.
White circles denote adjusted covariates while other blue circles denote variables not for adjustment in each model.

Model 1:
Exposure: Ethnicity
Outcome: Vaccination uptake/refusal
Confounder adjustment: None (no other variables were identified as a confounder for the association between ethnicity and vaccine uptake/refusal)

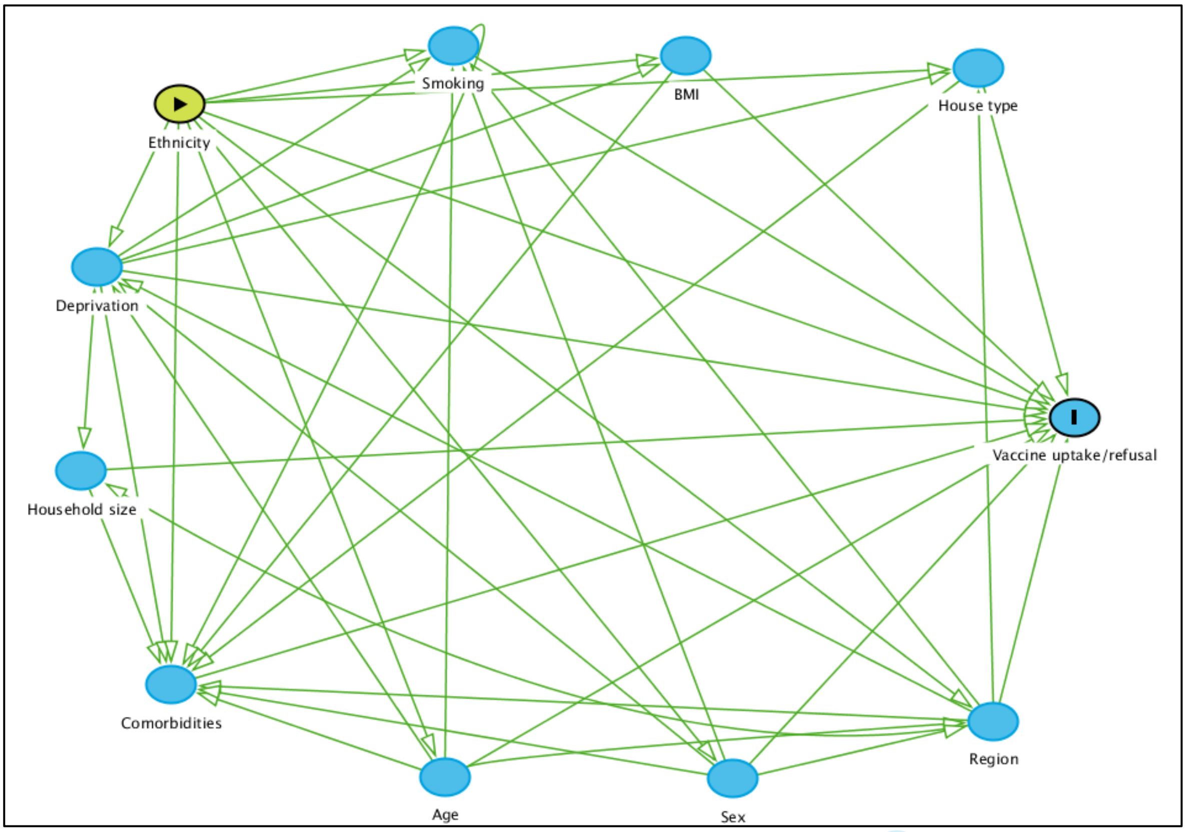

Model 2:
Exposure: Deprivation
Outcome: Vaccination uptake/refusal
Confounder adjustment: age, sex, region, ethnicity, household size (identified as confounders for the association between deprivation and vaccine uptake/refusal)

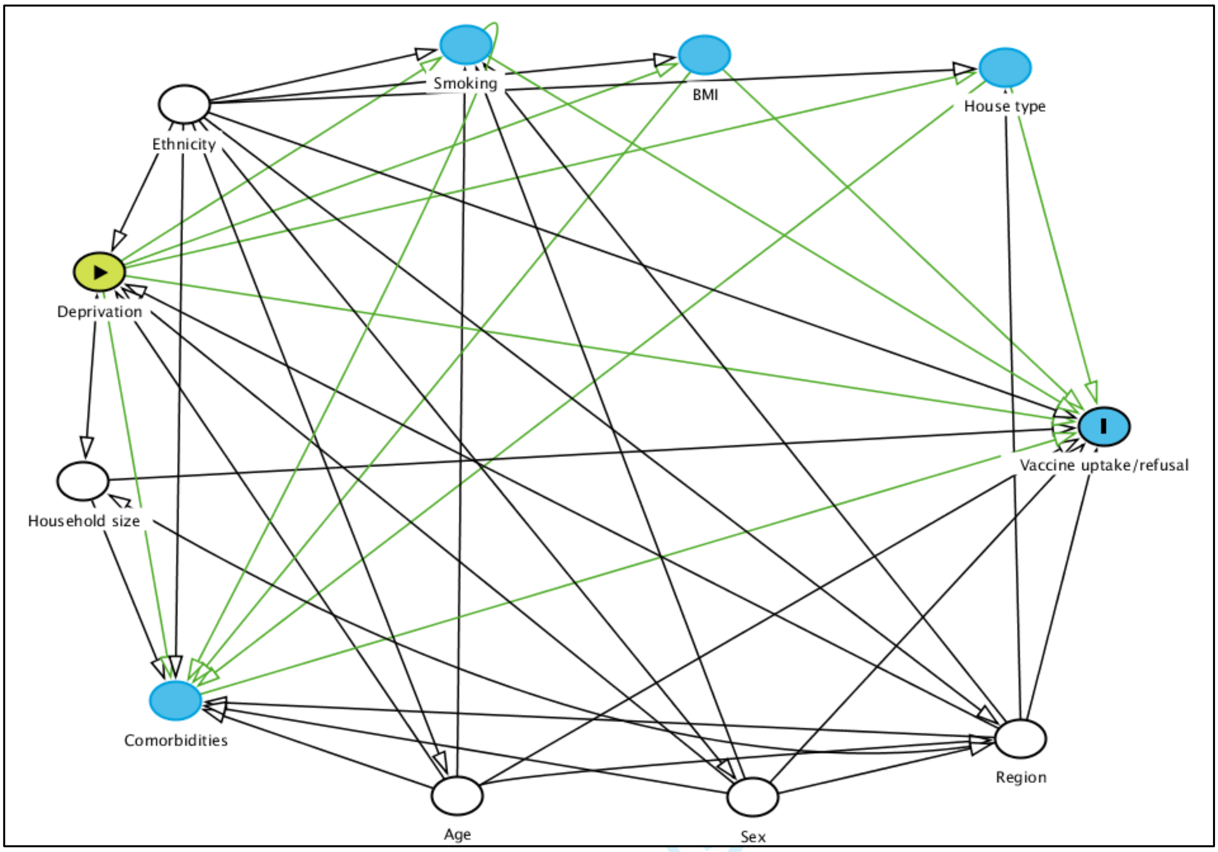

Model 3:
Exposure: Household size
Outcome: Vaccination uptake/refusal
Confounder adjustment: age, sex, region, ethnicity, deprivation (identified as confounders for the association between household size and vaccine uptake/refusal)

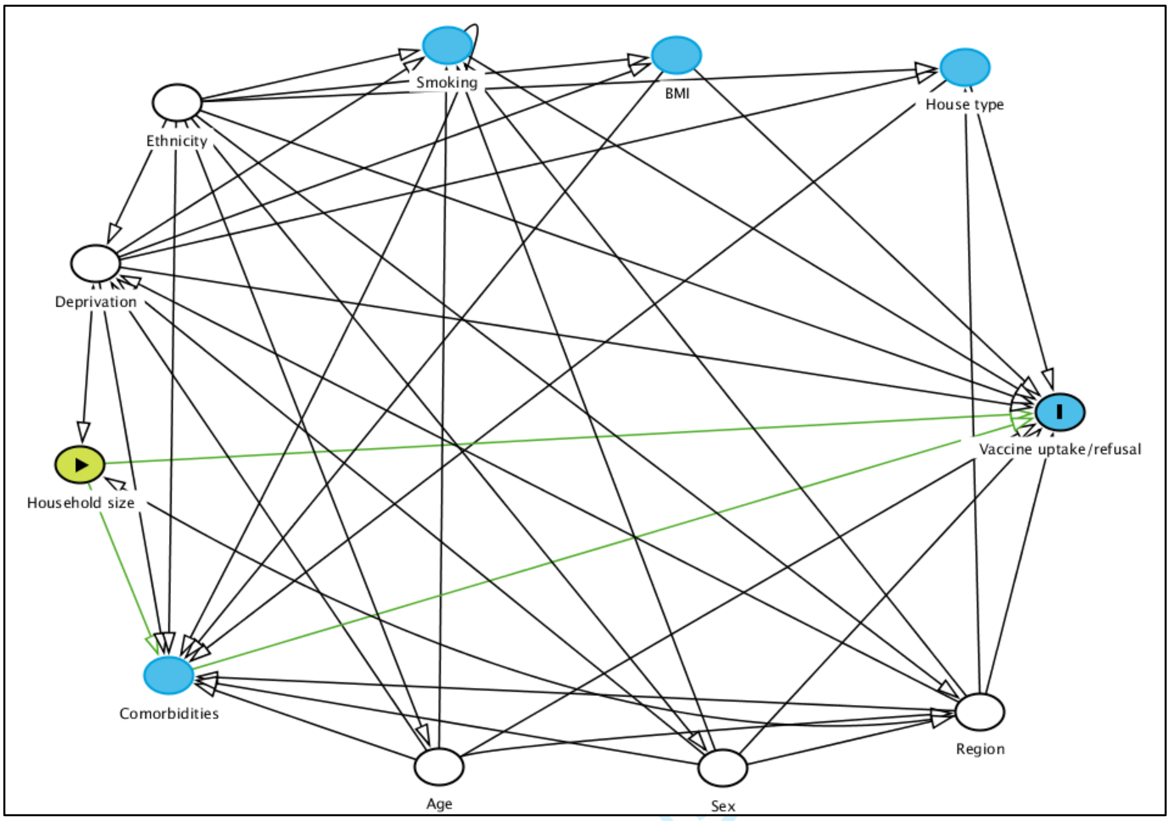

For peer review only

Model 4:
Exposure: Health conditions (comorbidities)
Outcome: Vaccination uptake/refusal
Confounder adjustment: age, sex, region, ethnicity, deprivation, household size, house type, smoking, BMI (identified as confounders for the association between health conditions (comorbidities) and vaccine uptake/refusal)

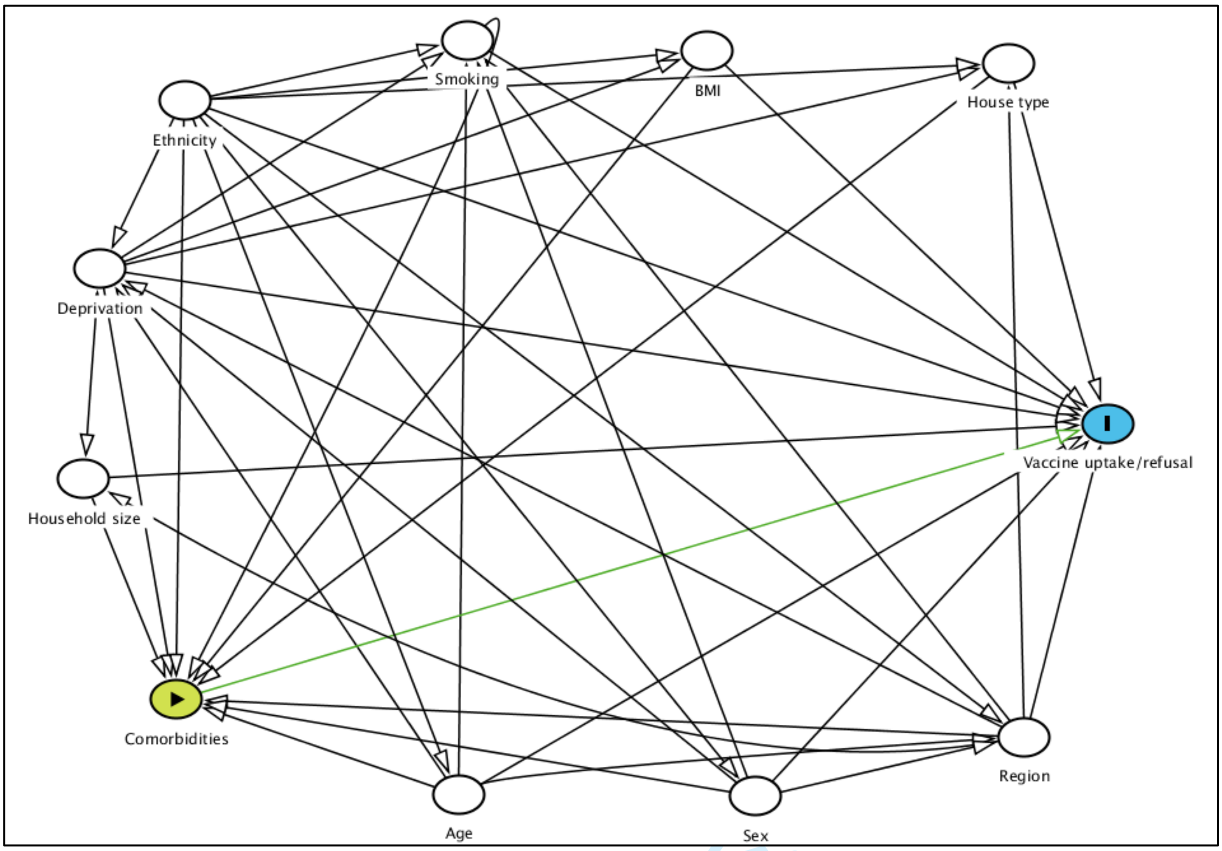

For peer review only - http://bmjopen.bmj.com/site/about/guidelines.xhtml

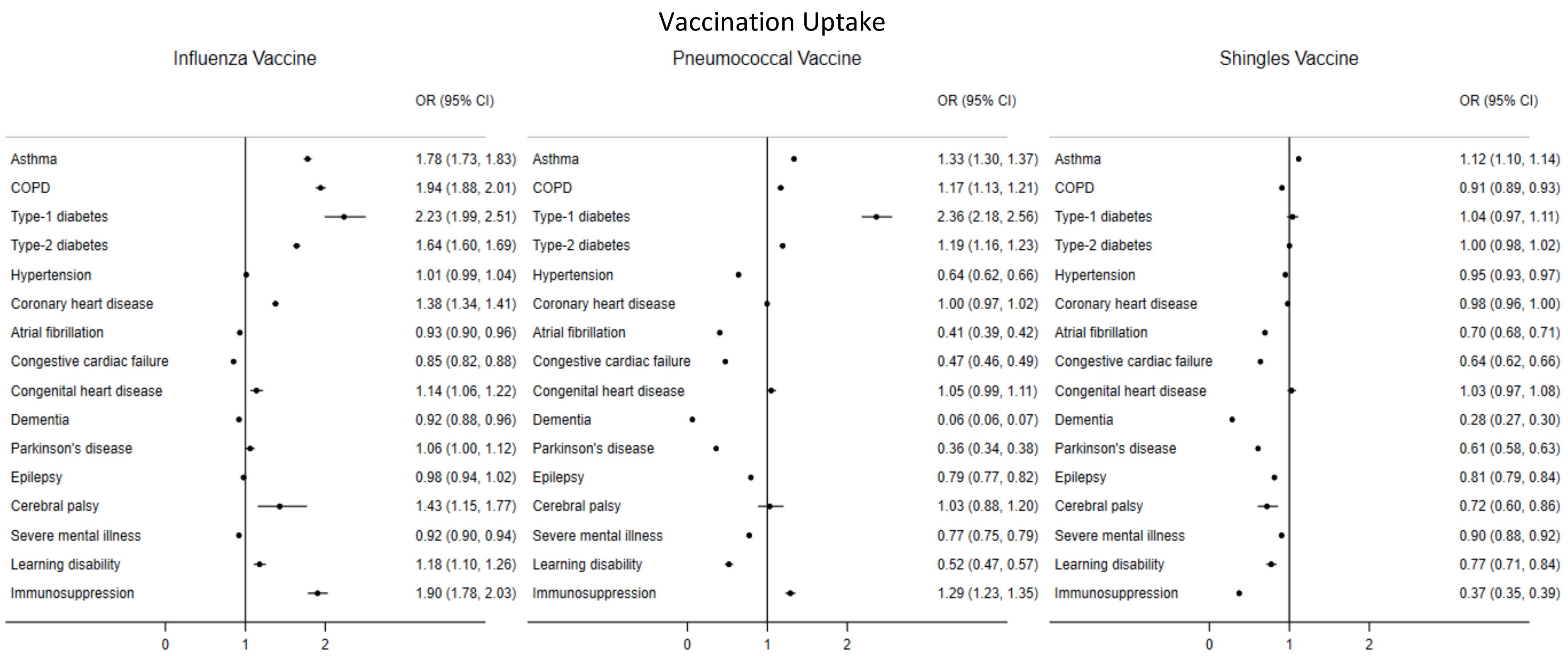

Figure S2: Associations of vaccine uptake and specific health conditions.

For peer review only - http://bmjopen.bmj.com/site/about/guidelines.xhtml

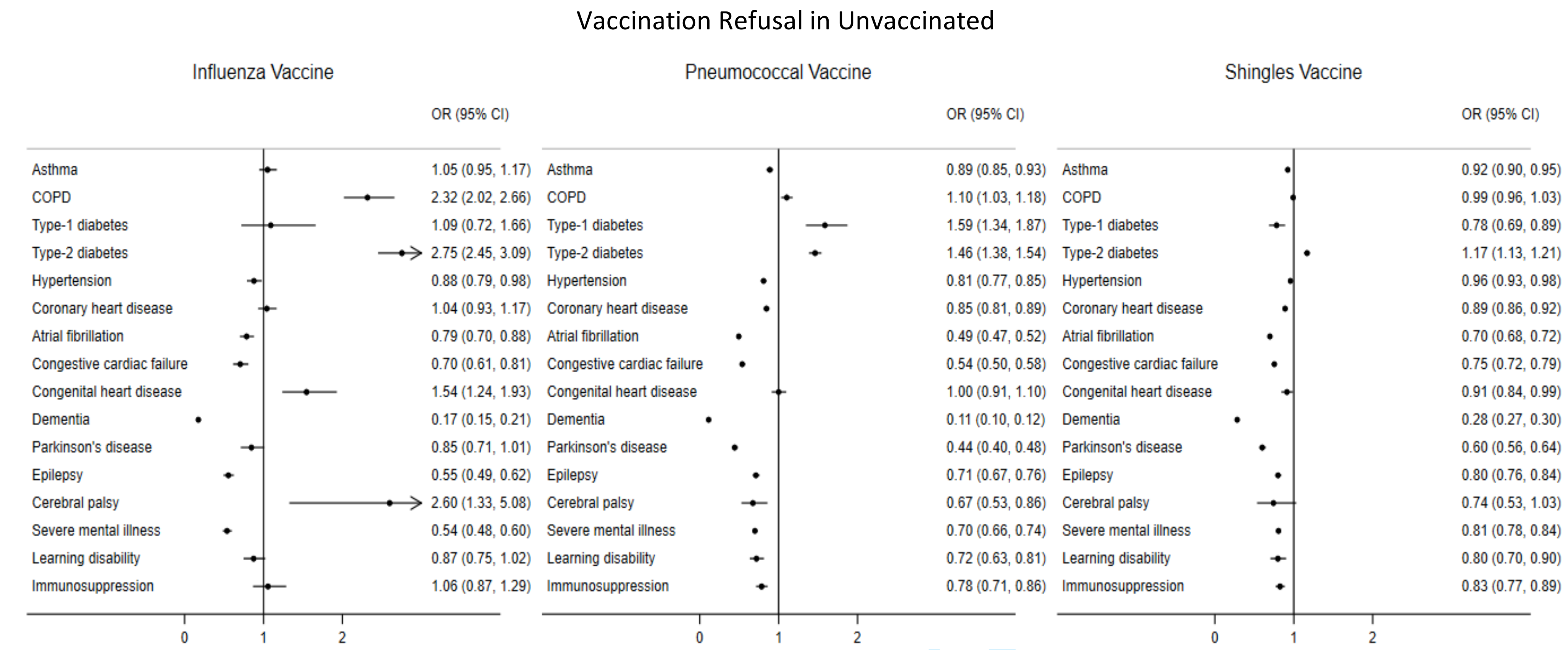

Figure S3: Associations of vaccine refusal in unvaccinated and specific health conditions.

Figure S4: Interaction analyses for vaccine uptake: ethnicity and deprivation

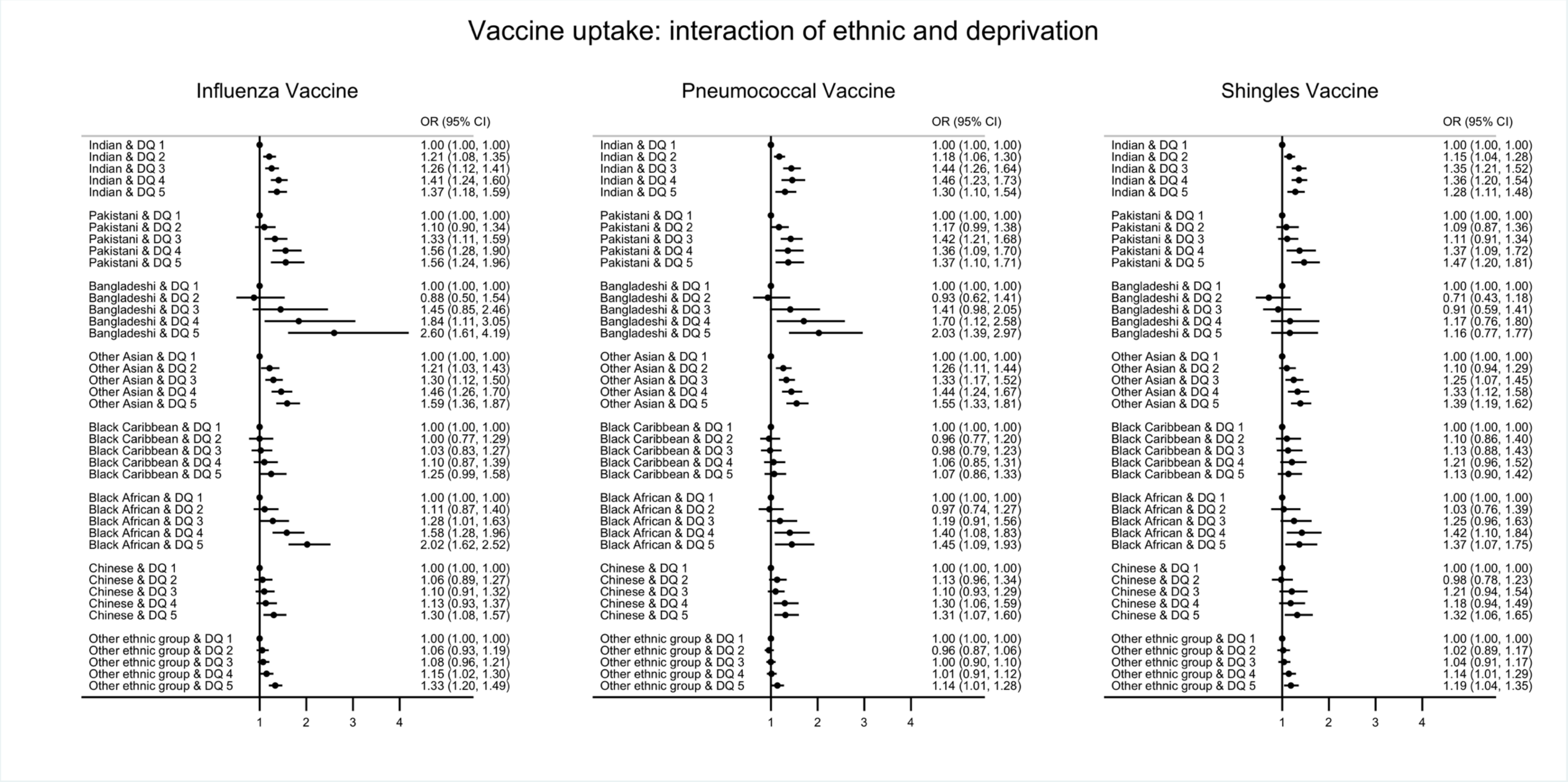

Interaction p values were <0.01 for uptake of each vaccines evaluated; DQ: deprivation quintile

Figure S5: Interaction analyses for vaccine uptake: ethnicity and household size

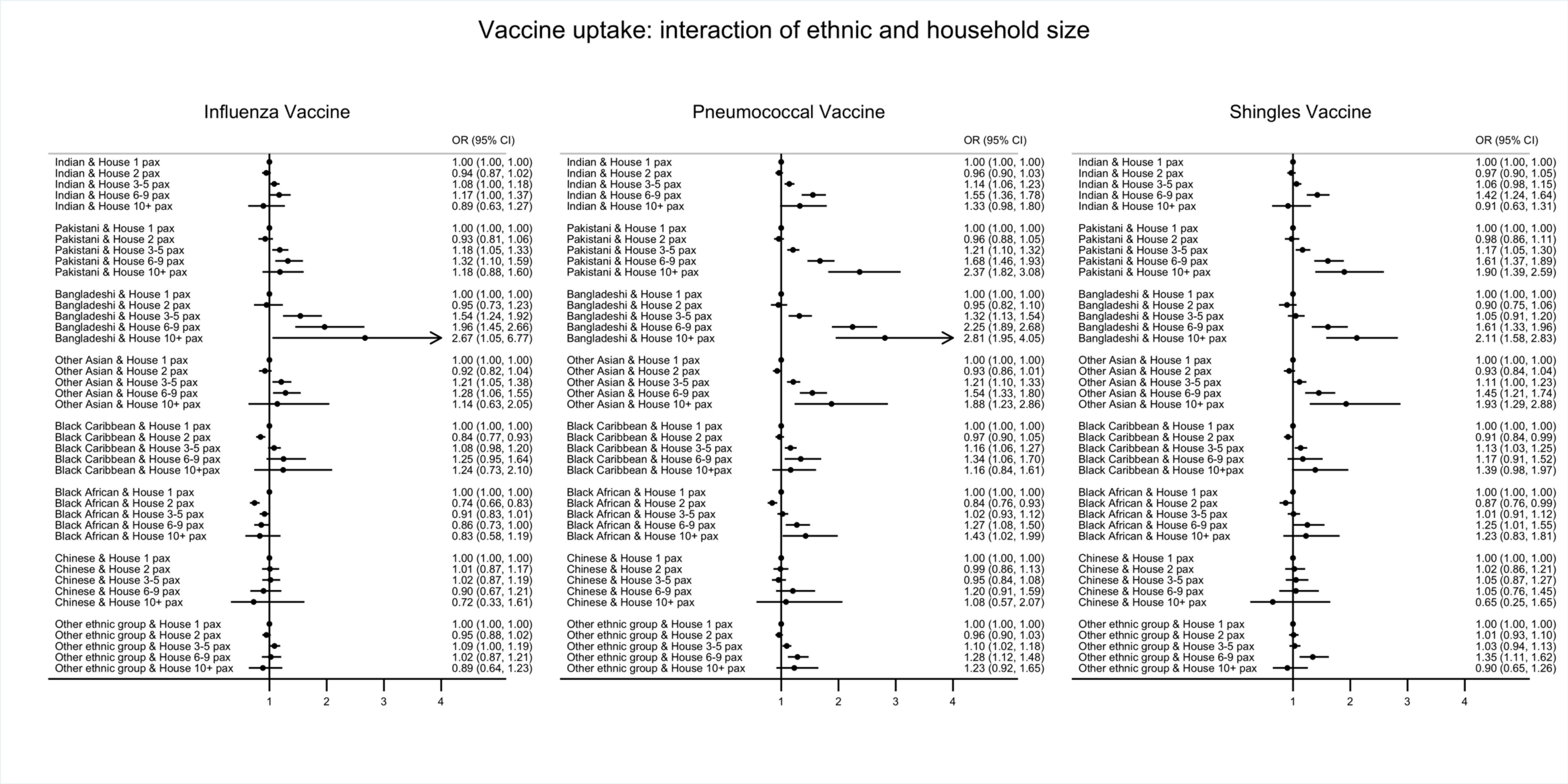

Interaction p values were <0.01 for uptake of each vaccines evaluated.

Figure S6: Interaction analyses for vaccine uptake: ethnicity and number of health conditions

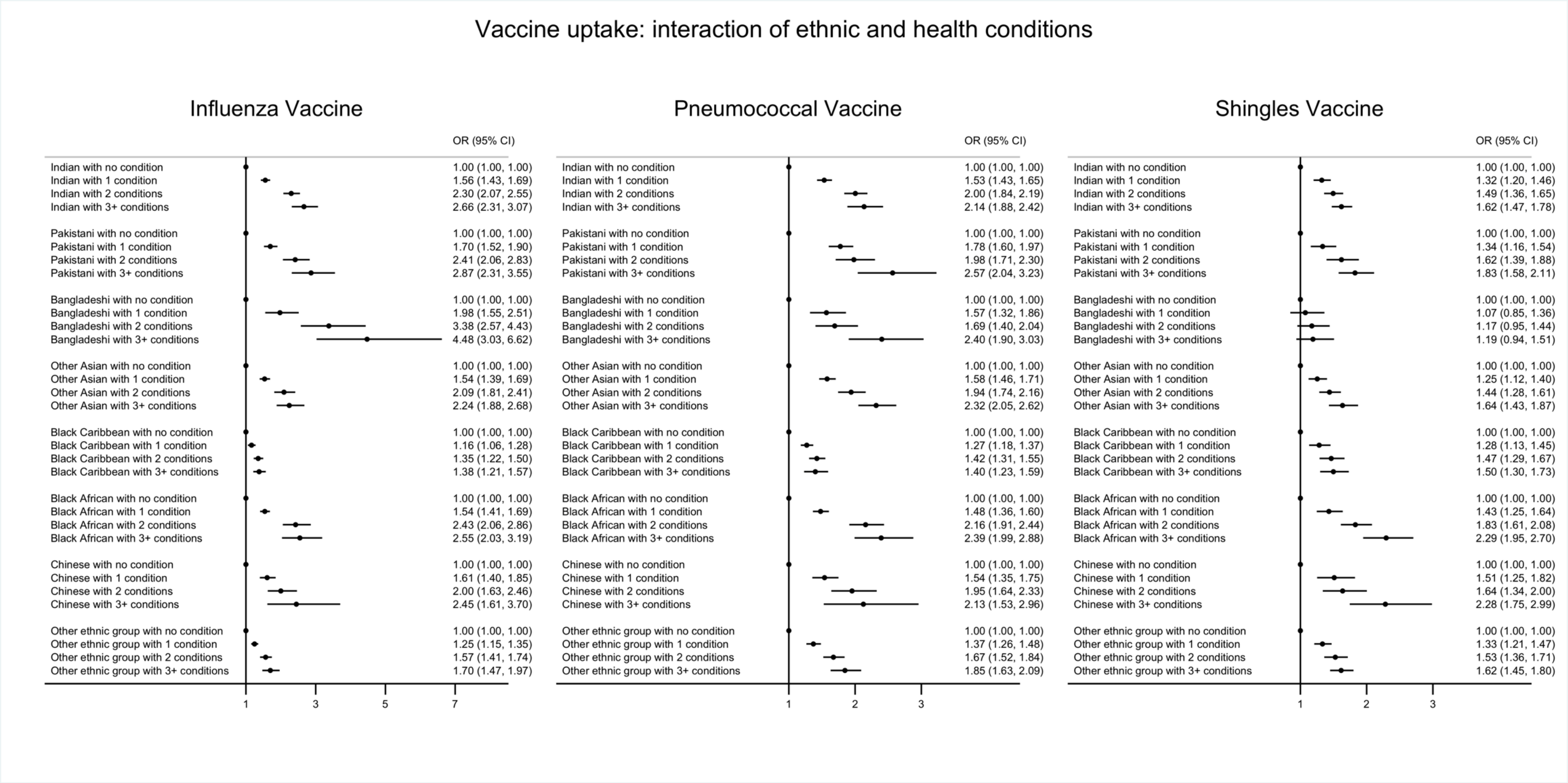

Interaction p values were <0.01 for uptake of each vaccines evaluated.

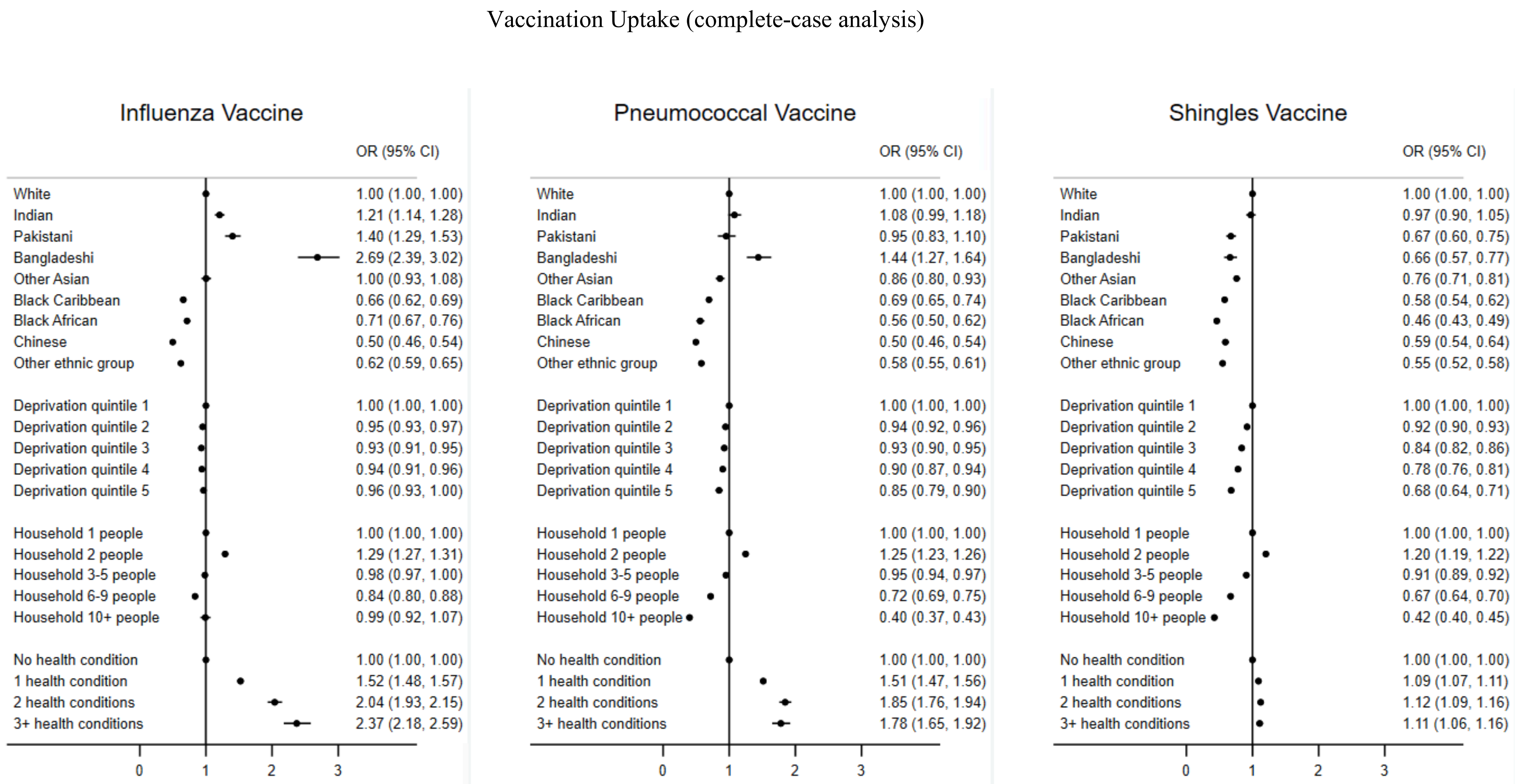

Figure S7: Sensitivity (complete-case analysis): associations of vaccine uptake and ethnic group, deprivation, household size and health conditions.

Vaccination Refusal in Unvaccinated (complete-case analysis)

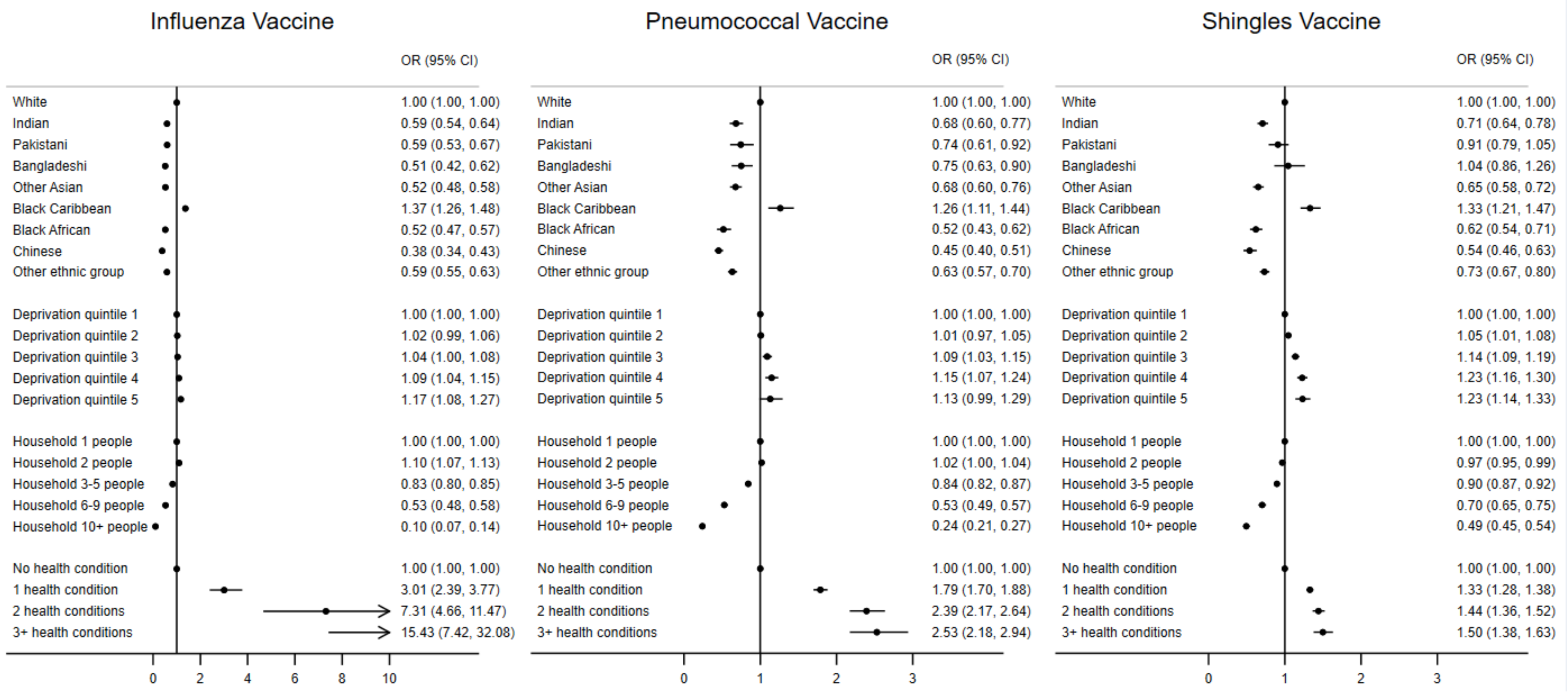

Figure S8: Sensitivity (complete-case analysis): associations of vaccine refusal (in non-vaccinated) and ethnic group, deprivation, household size and health conditions.

For peer review only

The RECORD statement – checklist of items, extended from the STROBE statement, that should be reported in observational studies using routinely collected health data.[1]

	Item No.	STROBE items	Location in manuscript where items are reported	RECORD items	Location in manuscript where items are reported
**Title and abstract**					
	1	(a) Indicate the study's design with a commonly used term in the title or the abstract (b) Provide in the abstract an informative and balanced summary of what was done and what was found	Pages 1-2	RECORD 1.1: The type of data used should be specified in the title or abstract. When possible, the name of the databases used should be included.<br><br>RECORD 1.2: If applicable, the geographic region and timeframe within which the study took place should be reported in the title or abstract.<br><br>RECORD 1.3: If linkage between databases was conducted for the study, this should be clearly stated in the title or abstract.	Page 1-2
**Introduction**					
Background rationale	2	Explain the scientific background and rationale for the investigation being reported	Page 4		
Objectives	3	State specific objectives, including any prespecified hypotheses	Page 5		
**Methods**					
Study Design	4	Present key elements of study design early in the paper	Page 6		
Setting	5	Describe the setting, locations, and relevant dates, including periods of recruitment, exposure, follow-up, and data collection	Page 6		

For peer review only

Participants	6	*(a) Cohort study* - Give the eligibility criteria, and the sources and methods of selection of participants. Describe methods of follow-up<br>*Case-control study* - Give the eligibility criteria, and the sources and methods of case ascertainment and control selection. Give the rationale for the choice of cases and controls<br>*Cross-sectional study* - Give the eligibility criteria, and the sources and methods of selection of participants<br><br>*(b) Cohort study* - For matched studies, give matching criteria and number of exposed and unexposed<br>*Case-control study* - For matched studies, give matching criteria and the number of controls per case	Page 6	RECORD 6.1: The methods of study population selection (such as codes or algorithms used to identify subjects) should be listed in detail. If this is not possible, an explanation should be provided.<br><br>RECORD 6.2: Any validation studies of the codes or algorithms used to select the population should be referenced. If validation was conducted for this study and not published elsewhere, detailed methods and results should be provided.<br><br>RECORD 6.3: If the study involved linkage of databases, consider use of a flow diagram or other graphical display to demonstrate the data linkage process, including the number of individuals with linked data at each stage.	Page 6
Variables	7	Clearly define all outcomes, exposures, predictors, potential confounders, and effect modifiers. Give diagnostic criteria, if applicable.	Page 6-7	RECORD 7.1: A complete list of codes and algorithms used to classify exposures, outcomes, confounders, and effect modifiers should be provided. If these cannot be reported, an explanation should be provided.	Page 6-7
Data sources/ measurement	8	For each variable of interest, give sources of data and details of methods of assessment (measurement).<br>Describe comparability of assessment methods if there is more than one group	Pages 6-7		

For peer review only

Bias	9	Describe any efforts to address potential sources of bias	Page 7		
Study size	10	Explain how the study size was arrived at	Pages 6-7		
Quantitative variables	11	Explain how quantitative variables were handled in the analyses. If applicable, describe which groupings were chosen, and why	Pages 6-7		
Statistical methods	12	(a) Describe all statistical methods, including those used to control for confounding<br>(b) Describe any methods used to examine subgroups and interactions<br>(c) Explain how missing data were addressed<br>(d) *Cohort study* - If applicable, explain how loss to follow-up was addressed<br>*Case-control study* - If applicable, explain how matching of cases and controls was addressed<br>*Cross-sectional study* - If applicable, describe analytical methods taking account of sampling strategy<br>(e) Describe any sensitivity analyses	Page 7-8		
Data access and cleaning methods		..		RECORD 12.1: Authors should describe the extent to which the investigators had access to the database population used to create the study population.<br><br>RECORD 12.2: Authors should provide information on the data	Pages 6-7

For peer review only

				cleaning methods used in the study.	
Linkage		..		RECORD 12.3: State whether the study included person-level, institutional-level, or other data linkage across two or more databases. The methods of linkage and methods of linkage quality evaluation should be provided.	-
**Results**					
Participants	13	(a) Report the numbers of individuals at each stage of the study (*e.g.*, numbers potentially eligible, examined for eligibility, confirmed eligible, included in the study, completing follow-up, and analysed)<br>(b) Give reasons for non-participation at each stage.<br>(c) Consider use of a flow diagram	Page 9	RECORD 13.1: Describe in detail the selection of the persons included in the study (*i.e.,* study population selection) including filtering based on data quality, data availability and linkage. The selection of included persons can be described in the text and/or by means of the study flow diagram.	Page 9
Descriptive data	14	(a) Give characteristics of study participants (*e.g.*, demographic, clinical, social) and information on exposures and potential confounders<br>(b) Indicate the number of participants with missing data for each variable of interest<br>(c) *Cohort study* - summarise follow-up time (*e.g.*, average and total amount)	Page 9		
Outcome data	15	*Cohort study* - Report numbers of outcome events or summary measures over time<br>*Case-control study* - Report numbers in each exposure	Page 9		

For peer review only

		category, or summary measures of exposure<br>*Cross-sectional study* - Report numbers of outcome events or summary measures			
Main results	16	(a) Give unadjusted estimates and, if applicable, confounder-adjusted estimates and their precision (e.g., 95% confidence interval). Make clear which confounders were adjusted for and why they were included<br>(b) Report category boundaries when continuous variables were categorized<br>(c) If relevant, consider translating estimates of relative risk into absolute risk for a meaningful time period	Pages 9-10		
Other analyses	17	Report other analyses done—e.g., analyses of subgroups and interactions, and sensitivity analyses	Page 11		
**Discussion**					
Key results	18	Summarise key results with reference to study objectives	Page 12		
Limitations	19	Discuss limitations of the study, taking into account sources of potential bias or imprecision. Discuss both direction and magnitude of any potential bias	Page 13-14	RECORD 19.1: Discuss the implications of using data that were not created or collected to answer the specific research question(s). Include discussion of misclassification bias, unmeasured confounding, missing data, and changing eligibility over time, as they pertain to the study being reported.	Page 13-14
Interpretation	20	Give a cautious overall interpretation of results	Pages 12-14		

For peer review only

		considering objectives, limitations, multiplicity of analyses, results from similar studies, and other relevant evidence			
Generalisability	21	Discuss the generalisability (external validity) of the study results	Pages 15		
**Other Information**					
Funding	22	Give the source of funding and the role of the funders for the present study and, if applicable, for the original study on which the present article is based	Pages 16-17		
Accessibility of protocol, raw data, and programming code		..	Page 17	RECORD 22.1: Authors should provide information on how to access any supplemental information such as the study protocol, raw data, or programming code.	Page 17

*Reference: Benchimol EI, Smeeth L, Guttmann A, Harron K, Moher D, Petersen I, Sørensen HT, von Elm E, Langan SM, the RECORD Working Committee. The REporting of studies Conducted using Observational Routinely-collected health Data (RECORD) Statement. *PLoS Medicine* 2015; in press.

*Checklist is protected under Creative Commons Attribution (CC BY) license.

For peer review only
